# Herpesviruses mimic zygotic genome activation to promote viral replication

**DOI:** 10.1038/s41467-025-55928-5

**Published:** 2025-01-16

**Authors:** Eva Neugebauer, Stephanie Walter, Jiang Tan, Nir Drayman, Vedran Franke, Michiel van Gent, Sandra Pennisi, Pia Veratti, Karla S. Stein, Isabelle Welker, Savaş Tay, Georges M. G. M. Verjans, H. T. Marc Timmers, Altuna Akalin, Markus Landthaler, Armin Ensser, Emanuel Wyler, Florian Full

**Affiliations:** 1 Institute of Virology, University Medical Center, and Faculty of Medicine, Albert-Ludwig-University Freiburg, Freiburg, Germany; 2 Spemann Graduate School of Biology and Medicine (SGBM), University of Freiburg, Freiburg, Germany; 3 Faculty of Biology, University of Freiburg, Freiburg, Germany; 4 Institute for Clinical and Molecular Virology, University Hospital Erlangen, Friedrich-Alexander-Universität Erlangen-Nürnberg, 91054 Erlangen, Germany; 5 The Department of Molecular Biology and Biochemistry, the Center for Virus Research and the Center for Complex Biological Systems, The University of California, Irvine, Irvine, CA 92697 USA; 6 Berlin Institute for Medical Systems Biology, Max Delbrück Center for Molecular Medicine, Helmholtz Society, Berlin, Germany; 7 HerpesLabNL, Department of Viroscience, Erasmus Medical Center, Rotterdam, The Netherlands; 8 The Pritzker School for Molecular Engineering, The University of Chicago, Chicago, IL 60637 USA; 9 German Cancer Consortium (DKTK), partner site Freiburg, a partnership between the DKFZ and Medical Center-University of Freiburg, and Department of Urology, Medical Center-University of Freiburg, Freiburg, Germany; 10 German Consulting Laboratory for HSV and VZV, Medical Center - University of Freiburg, Freiburg, Germany

**Keywords:** Herpes virus, Epigenetics

## Abstract

Zygotic genome activation (ZGA) is crucial for maternal to zygotic transition at the 2-8-cell stage in order to overcome silencing of genes and enable transcription from the zygotic genome. In humans, ZGA is induced by DUX4, a pioneer factor that drives expression of downstream germline-specific genes and retroelements. Here we show that herpesviruses from all subfamilies, papillomaviruses and Merkel cell polyomavirus actively induce DUX4 expression to promote viral transcription and replication. Analysis of single-cell sequencing data sets from patients shows that viral DUX4 activation is of relevance in vivo. Herpes-simplex virus 1 (HSV-1) immediate early proteins directly induce expression of DUX4 and its target genes, which mimics zygotic genome activation. Upon HSV-1 infection, DUX4 directly binds to the viral genome and promotes viral transcription. DUX4 is functionally required for infection, since genetic depletion by CRISPR/Cas9 as well as degradation of DUX4 by nanobody constructs abrogates HSV-1 replication. Our results show that DNA viruses including herpesviruses mimic an embryonic-like transcriptional program that prevents epigenetic silencing of the viral genome and facilitates herpesviral gene expression.

## Abstract (web summary)

Neugebauer et al. show activation of the usually silenced embryonic factor DUX4 by herpesviruses in vitro and in patients, and demonstrate that depletion of DUX4 by nanobody degraders abrogates viral replication.

## Introduction

Herpesviruses are a major health burden worldwide, with a prevalence of 25 – 100% dependent on the virus species and geography^1,2^. Herpesviruses cause a number of prevalent diseases, like oral and genital herpes, chickenpox, shingles, infectious mononucleosis and encephalitis. Life-threatening infections in healthy humans are rare, however severe disease is a significant risk in e.g. immunocompromised patients, transplant recipients or newborns^3,4^. Some herpesviruses such as Epstein-Barr virus (EBV) and Kaposi’s sarcoma-associated herpesvirus (KSHV) are also known to be the causative agent of particular cancer types^5^. Due to their lifelong persistence in the host, the risk of reactivation and developing a lytic infection is constantly present. Treatment options are limited and therapies able to eliminate the virus from the body are unavailable. A better understanding of the mechanisms involved during herpesviral infection and persistence is therefore essential.

In a recent study, we observed that the upregulation of the cellular protein Tripartite-motif 43 (TRIM43) upon HSV-1 infection is dependent on the embryonic transcription factor DUX4^6^, which was also confirmed by Friedel et al.^7^. DUX4 is a germline transcription factor exclusively expressed during the 2-cell to the 8-cell state of embryogenesis, a short phase during human embryonic development^8–10^. There, DUX4 is critical for zygotic genome activation (ZGA), a transcriptional activation event that leads to the induction of hundreds of target genes and allows the embryo to proceed further in development^11,12^. Concomitantly, it induces transcription from retroelements such as LTRs and ERVs^9,10,13^ which function as alternative promotors, to regulate ZGA-related genes and mediate pluripotency^14,15^. After this limited period of activation, DUX4 expression is silenced throughout all adult tissues, except for spermatocytes^16^. An exception to this is the genetic disorder Facioscapulohumeral Muscular Dystrophy (FSHD). In FSHD, DUX4 silencing is lost and aberrant DUX4 expression in adult muscle cells leads to apoptosis and degeneration of the affected muscles^17,18^. The *DUX4* gene resides within the macrosatellite repeat array D4Z4^19–22^, that harbors up to 100 copies of DUX4, with only the most distal copy encoding for a protein^17^. During early embryonic development, this macrosatellite array is transformed into heterochromatin after the 8-cell stage^8,10^ and its repression is strictly controlled by chromatin regulatory factors and repressors which have not been fully investigated^16,19^.

Given the peculiar expression pattern of DUX4 and its upregulation in herpesviral infection, we aimed to determine whether DUX4 plays a role during DNA virus infection and to shed light on its potential functions. We show that DUX4 expression is a general mechanism that is conserved among several DNA viruses and can also be observed in patient samples. For HSV-1, we could demonstrate that the viral proteins ICP0 and ICP4 induce DUX4 expression and of hundreds DUX4 target genes, and that DUX4 is essential for HSV-1 and HSV-2 replication. This points at so far unknown endogenous functions of DUX4 and extends our knowledge of this unique transcription factor.

## Results

**Fig. 1:**
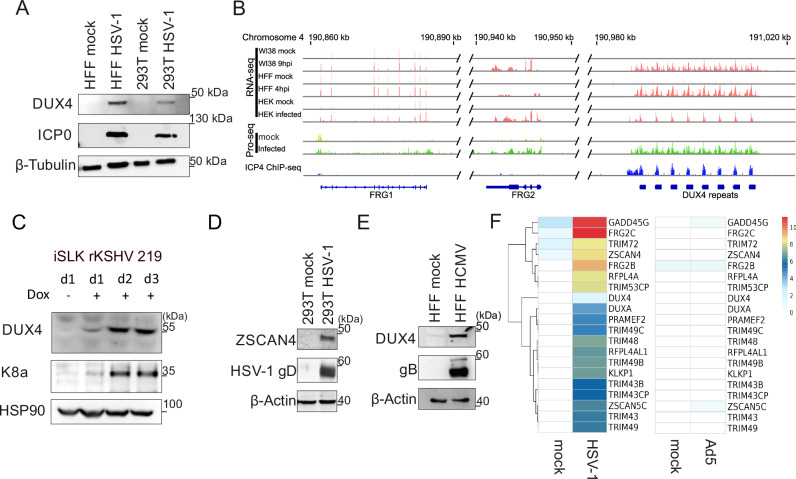
DUX4 induction after herpesviral infection. **A** Western blot of primary HFF cells infected with HSV-1 for 24 h and 293T cells infected with HSV-1 for 18 h, both at a MOI 5. ICP0 was used as marker for infection. Representative experiment out of *n* = 3. **B** RNA-seq. data of the *DUX4* and neighboring *FRG1* and *FRG2* loci in WI38 9 hpi, HFF 4 hpi and HEK 293T cells 18 hpi. The Precision Run-On Sequencing (Pro-Seq) data show RNA pol II occupancy on the *FRG1*, *FFRG2* and *DUX4* locus in infected and uninfected cells. ICP4 ChIP-Seq data show ICP4 occupation at the *DUX4* locus in cells infected with HSV-1. **C** Western blot of iSLK rKSHV 219 cells induced with 1 µg/ml Doxycycline for 1, 2 or 3 days. K8α run on a different gel and was used as control for KSHV reactivation. Representative experiment out of *n* = 3. **D** Western blot of 293T cells infected with HSV-1 for 18 h at a MOI of 5 and analyzed for expression of ZSCAN4. Glycoprotein D (gD) served as a control for viral infection. Representative experiment out of *n* = 3. **E** HFF cells infected with HCMV for 6 d with MOI 1. Western blot analysis of DUX4. HCMV glycoprotein B (gB) was used as marker for infection. Representative experiment out of *n* = 3. **F** RNA-seq. analysis of DUX4 target genes after HSV-1 or Ad5 infection. Ad5 RNA-seq. data was taken from BioProject PRJEB57806. Source Data are provided as a Source Data file.

We confirmed the expression of DUX4 in various cell lines infected with Herpes-simplex virus 1 (HSV-1) by Western Blot and reanalysis of published RNA-seq. datasets (Fig. 1A, B)^6,23–26^. Comparison of three independent RNA-seq. datasets in three different cell types showed no expression of DUX4 and DUX4 target genes (e.g. FRG1) in uninfected cells (Fig. 1B). Upon infection with HSV-1, however, transcription of *DUX4* is induced as well as known DUX4 target genes (Fig. 1B). Interestingly, we also confirmed the expression of HSAT-II satellite repeat transcripts, whereas the expression of other satellite repeats remained unchanged (Fig. S1A, B). HSAT-II repeats are known DUX4 targets^10,27^ and have recently been shown to play a role in HCMV replication^28^. Moreover, the reanalysis of a published Precision Run-On sequencing (PRO-seq.) dataset^25^ showed no RNA-polymerase-II occupancy at the DUX4 locus in uninfected cells, indicating that the locus is completely silenced under normal conditions (Fig. 1B). In contrast, HSV-1 infection induced a robust PRO-seq. signal at the DUX4 locus. DUX4 mRNA expression was also induced upon infection of primary human melanocytes with HSV-1, emphasizing the physiological relevance of DUX4 induction (Fig. S1C). Western Blot as well as qRT-PCR experiments further confirmed the expression of DUX4 and known DUX4-target genes^29^ TRIM48, TRIM49, ZSCAN4, ZSCAN5a, ZSCAN5d and RFPL4A upon HSV-1, HCMV and KSHV-infection (Fig. 1D, E, S1D, S2A) in different experimental settings. This indicates that DUX4 is functional and acts as a transcriptional activator in herpesvirus infected cells. Reanalysis of published RNA-seq. datasets could not confirm expression of DUX4 in cells infected with other RNA-viruses, Adenoviruses or Poxviruses (both DNA viruses) (Fig. 1F, S2I). Taken together, these data show that the induction of DUX4 expression is a common feature of human herpesviruses.

**Fig. 2:**
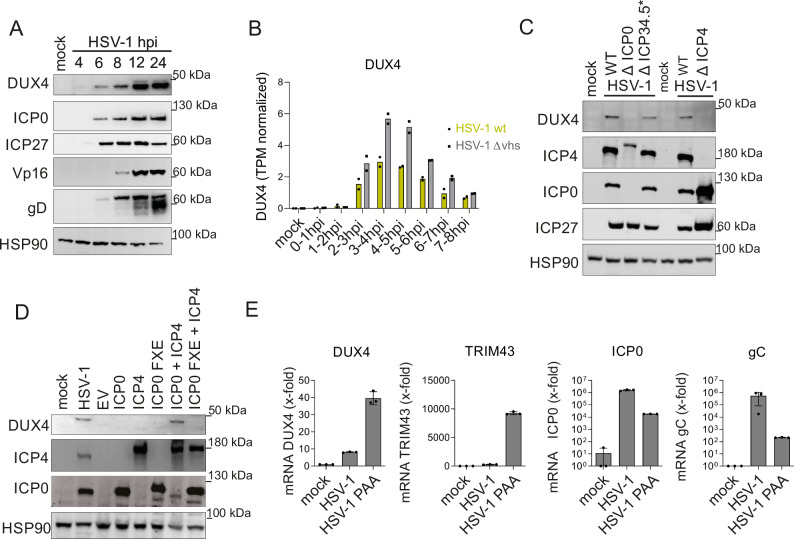
Herpesviral immediate-early proteins induce DUX4 expression. **A** Western Blot of DUX4 and HSV-1 proteins ICP0, ICP27, VP16 and glycoprotein D (gD) in HFF cells infected with HSV-1 harvested at different hpi (MOI of 10). Representative experiment out of *n* = 3. **B** *DUX4* expression kinetics of newly synthesized RNA (4su-sequencing) in HFF cells infected with HSV-1 wt and HSV-1 delta vhs virus. Reanalysis of data from Friedel et al.^7^. **C** Western Blot of DUX4 and HSV-1 proteins in primary HFF infected with HSV-1 and HSV-1 mutants (HSV-1 deltaICP0 (ICP4-YFP), HSV-1 deltaICP27, HSV-1 deltaICP34.5/ICP47) harvested at 16 hpi (MOI 10). One representative experiment out of *n* = 5. **D** Western blot analysis of DUX4 and HSV-1 protein in 293T cells transfected with HSV-1 IE proteins ICP0, ICP0 FXE, ICP4 (ICP4-YFP), ICP0 + ICP4 (ICP4-YFP) and ICP0 FXE + ICP4 (ICP4-YFP) for 48 h or infected with HSV-1 for 18 h (MOI of 10). ICP0 FXE is a mutant with a deletion in the RING domain, which inhibits Ubiquitin E3 ligase activity. EV: empty vector control. One representative experiment out of *n* = 3. **E** qRT-PCR analysis of cellular genes *DUX4*, *TRIM43* as well as the viral genes *ICP0* and *gC* in HDF-TERT cells untreated or treated with PAA and infected with HSV-1 (MOI of 0.1). Values are biological replicates and presented as mean fold induction +/-SD (normalized to HPRT RNA) relative to uninfected control cells. Source Data are provided as a Source Data file.

Next, we wanted to address the significance of herpesviral DUX4 induction for the infection itself, since DUX4 is a germline transcription factor that is not expressed in healthy adult tissue^8,10^. We first hypothesized that the induction of DUX4 might be part of an antiviral response of the cell to viral infection triggered by a herpesviral pathogen associated molecular pattern (PAMP), or by herpesviral induction of a cellular DNA-damage response (DDR). We reanalyzed several published RNA-seq. datasets, investigating DUX4 mRNA expression in response to cellular changes, but found no evidence for DUX4 expression. In addition, triggering a DNA-damage response by treating cells with Bleocin or Etoposide did not induce DUX4 protein expression (Fig. S3A). The only evidence for DUX4 expression came from a Chromatin-IP-sequencing (ChIP-Seq) dataset^26^ investigating the binding of the HSV-1 infected cell protein 4 (ICP4) to the cellular genome. ICP4 is a potent activator of viral transcription, part of the viral tegument, and essential for replication of HSV-1^30,31^. Our reanalysis of this dataset showed a very strong binding of the ICP4 protein to the DUX4 locus (Fig. 1B). Of note, the peak intensity of ICP4 binding at the DUX4 locus was among the highest in the entire host genome. This ICP4 binding was a strong indication for an active induction of DUX4 by HSV-1 infection. Moreover, experiments with phosphonoacetic acid (PAA), an inhibitor of the viral DNA-polymerase, showed that herpesviral DNA replication is dispensable for DUX4 expression (Fig. S3B), indicating that induction of the DUX4 gene takes place at the immediate-early stage of the viral gene expression cascade. Even after infection with UV-inactivated virus, DUX4 protein was still induced, indicating that incoming components of the virion contribute to DUX4 induction (Fig. S3B). Analysis of the kinetics of DUX4 expression upon HSV-1 infection further demonstrated that DUX4 protein could be detected as early as 4 h post infection with protein levels constantly increasing over the course of infection (Fig. 2A). We analyzed the expression kinetics of newly synthesized DUX4-mRNA in the context of HSV-1 infection by reanalyzing 4-thiouridine (4su)-sequencing datasets from Friedel et al.^7^. DUX4-mRNA shows a peak from 2-6 h post infection (Fig. 2B). To elucidate the involvement of HSV-1 tegument proteins in the induction of DUX4 expression we performed infection experiments with HSV-1 mutants lacking the immediate early proteins ICP0, ICP4 and ICP34.5. We could show that wildtype (wt) HSV-1 infection as well as infection with HSV-1-Δ γ34.5 resulted in DUX4 protein expression, whereas DUX4 expression is abrogated in cells infected with HSV-1 lacking either ICP0 (expressing ICP4-YFP protein) or ICP4 (Fig. 2C and Fig. S3C). In addition, the transient co-expression of ICP0 and ICP4 is sufficient to induce DUX4 expression in the absence of viral infection (Fig. 2D), whereas an E3-ubiquitin-ligase deficient mutant of ICP0 (ICP0 FXE) was not able to induce expression of DUX4 when coexpressed together with ICP4 (Fig. 2D). Although U2OS cells support replication of a HSV-1 lacking ICP0, DUX4 is not expressed in uninfected U2OS cells (Fig. S3D). Moreover, DUX4 overexpression is not sufficient to complement replication of a HSV-1 lacking ICP0 in HFF cells (Fig. S3E). Taken together, this confirms that ICP0 and ICP4 are necessary and sufficient for inducing DUX4 expression and indicates that DUX4 is only briefly activated by ICP0 and ICP4 during the early phase of infection. To test this, we treated cells with PAA, which arrests viral infection at the stage of viral DNA replication, i.e. after immediate-early gene expression but before late gene expression (Fig. 2E and Fig. S4A, B). PAA treated cells showed a strong increase in expression of *DUX4* mRNA and its target genes by qRT-PCR analysis of HDF cells infected with HSV-1 (Fig. 2E), as well as the DUX4 protein by WB analysis in 293T cells (Fig. S3B), supporting the notion of a transient activation of DUX4 during the early stages of HSV-1 infection.

**Fig. 3:**
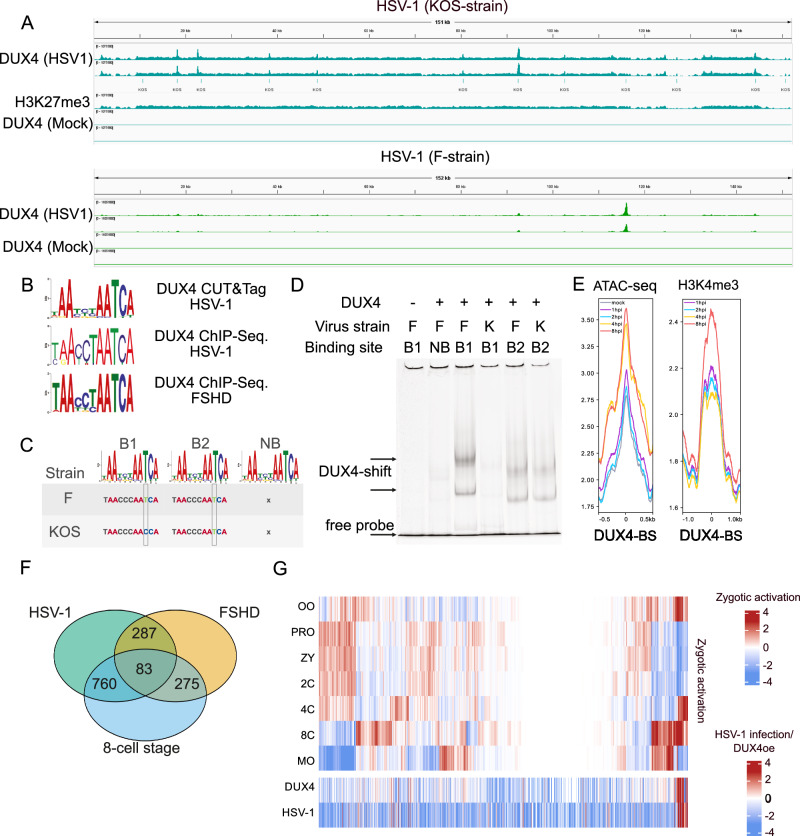
Direct binding of DUX4 to the HSV-1 genome. **A** CUT&RUN with a DUX4-specific antibody in HFF1 cells infected with HSV-1 at 12 hpi performed in duplicates. One sample with an H3K27me3-specific antibody was included as control. Data was normalized to Drosophila Spike-in DNA. Lines labeled with KOS indicate the DUX4 motif sites in the HSV-1 genome determined with FIMO. **B** Comparison of DUX4 consensus binding sequence from DUX4 CUT&Tag in HSV-1 infected cells (upper panel), DUX4 HSV-1 ChIP-Seq (middle panel) and with the previously published DUX4 consensus sequences (Geng et al.) in the lower panel. **C**, **D** Electrophoretic Mobility Shift Assay (EMSA) of different 600 bp fragments of fluorescently labelled viral DNA containing either one DUX4 binding motif (B1/B2) or no DUX4 binding motif (NB), incubated with purified DUX4 protein (DUX4). B1 shows a T- > C nucleotide exchange at position 9 of the DUX4 binding motif in the KOS strain. Representative experiment out of *n* = 4. **E** Reanalysis of ATAC-seq and histone modifications at known DUX4 binding sites of THP-1 cells infected with HSV-1 at 1 h, 2 h, 4 h and 8 h post infection. Histone modification and ATAC-seq were taken from Gao et al. and Hennig et al. respectively^33,34^ and DUX4 binding sites from the DUX4 ChIP-Sequencing after HSV-1 infection. **F** Venn diagram showing overlap of genes that are significantly upregulated in the 8-cell stage of human development, HSV-1 infection and FSHD. **G** Expression pattern of embryonic genes during embryonic genome activation compared to expression pattern in DUX4 overexpressing and HSV-1 infected cells. Embryonic data is normalized to the average expression value of each gene. Data from DUX4 overexpression and HSV-1 infection is relative to mock. Shown are genes which have DUX4 binding sites in the proximity of the TSS (500 bp downstream, 1000 bp upstream of the annotated promoter). (OO: oozyte, PRO: pronucleus, ZY: zygote, 2 C: 2-cell state, 4 C: 4-cell state, 8 C: 8-cell state, MO: morula). Source Data are provided as a Source Data file.

It is known that the physiological function of DUX4 during embryonic genome activation at the 2-8 cell stage is to directly bind to DNA and activate genes and retroelements that are necessary for developmental progression^9,10^. In particular, endogenous retroelements act as promoters for downstream genes, and binding of DUX4 to retroelements activates transcription of respective genes^14,32^. In order to analyze the role of DUX4 in herpesviral replication, we performed endogenous DUX4 ChIP-seq. DUX4 CUT&Tag as well as DUX4 CUT&RUN experiments. The DUX4-ChIP conditions optimized for DUX4 binding to the cellular genome resulted in a high background for the viral genome, most likely due to different physical properties that affect sonication and differences in chromatin accessibility. However, using CUT&Tag and CUT&RUN we could detect direct binding of DUX4 to the genome of HSV-1 GFP (F-strain) starting at about 4 h post infection (Fig. 3A, S4C,D, Supplementary Data 1, 2). We observed several DUX4 binding sites over the HSV-1 genome, with the biggest peak located in between the UL55 and UL56 genes. The DUX4 binding site between UL55 and UL56 was not present in many other HSV-1 strains like KOS, however, binding of DUX4 to other peaks was conserved (Fig. 3A,C,D, Fig. S4D, Supplementary Data 3). Analysis of the binding sites from our endogenous DUX4 ChIP-seq. CUT&Tag and comparison with the DUX4 ChIP-Seq. performed by Young et al. ^14^ with overexpressed DUX4 protein in muscle cells showed almost identical consensus binding sites (Fig. 3B). A computational analysis found multiple potential DUX4 consensus motifs in the genomes of HSV-1, HSV-2, HCMV, EBV and KSHV (Supplementary Data 4), suggesting that DUX4 possibly binds to viral genomes of all herpesviruses. Next, we established an electrophoretic mobility shift assay (EMSA) to confirm binding of DUX4 to regions of the HSV-1 genome (Fig. 3D). To this end, full-length DUX4 protein was expressed in bacteria (Fig. S5B), purified and about 600bp-long fragments of the HSV-1 genome amplified by PCR. After coincubation of recombinant DUX4 with fluorescently labeled PCR fragments we observed binding of DUX4 to a fragment containing one copy of the consensus DUX4 DNA-binding motif, whereas the exchange of T for a C at position 9 completely abrogated binding (Fig. 3D). EMSA experiments for KSHV confirmed binding of DUX4 to 3 predicted KSHV binding sites (Fig. S5A). For the host genome, the ChIP-Seq. and the CUT&Tag experiments revealed about 11.000 DUX4 binding sites with ChIP-Seq. and 3700 DUX4 binding sites with CUT&Tag mapping to the host genome, which is comparable to DUX4 ChIP-seq. data from Geng et al. in muscle cells^32^. Most DUX4 binding sites were not at transcriptional start sites or within exon regions of genes, but within intronic and intergenic regions. We reanalyzed HSV-1 ATAC-seq. and histone modification data from Hennig et al.^33^ and Gao et al.^34^, respectively, in order to find out whether cellular chromatin becomes more accessible in the course of HSV-1 infection at known DUX4 binding sites. ATAC-seq. as well as histone 3-lysine 4 (H3K4me3) methylation peaks increase over the course of infection (Fig. 3E) at DUX4 binding sites (Supplementary Data 5). Moreover, a comparison of upregulated genes from HSV-1 infection, 8-cell stage of human development and FSHD patients showed that 843 host genes are significantly induced both during herpesviral infection and during the 8-cell stage of early human development (Fig. 3F, G and Supplementary Data 6-8)^35,36^.

**Fig. 4:**
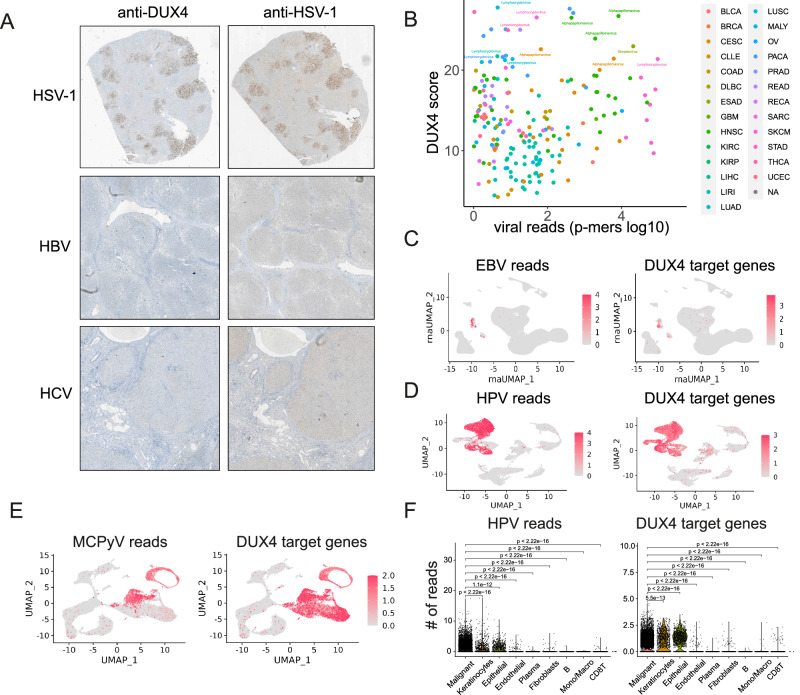
Viral DUX4 expression in patient samples. **A** Immunohistochemistry staining of liver biopsies from hepatitis patients with HSV-1, HBV and HCV. Consecutive slices were stained with antibodies specific for DUX4 (left panel) and HSV-1 (right panel). **B** Reanalysis of sequencing datasets from the International Cancer Genome Consortium (ICGC). Datasets were classified according to a DUX4 score (Expression of DUX4 and DUX4-target genes, *y*-axis) and viral abundances (x-axis). Bladder Urothelial Carcinoma (BLCA), Breast invasive carcinoma (BRCA), Cervical squamous cell carcinoma and endocervical adenocarcinoma (CESC), Chronic Lymphocytic Leukemia (CLLE), Colon adenocarcinoma (COAD), Diffuse large B cell lymphoma (DBLC), Esophageal Adenocarcinoma (ESAD), Glioblastoma multiforme (GBM), Head and Neck squamous cell carcinoma (HNSC), Kidney renal clear cell carcinoma (KIRC), Kidney renal papillary cell carcinoma (KIRP), Liver hepatocellular carcinoma (LIHC), Liver Cancer (LIRI), Lung adenocarcinoma (LUAD), Lung squamous cell carcinoma (LUSC), Malignant Lymphoma (MALY), Ovarian serous cystadenocarcinoma (OV), Pancreatic Cancer (PACA), Prostate adenocarcinoma (PRAD), Rectum adenocarcinoma (READ), Renal Cancer (RECA), Sarcoma (SARC), Skin Cutaneous Melanoma (SKCM), Stomach adenocarcinoma (STAD), Thyroid carcinoma (THCA), Uterine Corpus Endometrial Carcinoma (UCEC). **C** Reanalysis of single cell sequencing datasets from patients with EBV-positive Nasopharynx carcinoma (NPC) from Liu et al.^37^. Left panel: UMAP projection of number of EBV-specific reads per cell, pool of 10 donors. Right panel: UMAP projection of number of DUX4-target gene specific reads pool of 10 donors. **D** Reanalysis of single cell sequencing datasets from patients with HPV-positive head and neck cancer from Kürten et al.^38^. Left panel: UMAP projection of number of HPV-specific reads per cell, pool of 10 donors, 5 donors HPV-positive and 5 donors HPV-negative. Right panel: UMAP projection of number of DUX4-target gene specific reads, pool of 10 donors, 5 donors HPV positive and 5 donors HPV-negative. **E** Reanalysis of single cell sequencing datasets from patients with Merkel cell polyomavirus (MCPyV) from Das et al.^39^. Left panel: UMAP projection of number of MCPyV-specific reads per cell, pool of 11 donors. Right panel: UMAP projection of number of DUX4-target gene specific reads, pool of 11 donors. **F** Quantification of DUX4-reads and HPV-reads in single cells in cell clusters from D. Statistical analysis was done using the two-sided Kruskal-Wallis test with adjustment for multiple comparison.

Next, we wanted to address whether DUX4 expression is also of relevance in vivo. Immunohistochemistry confirmed DUX4 expression in liver biopsies from HSV-1 hepatitis patients but not in hepatitis samples from hepatitis-B virus or in hepatitis-C virus infected liver samples (Fig. 4A, Fig. S5C). In addition, we wanted to investigate whether additional DNA viruses, in particular DNA viruses that are also able to establish persistent infections like herpesviruses also show DUX4 expression. To this end, we reanalyzed sequencing data from the International Cancer Genome Consortium and classified tumors for DUX4 expression by a DUX4 score that includes expression of *DUX4* and known DUX4 target genes. Then we correlated this with the presence of viral reads in the tumor samples. We found a strong association between *DUX4* expression and human papillomaviruses (HPV) in head and neck cancer samples as well as an association with EBV-positive nasopharyngeal carcinoma (Fig. 4B). To confirm this, we reanalyzed single cell RNA-seq. data from patients with EBV-positive nasopharyngeal carcinoma^37^, and could demonstrate that *DUX4* and DUX4 target genes are only expressed in EBV-positive tumor cells but not in healthy tissue from the same patients (Fig. 4C). In addition, we could detect expression of *DUX4* and DUX4 target genes in single cell RNA-seq. datasets from patients with HPV- positive head and neck cancer^38^(Fig. 4D, F) as well as single-cell sequencing datasets from Merkel cell polyomavirus (MCPyV)-positive Merkel cell carcinoma^39^ (Fig. 4E). In contrast to alpha-herpesviruses, the default outcome of infection of cells with gamma-herpesvirus KSHV and EBV is not lytic replication but latency. Therefore, we asked whether DUX4 alone is sufficient to induce lytic replication upon overexpression in latently infected cells. Using a doxycycline-inducible system, we overexpressed DUX4 in latently infected Raji cells (EBV). DUX4 overexpression alone was not sufficient to trigger lytic gene expression (Fig. S5D), indicating that DUX4 is not sufficient to induce EBV reactivation.

**Fig. 5:**
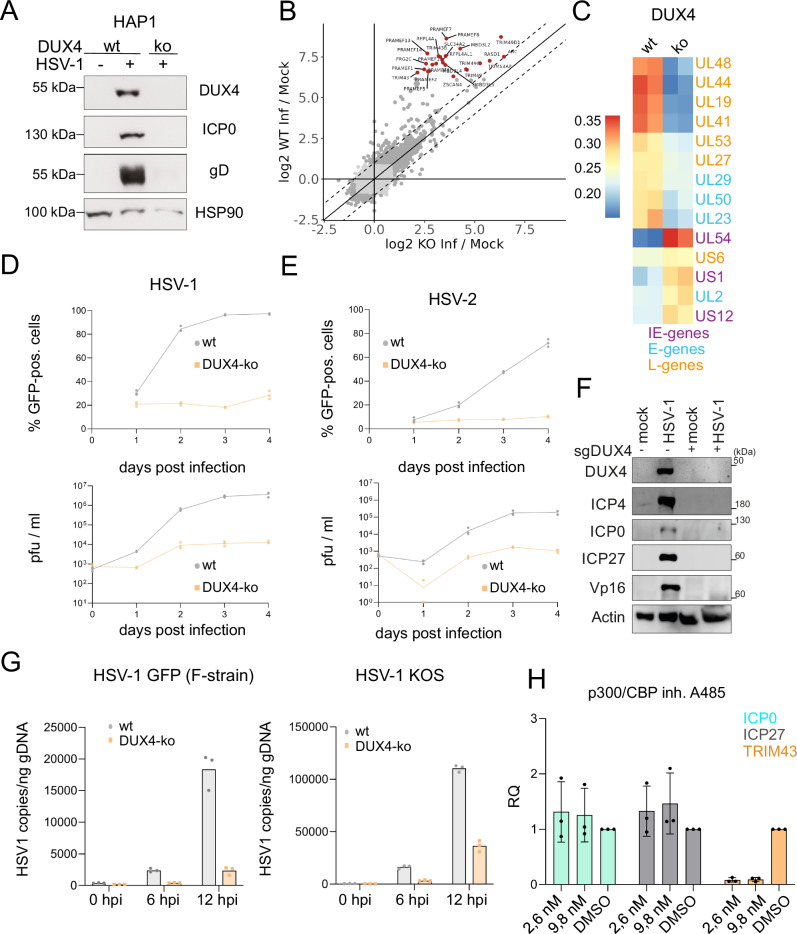
DUX4 expression is critical for HSV-1 and HSV-2 replication. **A** DUX4 ko HAP1 cells were generated with CRISPR/Cas9 and DUX4 specific sgRNAs. HSV-1 protein expression in HAP1 wt cells and HAP1 cells with DUX4 knockout. Cells were infected with HSV-1-GFP (MOI of 2) for 20 h and analyzed by western blot. HSP90 was used as loading control. Representative experiment out of *n* = 3. **B** mRNA expression (RNA-seq.) of all host genes of HAP1-DUX4-ko cells plotted against HAP1 wt cells, both infected with HSV-1-GFP (MOI of 1) for 8 h. Depicted in red are known DUX4 target genes. **C** Heatmap of mRNA-expression of selected viral transcripts from HSV-1 (MOI of 1) infected wt and DUX4 ko HAP1 cells 8 h post infection. Marked are viral genes with immediate-early, early and late expression kinetics. **D** GFP-expression of HAP1 wt and HAP1 DUX4-ko cells infected with HSV-1-GFP (MOI of 0.1) at day 1–4 post infection, measured by flow cytometry (upper panel). In parallel, viral titers were assessed by plaque assays on Vero cells (lower panel). One representative experiment out of *n* = 3. Values shown are technical replicates. **E** GFP-expression of HAP1 wt and HAP1 DUX4-ko cells infected with HSV-2-GFP (MOI of 0.05) at day 1–4 post infection, measured by flow cytometry (upper panel). In parallel, viral titers were assessed by plaque assays on Vero cells (lower panel). One representative experiment out of *n* = 3. Values shown are technical replicates. **F** Western blot of 293T WT cells and 293T DUX4-ko cells infected with HSV-1 for 18 h. Comparison of HSV-1 protein expression in wt cells and cells with complete DUX4-ko. Actin was used as loading control. Representative experiment out of *n* = 4. **G** qRT-PCR analysis of the replication of HSV-1 GFP (F-strain) and HSV-1 KOS at 0,6 and 12 h after infection of HAP1 wt and HAP1 DUX4-ko cells (MOI of 1). Representative experiment out of *n* = 3. Values shown are technical replicates. **H** qRT-PCR analysis of the DUX4 target genes TRIM43 as well as the viral genes ICP0 and ICP27 in cells treated with 2,6 nM, 9,8 nM A485 or DMSO and infected with HSV-1 (MOI of 3). Values shown are the biological replicates (*n* = 3) and presented as mean fold induction +/- SD (normalized to HPRT RNA) relative to uninfected control cells. Source Data are provided as a Source Data file.

Our data revealed a robust expression of DUX4 during lytic replication of all human herpesvirus subfamilies. We thus wanted to address the physiological consequences concerning herpesviral gene expression and replication. DUX4 is located within the 4q35 region of chromosome 4^20,22^ and it is known in the field that the gene is particularly complicated to target by CRISPR/Cas9. In healthy individuals, the locus consists of 10-100 repeat units, and a copy of DUX4 resides in every unit. However, only one of the D4Z4 repeat arrays, the most distal one adjacent to the telomeres is encoding for the functional protein^17^. In addition to multiple repeats on chromosomes 4 (4q allele) there is also another heterochromatic repeat array on chromosome 10 (10q allele)^21^, which is likely to interfere with CRISPR/CAS9 based knockout strategies^40^. We finally managed to generate knockout cells in the haploid human cell line HAP1 (Fig. 5A). Upon infection of HAP1 DUX4 knockout (ko) cells, we could not detect DUX4 protein by Western Blot, while as expected it is present in the infected wildtype (wt) HAP1 cells. Interestingly, the expression of the viral proteins ICP0 and glycoprotein D (gD) was also strongly reduced in DUX4 ko cells compared to wt cells. In order to assess the effect of *DUX4* ko on the transcription of the entire viral genome, we performed RNA-seq. from HSV-1 infected knockout and wt cells 8 h post infection. Comparison of host gene expression from DUX4-wt and -ko cells showed that DUX4 target genes are significantly downregulated in the ko cells, whereas transcription of other genes is not altered (Fig. 5B). Unsupervised clustering showed altered expression of most HSV-1 genes in ko cells compared to wt cells (Fig. 5C, S6A). Whereas the expression of early and late genes is higher in wt cells at 8 h post infection, the expression of immediate-early genes like *UL54* or *US1* is lower in the wt and higher in the ko cells, indicating that DUX4 is required for progression to the later stages of HSV-1 infection (Fig. 5C, S6B). Infection experiments with GFP-expressing HSV-1 and HSV-2 showed that the infection does not proceed in DUX4 ko cells compared to wt cells in which the virus replicates at normal levels (Fig. 5D, E). We also used a transient approach in 293T cells to knockout *DUX4* at the population level, which resulted in a complete knockout of DUX4 for a short time period. Infection of DUX4 ko 293T cells resulted in an almost complete loss of most HSV-1 genes tested in Western Blot, like ICP0, ICP4, ICP27 and VP16 compared to wt 293T cells (Fig. 5F), confirming that DUX4 is critical for HSV-1 replication. Western Blots in HAP1 cells however showed comparable levels of ICP27 protein in wt and DUX4-ko cells, indicating cell type specific differences (Fig. S6B). Analysis of HSV-1 replication kinetics showed reduced DNA replication of HSV-1 in the absence of DUX4 compared to wt cells (Fig. 5G). Of note, the difference in DNA replication exists for both the KOS- and the F-strain. This shows that the binding site between UL55 and UL56, which is not conserved between KOS- and F-strain (Fig. 3A), is not important for HSV-1 replication. DUX4 is known to interact with the histone acetyltransferases p300/CBP and thereby promote transcription of DUX4 target genes^41^. To investigate involvement of p300/CBP in transcriptional activation of viral genes, we treated HSV-1 infected cells with A485, an inhibitor of p300/CBP. As expected, expression of TRIM43 was reduced upon A485 treatment compared to DMSO control, whereas expression of ICP0 and ICP27 was unchanged (Fig. 5H).

**Fig. 6:**
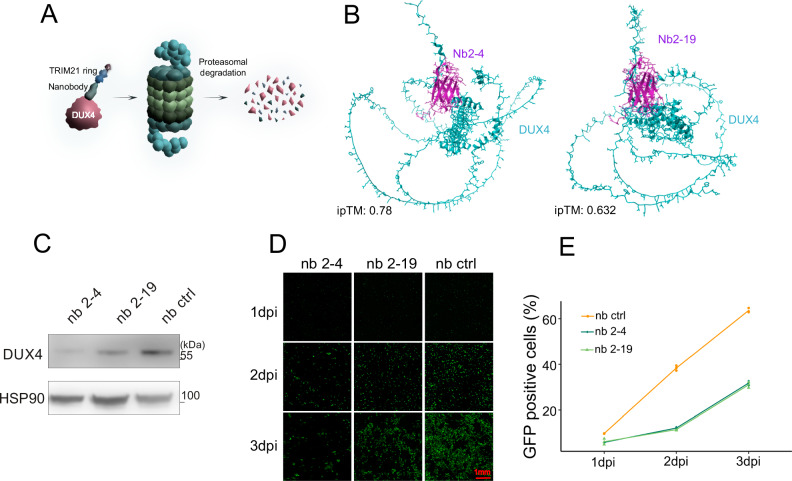
Degradation of DUX4 during HSV-1 infection using the mini-TRIMAway assay. **A** Principle of the mini-TRIMAway assay: A DUX4 specific nanobody linked to the TRIM21 ring targets DUX4 for proteosomal degradation. **B** AlphaFold-Multimer generated prediction of the interaction of the two DUX4 directed nanobody clones Nb2-4 and Nb2-19 to the DUX4 protein. **C** Western blot analyzing DUX4 levels after simultaneous induction of DUX4 and induction of the nanobody targeting DUX4 for degradation (nb 2–4 or nb 2–19) or control nanobody (nb ctrl). HEK293T were induced with 0,1 µg/ml Doxycycline and analyzed 24 h post induction. HSP90 was used as loading control. **D** Microscope analysis of the mini-TRIMAway assay for DUX4 degradation after HSV-1 infection at day 1–3 post infection. The corresponding nanobody (nb 2-4, nb 2-19 or nb ctrl) was induced with 2 µg/ml doxycycline and infected with HSV-1 GFP (MOI 0.05). Representative experiment out of *n* = 3. **E** Measurement of GFP-expression by flow cytometry of the mini-TRIMAway assay for DUX4 degradation after HSV-1 infection at day 1-3 post infection. The corresponding nanobody (nb 2–4, nb 2–19 or nb ctrl) was induced with 2 µg/ml doxycycline and infected with HSV-1 GFP (MOI 0.05). Representative experiment out of *n* = 3. Values shown are technical replicates. Source Data are provided as a Source Data file.

In order to confirm the results from our DUX4 ko cells, we generated DUX4 specific nanobodies using the purified DUX4 protein (Fig. S5B). We used two DUX4-specific nanobodies to generate mini-TRIMAway constructs in order to target DUX4 at the protein level. The TRIMAway technique is based on the intracellular E3-ubiquitin ligase TRIM21 which binds to the Fc-part of antibodies and thereby mediates proteasomal degradation of an antibody-antigen complex^42,43^. The mini-TRIMAway assay relies on direct fusion of the E3-ligase domain of TRIM21 to the nanobody (Fig. 6A). Computational modeling with AlphaFold confirmed binding of nanobodies 2–4 and 2–19 to DUX4 (Fig. 6B). Intracellular expression of two DUX4-specific mini-TRIMAway construct mediated degradation of overexpressed DUX4 compared to a GFP-specific control nanobody (Fig. 6C). Expression of two DUX4-specific mini-TRIMAway constructs prevents spread of HSV-1 replication in a low MOI infection setting with a GFP-expressing HSV-1 compared to a control mini-TRIMaway construct as shown by immunofluorescence (Fig. 6D) and flow cytometry (Fig. 6E). This confirms our results from the ko-cells and demonstrates the importance of DUX4 for herpesviral replication.

## Discussion

In humans the female oocyte is produced during female gametogenesis in the embryo and is stored in prophase I of the meiosis for up to 50 years. Oocyte transcription is halted by epigenetic mechanisms, and stored mRNAs mostly control development^44^. After fertilization the zygote is formed, maternal to zygotic transition (MZT) takes place and with the onset of ZGA the zygotic genome starts to be transcribed into zygotic mRNA^44^. In order to activate transcription, the embryo has to overcome silencing of the genome, which is regulated by repressive epigenetic modifications like DNA methylation, histone modifications and a shortage of the cellular transcription machinery^44,45^. DUX4 has been shown to be important for ZGA by activating hundreds of genes that are necessary for further development, and several endogenous retroelements^8–10,13^. Although the detailed function of most of DUX4 target genes remains elusive, it is thought that DUX4 induces several important factors that are involved in the creation of a permissive environment that allows transcription from the newly formed diploid genome.

Upon herpesviral infection the viral genome enters the nucleus and is packaged into chromatin structures, although the degree of chromatinization of the viral genome is discussed, in particular for HSV-1. However, it is widely accepted in the field that herpesvirus genomes are subjected to epigenetic silencing, and that they evolved strategies to prevent epigenetic silencing to allow transcription necessary for viral replication and virus transmission^46^. We show that herpesviruses from all human subfamilies, papillomaviruses as well as Merkel cell polyomavirus induce a robust expression of DUX4 upon infection in vitro and in vivo (Fig. 1A–E, Fig.4 A–F). This hints at a highly conserved mechanism in the coevolution of DNA viruses with their respective hosts. Herpesviruses and papilloma- / polyomaviruses belong to different viral realms, the highest taxonomic order of viruses, which indicates at a highly conserved mechanism. Even for herpesviruses this is highly remarkable since alpha-, beta-, and gamma-herpesviruses were split into three separate lineages about 100-200 million years ago^47^. For HSV-1, we demonstrate that DUX4 expression is induced by viral tegument/immediate-early proteins ICP0 and ICP4, indicating that this is an active induction by herpesvirus proteins (Fig. 2C, D). ICP4 directly binds to the DUX4 locus, however, for full DUX4 induction the E3-ubiquitin-ligase activity of ICP0 is needed (Fig. 2D) indicating that ICP0 degrades a cellular protein that mediates silencing of the DUX4 locus. ICP0 and ICP4 induce expression of DUX4 at early stages of infection for a brief period, indicated by 4sU-labelling of RNAs (Fig. 2B). Blocking viral DNA replication with PAA results in higher levels of DUX4 and its target genes (Fig. S2B), suggesting DUX4 behaves similarly to the strong HSV-1 beta genes, which are upregulated early in infection and are shut down during the switch to viral DNA replication and late gene expression. Interestingly, this very short induction of DUX4 resembles the mechanism of DUX4 induction during ZGA, where a short burst of DUX4 is sufficient for target gene activation and reprogramming of the embryo but prevents toxicity mediated by DUX4.

Moreover, we could demonstrate that herpesviral DUX4 induction is essential for efficient herpesviral gene expression and replication. Depletion of DUX4 from cells results in a strong reduction of most herpesviral gene and protein expression. In addition, DUX4 ko affects HSV-1 DNA replication (Fig. 5G). HSV-1 as well as HSV-2 show drastically reduced viral spread in the absence of DUX4. We observe about 100-fold reduction in virus progeny in ko cells compared to wt cells (Fig. 5D, E). Mechanistically, our data suggest that DUX4 promotes HSV replication independent of its known interaction partner p300/CBP, as treatment of cells with a p300/CBP inhibitor did not affect ICP0 and ICP27 expression (Fig. 5H). We hypothesize that herpesviruses evolved to partially mimic ZGA by actively inducing DUX4 expression. We found that 843 cellular genes are upregulated upon HSV-1 infection and ZGA (Fig. 3F, G). Upon HSV-1 infection, DUX4 binding correlates with an increase in open chromatin and a decrease in repressive chromatin (Fig. 3E), reflecting the role of DUX4 in ZGA. ZGA is conserved in all animals, and due to its significance in embryonic development there is very little room for the host to antagonize this viral mimicry of ZGA^44^. Any interference with DUX4 function, for example by mutations in the coding sequence or by preventing DUX4 expression could lead to drastic changes in the embryonic development that are incompatible with life. Thus, from a viral perspective, it is beneficial to exploit a host gene which is essential for host development for its own purpose in order to limit mutations that affect viral replication.

In addition to its role in ZGA and in the development of FSHD, DUX4 also plays an important role in a variety of human cancers^48^. Reanalysis of almost 10.000 cancer transcriptomes from The Cancer Genome Atlas (TCGA) showed DUX4 re-expression in many human cancers^48^. The authors speculate that expression of DUX4 and DUX4 target genes may contribute to tumorigenesis^48^. Interestingly, EBV, KSHV, HPV and MCPyV are known to cause cancer in humans^5^. We observe expression of DUX4 target genes in EBV-positive nasopharynx carcinoma cells, HPV-positive head and neck cancer and Merkel cell carcinoma, but not in healthy tissue from the same donors (Fig. 4B–F). This indicates that the observed DUX4 induction is also of relevance in vivo. It is worth speculating whether DUX4 expression induced by EBV, KSHV, HPV and MCPyV also contributes to viral oncogenesis. In addition, Chew et al. showed that DUX4 expression results in a downregulation of MHC-Class I expression by interfering with STAT1 signaling, hinting at an immune evasion mechanism^48,49^ that might also be important for viral infections.

Our data points at a very important if not essential role for DUX4 in herpesviral gene expression and replication. We could demonstrate that depletion of DUX4 in cells impairs viral replication. Viral immediate-early gene products accumulate in DUX4 ko cells whereas expression of early and late genes is reduced. This hints at a role of DUX4 in the switch between immediate-early and early late gene expression. It was thought that the herpesviral replication cascade is solely regulated by viral proteins, but our data show that DUX4 as a host factor is crucial for this regulation. As such, it is tempting to speculate that DUX4 and DUX4 downstream genes could be targeted for therapy of herpesvirus-associated diseases. Blocking DUX4 means preventing herpesviral gene expression and subsequent viral replication from the beginning. Targeting a cellular protein has the major advantage that viral escape mutants and resistance-formation are unlikely, but usually this comes with the risk of possible side-effects. However, DUX4 is not expressed in adult somatic tissue, one can expect that the side-effects are negligible. Using the mini-TRIMAway assay we could demonstrate that targeting DUX4 at the protein level hampers HSV-1 replication (Fig. 6D,E) and that DUX4 might serve as an attractive target for anti-herpesviral therapy in the future.

## Methods

### Ethics statement

Our research complies with all relevant ethical regulations. FFPE liver samples from patients with clinically and diagnostically proven HSV-1, hepatitis B (HBV), or hepatitis C virus (HCV)-induced hepatitis were obtained for diagnostic purposes and provided by the BioBanks of the Erasmus MC. According to the institutional “Opt-Out” system, which is defined by the National “Code of Good Conduct” [Dutch: Code Goed Gebruik, May 2011], these surplus human liver tissues were made available for the current study. Information about sex of anonymous study participants wasn’t available.

### Cell culture and viruses

Cells and cell lines were obtained from ATCC (293T (CRL-3216); HFF-1(SCRC-1041); Vero (CCL-81); Raji (CCL-86); NDF, primary melanocytes) and Horizon (HAP1 Parental Cell Line (catalog number C631)). iSLK cells were kindly provided by Don Ganem (San Francisco).

HEK 293T, primary HFF1 and Vero cells were maintained in Dulbecco’s modified Eagle’s medium (DMEM, Thermo Fisher Scientific) supplemented with 10% (vol/vol) heat-inactivated fetal bovine serum (FBS, Thermo Fisher Scientific), 2 mM GlutaMAX (Thermo Fisher Scientific), 1 mM HEPES (Thermo Fisher Scientific) and 1% (vol/vol) gentamycin or penicillin-streptomycin (vol/vol). HAP1 were cultured in Iscove’s Modified Dulbecco’s Medium (IMDM,Gibco) supplemented with 10% (vol/vol) fetal bovine serum (FBS, Anprotec) and 1% (vol/vol) penicillin-streptomycin (Sigma-Aldrich). iSLK harbouring recombinant (r) KSHV.219 were cultured in DMEM supplemented with 10% (vol/vol) FBS, 2 mM GlutaMAX (Thermo Fisher Scientific), 10 mM HEPES buffer (Thermo Fisher Scientific) and 1% (vol/vol) penicillin-streptomycin (Sigma Aldrich), 1 µg/ml Puromycin (Invitrogen) and 250 µg/ml G418 Genaxxon). EBV-positive Raji and EBV-negative Ramos cell lines derived from human Burkitt’s lymphoma were cultured in Roswell Park Memorial Institute medium (RPMI-1640, Gibco) supplemented with 10% FCS, 100 IU/ml penicillin, 100 µg/ml streptomycin and 2 mM glutamine. For the induction of EBV lytic cycle gene expression, 0.5 × 10^6^ cells/mL were treated with 25 ng/ml PMA and 1 µg/ml ionomycin at 37 °C and 5% CO_2_ for 12 h. All cells were kept under standard culture conditions and monthly checked for mycoplasma contamination (MycoAlert Kit, Lonza).

Lytic replication of KSHV in iSLK.219 was induced by adding 1 µg/ml doxycycline (Ratiopharm). Infection experiments were performed with HSV-1 strain KOS, HSV-1 GFP (F-strain, provided by B. Kaufer, Berlin), HSV-2-GFP (strain MS, kindly provided by Beate Sodeik, Hannover) or HCMV (strain Ad-169). HSV-1 deltaICP0 (ICP4-YFP) and HSV-1 delta ICP27 were provided by R. Everett (University of Glasgow). HSV-1 T-VEC del ICP34.5/ICP47 IMLYGIC®, Talimogene laherparepvec) was provided by L. Heinzerling (Friedrich-Alexander-Universität Erlangen-Nürnberg). HSV-1 expressing ICP4-YFP and VP26-RFP is a new recombinant virus generated in the background of strain 17 by recombining HSV-1 expressing ICP4-YFP (obtained from Matthew D. Weitzman) and HSV-1 expressing VP26-RFP (obtained from Oren Kobiler) and plaque purifying for 5 cycles. Pox virus infections were performed with VACV-WR, MVA-T7, MPXV-GA (Gabon-1987, Clade I) MPXV-IC (Ivory Coast-2012, Clade IIa), MPXV-FR (Freiburg-2022, Clade IIb), MPXV-RKI (RKI-2022, Clade IIb), CPXV (Hum/Gri 07/01) and CMLV (CP-1). Poxviruses were kindly provided by Georg Kochs, Freiburg.

### Reagents, plasmids and transfections

Transfections were performed with GenJet (SignaGen Laboratories) or Lipofectamin 2000 (Thermo Fisher Scientific) according to the manufacturer’s protocol. Plasmids are listed in Table 1.

**Table 1:** Reagents

Primary Antibodies and Plasmids			
Anti-DUX4 (E5-5)		Abcam	Ab124699
Anti-DUX4 (clone 9A12)		EMD Millipore	MABD116
Anti-DUX4 (P4H2)		Thermo Fisher Scientific	MA5-16147
VP16 (14-5)		Santa Cruz Biotechnology Inc	Sc-7546
HSV-1 ICP0 antibody (11060)		Santa Cruz Biotechnology Inc	Sc-53070
HSV-1/2 ICP27 antibody		Santa Cruz Biotechnology Inc	Sc-69806
HSV-1 gD antibody		Santa Cruz Biotechnology Inc	Sc-21719
HSV-1 ICP4 (H943) antibody		Santa Cruz Biotechnology Inc	Sc-69809
Anti-HA.11		Covance	MMS-101R
THE GFP antibody		Genescript	A01704-40
Anti-HCMV gB (27-287)		Hybridoma	
Anti-ZSCAN4		Sigma-Aldrich/Merck Millipore	HPA006491
THE β-Actin antibody, HRP		Genescript	A00730-40
GAPDH antibody, Biotin		Genescript	A00915
Β-Tubulin antibody		Genescript	A01717-40
THE α-Tubulin antibody		Genescript	A01410-40
Normal Rabbit IgG		Cell Signaling Technology	#2729
HSP 90 (4F10)		Santa Cruz Biotechnology Inc	Sc-69703
**Plasmids**			
LentiCRISPRv2	Addgene	52961	
pMD2.G	Addgene	12259	
psPAX2	Addgene	12260	
pCW57.1-DUX4-CA	Addgene	99281	
pCW57.1-Luciferase	Addgene	99283	
pCW57.1_blasti	Addgene	194067	
pCS2-mkgDUX4	Addgene	21156	
pTYB12	NEB	N6709S	

### Western Blotting

Cells were either directly lysed in 2x Laemmli-buffer or in RIPA HS buffer (10 mM Tris-HCl pH 8.0, 1 mM EDTA, 500 mM NaCl, 1% Triton X-100 (vol/vol), 0.1% SDS (vol/vol), 0.1% deoxycholic acid (DOC) with Aprotinin and Leupeptin, MG-132 and sodium metavanadate (Sigma-Aldrich)). Cell pellets were centrifuged at 4 °C, 18213 x g for 30 min in an Eppendorf centrifuge. Samples were diluted with Laemmli-SDS sample buffer and heated for 5 min at 95 °C. For DUX4 Western blots cells were treated 3 h prior harvesting with 10 µM MG-132. Antibodies used are listed in Table 1.

### qRT-PCR

RNA was extracted using the Direct-zol RNA Miniprep Plus kit from Zymo Research or the TRIzol reagent (Invitrogen Life Science) according to manufacturer’s instructions. Reverse transcription was performed using the Super Script IV Kit (Thermo Fisher Scientific) or using the LunaScript RT SuperMix kit (New England BioLabs) according to manufacturer’s instructions. qRT-PCR was carried out using TaqMan™ Universal PCR Master Mix I (Applied Biosystems, Thermo Fisher Scientific) 0.1 µg as template on a 7500 Fast Real-Time PCR machine or using the Luna Universal Probe qPCR Master Mix (New England Biolabs) for measurement on a C1000 Touch Termal Cycler (BioRad). In addition, reverse transcription and qRT-PCR were conducted in one step using the Luna Universal Probe One-Step RT-qPCR kit (New England Biolabs) following manufacturer’s protocols. Primers/probes are listed in Table 2. Expression levels for each gene were obtained by normalizing values to *HPRT1* or *VTRNA* and fold induction was calculated using the comparative CT method (ΔΔCT method).

**Table 2:** Oligonucleotides

Real time quantitative PCR probes			
DUX4L	5’-TCAGCCAGAATTTCACGGAAG-3’ 5’-CCATTCTTTCCTGGGCATCC-3’	FAM/ZEN/3IABκFQ	IDT
ZSCAN4	5’-CTAGTCACACATCAGCTCAGT-3’ 5’-TTCAGTCTCTTGCCTTGTGTC-3’	FAM/ZEN/3IABκFQ	IDT
TRIM49B	5’-GCTGCTGAGGAACACCA-3’ 5’-ATTGCTTCTAGCCTTAAATTCACAT-3’	FAM/ZEN/3IABκFQ	IDT
TRIM48	5’-ATCACTGGACTGAGGGACA-3’ 5’-GGGCGGATTTTGACGGT-3’	FAM/ZEN/3IABκFQ	IDT
ICP0	5’-CGGACACGGAACTGTTCGA-3’ 5’-CGCCCCCGCAACTGC-3’	FAM/ZEN/3IABκFQ	IDT
HPRT1	5’-CGAGATGTCATGAAGGAGATGG-3’ 5’-TCAGCAAAGAACTTATAGCCCC-3’	HEX/ZEN/3IABκFQ	IDT
VTRNA1	5’-TTTAATTGAAACAAGCAACCTGTCT-3’	FAM/NFQ	Thermo Fisher Scientific
TRIM43	5‘-TCATCTGATTCGACGGCAAG-3´ 5´-AGATGGTGACAGGGTCTACC-3´	FAM/ZEN/3IABκFQ	IDT
ICP27	5’-ACATCTTGCACCACGCCAG-3’ 5’-CGCCAAGAAAATTTCATCGAG-3’	FAM/ZEN/3IABκFQ	IDT
gG	5’-GCAGGCAYACGTAACGCACGCT-3’ 5’-TTCTCGTTCCTCACTGCCTCCC-3’	5Cy5/ 3IAbRQSp	IDT
BZLF1	5’-TTCAGAATCGCATTCCTCCAG-3’ 5’-CTAGCAGACATTGGTGTTCCA-3'	FAM/ZEN/3IABκFQ	IDT
BRLF1	5’-CAGGAGTTAGCCTCAGAAAGTC-3’ 5’-ATACCATACAGGACACAACACC-3'	FAM/ZEN/3IABκFQ	IDT
EBNA1	5’-CACATGTCGTCTTACACCATTGA-3’ 5’-TGTGGGCCGGGTCCA-3'	FAM/ZEN/3IABκFQ	IDT
**PCR primer for EMSA assay**			
HSV1-BS1	fwd: 5’-/5Cy5/ GTGTACCACTGCCACTGTCG-3’ rev: 5’-/5Cy5/ GTCTGATCATGCCCCATACC-3’		IDT
HSV1-BS2	fwd: 5’-/5Cy5/ ACATGACGTTCCACAGGTCC-3’ rev: 5’-/5Cy5/ TCACCGAGCCAGAAACTACG-3’		IDT
HSV1-No-BS	fwd: 5’-/5Cy5/ CGTGAACCAAAGACGAGGGC-3’ rev: 5’-/5Cy5/ CCACGTTGAGAAGCTCGTCG-3’		IDT
KSHV-BS1	fwd: 5’-/5Cy5/ ACGTACTAAATCTACTTGACAC-3’ rev: 5’-/5Cy5/CAGACACTATCCCAGAAAC-3’		IDT
KSHV-BS2	fwd: 5’-/5Cy5/CTCTGTAGAGGTGATGTCC -3’ rev: 5’-/5Cy5/ATTATTATGAGGCCTATCTG -3'		IDT
KSHV-BS3	fwd: 5’-/5Cy5/ATCTACATATAGCGTCTACACC-3’ rev: 5’-/5Cy5/AGGTTACATTAGTAGTGTCACG-3'		IDT
**PCR primer for HSV-1 Standard**			
HSV-1 gG	fwd: 5’-TTGGCACAAAAAGACCCCGA-3’ rev: 5’- AACTAGATACCACCGCCTTTATTG-3’		IDT
**PCR primer for nanobody cloning**			
Nanobody	fwd: 5’- AGAGCTCGTTTAGTGAACCGTC-3’ rev: 5’- GGTGGACCGGTTCATTACTAAAC-3’		IDT
Backbone	fwd: 5’- GTTTAGTAATGAACCGGTCCACC-3’ rev: 5’- GGCGATCTGACGGTTCACTAAAC-3’		IDT

For qRT-PCR on the viral DNA, the genomic DNA was extracted using the *Quick*-DNA MiniPrep kit (Zymo Research). qRT-PCR was conducted using the Luna Universal Probe qPCR Master Mix (New England Biolabs) and quantified with an HSV-1 standard.

### CRISPR and sgRNAs

All sgRNAs used in this study were previously described and are listed in Table 3. sgRNAs were cloned into LentiCRISPRv2 plasmid gifted from F. Zhang and plasmids verified by sequencing. Lentiviruses were packaged with pMD2.G and psPAX2 (both gifted from D. Trono) into HEK 293T cells. HEK 293T cells were seeded into 12-well plates and HAP1 cells into 6-well plates before lentiviral supernatants were added at 70–80% confluence. Plates with HEK 293T cells were centrifuged at 300 x g for 2 min. The medium was changed to normal culture medium the next day and selection with 1 µg/ml (HAP1) or 2 µg/ml (HEK293T) Puromycin in normal culture medium started on day 3.

**Table 3:** sgRNAs

Small guide RNAs for CRISPR/Cas		
DUX4 #1 (E1-3; v1)	5’-CACCGCACCCGGGCAAAAGCCGGG-3’ 5’-AAACCCCGGCTTTTGCCCGGGTGC-3’	IDT
DUX4 #2 (E1-4)	5’-CACCGCTGGAAGCACCCCTCAGCG-3’ 5’-AAACCGCTGAGGGGTGCTTCCAGC-3’	IDT
DUX4 #3 (p8)	5’-CACCGTCGGACAGCACCCTCCCCG-3’ 5’-AAACCGGGGAGGGTGCTGTCCGAC-3’	IDT

### DUX4 protein purification

The DUX4 protein was purified using the Intein Mediated Purification with an Affinity Chitin-binding Tag (IMPACT) system (New England Biolabs). The coding sequence of DUX4 was subcloned into the pTYB12 vector using EcoR1 and Sap1 restriction sites, with an intein-CBD tag added to the N-terminus of DUX4. Protein expression was induced by adding 0.4 mM of IPTG to ER2566 cells at an OD600 = 0.5 overnight at 18 °C. Bacterial pellets were then resuspended in lysis buffer (20 mM Na-HEPES, 500 mM NaCl, 1 mM EDTA, 0.1% Triton X-100 and protease inhibitors (cOmplete™ Proteaseinhibitor-Cocktail, Sigma Aldrich)) and lysed using a French Press. The lysates were centrifuged at 15.000 g at 4 °C for 30 min, and the clarified lysate was slowly loaded onto the chitin column for purification using the ӒKTA pure™ chromatography system. The beads were then washed with 50 bed volumes of Column Buffer (20 mM Na-HEPES, 500 mM NaCl, 1 mM EDTA, and 0.1% Triton X-100) before protein cleavage with washing buffer containing 50 mM DTT. Finally, the eluate was further purified with Size Exclusion Chromatography using a Superdex 200 Increase 10/300 GL column (Cytiva).

### Inhibition of p300/CBP

The inhibitor A-485 (MedChemExpress) was used to inhibit p300/CBP. HFF1 cells were seeded one day before infection. A485 was added in the concentrations of 2.6 nM and 9.8 nM 30 min prior to the infection (HSV-1 GFP, MOI 3) and the RNA was harvested 8 h post infection.

### Doxycycline inducible cell lines (DUX4 and nanobody)

For inducible DUX4 overexpression, the DUX4 and Luciferase constructs were cloned into the pCW57.1_blasti vector using restriction cloning with AgeI and XhoI enzymes. Lentiviruses were packaged with pMD2.G and psPAX2 into HEK 293T. Raji, 293T and HFFtert cells were transduced with lentiviruses and selected with 10 µg/ml Blasticidin (Invitrogen). DUX4 expression was induced by adding 1 µg/ml Doxycycline to the medium.

The custom nanobodies targeting DUX4 were purchased from Gulliver Biomed (Ghent, Belgium). The coding sequence of the nanobodies linked with the TRIM21 ring was synthesized by Integrated DNA Technologies (IDT) and amplified by the primer forward: agagctcgtttagtgaaccgtc and reverse: ggtggaccggttcattactaaac. The sequence of the backbone pCW57.1_blasti plasmid was amplified by the primer forward: gtttagtaatgaaccggtccacc and reverse: ggcgatctgacggttcactaaac. The sequence information is provided in the Supplementary Data 9. Both fragments then linked by Gibson Assembly® Master Mix (New England Biolabs) following the manufacturer’s protocol.

Nanobody inducible 293T cell lines cells were generated by lentiviral transduction. To select for successfully transduced 293T, the cells were then provided with medium containing 10 µg/ml Blasticidin.

### Chip-Seq

For CHIPmentation primary HFF cells were seeded in T175 flasks and infected with HSV-1 KOS (MOI of 10) for different time points. CHIPmentation was conducted as published elsewhere^50^. Cells were sonicated using the Bioruptor (Diagenode) for 30 cycles. For the immunoprecipitation protein G Dynabeads (Thermo Fisher Scientific) were used. Samples were incubated with either 2.5 µg anti-DUX4 (E5-5) (Abcam) or Normal Rabbit IgG (Cell Signaling Technology) as control. Samples were purified using AMpureXP beads (Beckman Coulter) according to manufacturer’s description. Libraries were sequenced on HiSeq 4000 System (Illumina).

### CUT&Tag

Primary HFF cells were infected with HSV-1 GFP (MOI 1) in PBS supplemented with 0.1% Glucose and 1% FCS. The remaining virus was washed away with a low pH buffer (40 mM Citric acid, 10 mM KCl, 135 mM NaCl, pH3) after 1 h at 37 °C. CUT&Tag was performed according to the manufacturing protocol (CUT&Tag-IT Assay Kit, Active Motif) using 2,5 µg anti-Dux4 E5-5 (Abcam) or 2,5 µg rabbit IgG (Cell Signaling Technology). In short, cells were bound to concanavalin A-coated magnetic beads and permeabilized for subsequent incubation with primary and secondary antibodies. Specific cutting and addition of adapters was mediated by a protein A-Tn5 transposase fusion protein. Libraries were sequenced on HiSeq 4000 System (Illumina).

### CUT&RUN

Primary HFF cells were infected with HSV-1 GFP (MOI of 1) in PBS supplemented with 0.1% Glucose and 1% FCS. The remaining virus was washed away with a low pH buffer (40 mM Citric acid, 10 mM KCl, 135 mM NaCl, pH3) after 1 h at 37 °C. CUT&RUN was performed using the option 1 of the CUT&RUN protocol (v3, 10.17504/protocols.io.zcpf2vn). 1 µg anti-DUX4 (E5-5 Abcam), 1 µg anti-H3K27me3 (Active Motif) or 1 µg rabbit IgG (Cell Signaling Technology) were used accordingly and 5 pg Drosophila Spike in DNA was added to each sample for normalization. Libraries were generated using the NEBNext Ultra II DNA Library Prep Kit.

### Next generation RNA-seq

HEK 293T wildtype cells and HEK 293T CRISPR/Cas knockout cells were seeded in T25 flasks. Cells were infected for different time points with HSV-1 KOS (MOI 10). HAP1 wildtype cells and HAP1 DUX4 ko cells were seeded in 6-well plates and infected with HSV-1 GFP (MOI 1) in PBS supplemented with 0.1% Glucose and 1% FCS. After 1 h at 37 °C the remaining virus was washed away with a low pH buffer (40 mM Citric acid, 10 mM KCl, 135 mM NaCl, pH3). For pox virus infection cells were seeded in 6-well plates the day before infection. An aliquot of the cell supernatant was removed and kept at 37 °C. Cells were infected at an MOI 1 and after 2 h incubation at 37 °C the virus-containing medium was changed to conditioned medium. Cells were lysed in TRIzol (Life Technologies by Thermo Fisher Scientific), and total RNA was isolated using the RNA clean and concentrator kit (Zymo Research), according to the manufacturer’s instructions. Sequencing libraries were prepared using the NEBNext Ultra II Directional RNA Library Prep Kit for Illumina (NEB) with 9 cycles PCR amplification, and sequenced on a HiSeq 4000 device with 1×50 cycles. For quantification of viral gene expression alignments were done using hisat2^49^ on the HSV-1 genome (strain 17, GenBank accession no. NC_001806) and read counts per gene quantified using quasR^51^.

### EMSA

Viral DNA of HSV-1 GFP (F-strain), HSV-1 KOS and KSHV (BAC16) was isolated using the *Quick*-DNA MiniPrep kit (Zymo Research). About 600 bp long fragments containing either the DUX4 binding motif or no DUX4 binding motif were amplified from the viral genomes using the primers depicted in Table 2.The EMSA was performed as described by Lee et al. ^51^ using 50 ng of amplified viral DNA, which was incubated 30 min at room temperature with 1 µg purified DUX4 protein and 2 µL salmon sperm DNA (D7656, Sigma-Aldrich).

### Flow cytometry

HAP1 wt and ko cells were simultaneously seeded and infected with HSV-1 GFP or HSV-2 GFP at a MOI of 0.1. The days 1, 2, 3 and 4 post infection were analyzed by flow cytometry.

To check the replication of HSV-1 upon nanobodies expression, the nanobody induction in the corresponding cell lines was initiated by the addition of 2 µg/ml doxycycline and infected with HSV-1 GFP at a MOI of 0.05.

For flow cytometry cells were detached by scraping, fixed in 1% Paraformaldehyde (PFA) for 20 min and resuspended in FACS buffer (PBS supplemented with 2% FCS and 0.5 mM EDTA). GFP expression was measured with BD LSRFortessa and the data was analyzed with FlowJo.

### The prediction of DUX4 nanobody interaction by AlphaFold-Multimer

The interaction of DUX4 and nanobody were predicted in google colab by using AlphaFold-Multimer. The models were ranked by the ranking_confidence score and rank 1 was selected.

### Predicted DUX4 binding sites in different herpesviral genomes

The following viral genomes were downloaded at NCBI: HSV-1 (GU734771.1, KT899744.1), HSV-2 (Z86099.2), VZV (NC_001348.1), EBV (NC_007605), HCMV (MN900952.1), HHV6A (NC_001664.4), HHV7 (U43400.1) and KSHV (OK358814). The viral genomes were analysed for DUX4 binding sites with FIMO^52^ using the DUX4 binding motif obtained from the CUT&Tag experiment with a cutoff for the *p*-value of 0.001.

### H&E and Immunohistochemistry

Four-micrometer thick sections of formalin-fixed paraffin-embedded (FFPE) tissue samples were stained with haematoxylin and eosin according to the manufacturer’s instructions using the HE 600 automatic staining system (Ventana Medical Systems Inc). Immunohistochemistry stainings were performed with an automated, validated and accredited staining system (Ventana Benchmark ULTRA, Ventana Medical Systems) using optiview universal DAB detection kit. In brief, following deparaffinization and heat-induced antigen retrieval with CC1 (#950-500, Ventana) for 32 min the samples were incubated with anti-DUX4 monoclonal antibody (P4H2, Thermo Fisher Scientific, 1:800) for 32 min at 37 °C. Incubation was followed by optiview detection, hematoxylin II counterstain for 8 min, and a blue coloring reagent for 8 min according to the manufacturer’s instructions (Ventana Medical Systems Inc). Slides were scanned using the Nanozoomer HT2.0 (Hamamatsu, Japan).

### ChIP-seq

ChIP-seq data processing was done using the PiGx-ChIP-seq pipeline (10.1093/gigascience/giy123). In short, adapters and low quality bases were trimmed from reads using Trim-galore. The reads were mapped on the hg19 version of the human genome, combined with HSV-1 genome, using Bowtie2 with *k* = 1 parameter. bigWig tracks were created by extending reads to 200, collapsing them into pileups, and normalizing to reads per million. Peak calling was done with MACS2 (https://github.com/taoliu/MACS) using the default parameters. Motif discovery was done using MEME^53^ with the default parameters, on the top 100 peaks (sorted by q value), in a region of +/- 50 bp around the peak center. Peak annotation was done using the hg19 ENSEMBL GTF file, downloaded on 17.03.2017. from the ENSEMBL database^54^. Peaks were annotated based on the following hierarchy of functional categories: tss -> exon -> intron -> intergenic (eg. if a peak overlapped multiple categories, it was annotated by the class that is highest in the hierarchy). Peaks were overlapped with the hg19 Repeatmasker repeat annotation, downloaded from the UCSC database on 03.02.2015.

### CUT&Tag

For CUT&Tag analysis, the data was mapped to the hg19 version of the human genome with the viral genome using Bowtie2 with *k* = 1 parameter. bigWig tracks were created by extending reads to 200, collapsing them into pileups, and normalizing to reads per million. Bam files were uploaded to the Galaxy web platform (10.1093/nar/gkae410). Peak calling was executed by MACS2 using the default parameters. Motif discovery was done with MEME^53^ using the default parameters on all peaks of the human genome in a region +/-50 bp around the peak center.

### CUT&RUN

The data for CUT&RUN analysis was uploaded to the Galaxy web platform 10.1093/nar/gkae410). As the first step data was aligned to the human genome (CMH13 2.0) and the HSV-1 genome (GU734771.1 or KT899744.1) using bowtie2. BamCoverage was used to normalize the data to the Drosophila Spike in DNA (Scaling factor = %dm3 mapped reads/ %human mapped reads) and generate bigwig files. Peak calling was executed by MACS2 using the default parameters.

### Bulk RNA-seq

The bulk RNA-seq. raw data of Human Adenovirus 5 infected cells (PRJEB57806), FSHD patients (GSE153301), and 8-cell-stage embryo cells (GSE36552), as well as the single-cell RNA-seq. data of nasopharyngeal carcinoma patients (GSE162025), were downloaded from the NCBI database. The bulk RNA-seq. data were aligned to the human genome (hg38) using STAR and then normalized using DESeq2. The R package VennDiagram was used to generate a Venn diagram showing the overlapping genes between the different samples. To visualize the DUX4 target genes expression patterns, the R package pheatmap was used to create a heatmap. For the single-cell RNA-seq. data analysis of nasopharyngeal carcinoma patients, the STARsolo pipeline was used to align sequencing reads to the human reference genome hg38 and to generate feature-barcode matrices. The gene expression matrices for all PBMC and tumour cells were combined and converted to a Seurat object using the R package Seurat. The gene expression matrices were then log-normalized and linearly regressed using the NormalizeData and ScaleData function of the Seurat package. Finally, the scVirusScan pipeline was employed to identify viruses present in all PBMC and tumour cells.

Viral abundances in ICGC database were taken from a previous paper^55^. The processed RNA-seq. data from ICGC tumors was downloaded from the PCAWG database. DUX4 score was obtained by applying the decoupleR method [10.1093/bioadv/vbac016] on the ICGC RNA-seq. data, using the top 50 DUX4 target genes. The final visualization was created by plotting the mlm score of DUX4 target genes versus the measured viral abundances.

For analysis of ATAC-seq and histone modification peaks at DUX4 binding sites we reanalyzed data from Hennig et al. ^33^, Gao et al. ^34^ and Geng et al. ^32^ using deeptools^56^.

### Comparison of HSV-1 gene expression with the embryonic expression profile

Expression profiles for HSV-1 and DUX4 ectopically expressed, 293T cells were taken from the following publication^6^. The embryonic expression profiles were downloaded from the ARCHS4 database. The data originated from the following repository GSE44183. Data was visualized using the ComplexHeatmap function.

### Comparison of repeat expression during HSV-1 infection and DUX4 overexpression

Expression profiles obtained from HSV-1 and DUX4 ectopically expressed, 293T cells were taken from the following publication^6^. DUX4 binding data extracted from the supplementary data from the following publication^14^. RNA - seq data was mapped using STAR, and the repeats were quantified by counting the number of, uniquely mapping, spliced reads overlapping with each transposon category. Expression was visualized using ComplexHeatmap. Prior to the visualization the reads were normalized to uninfected samples.

### Statistical analysis

*P*-values were calculated using an unpaired Student’s test. *P* < 0.05 was considered statistically significant. For the calculation of differentially expressed genes we used the DESeq2 package with default settings. First, for normalization, DESeq2 calculates size factors to normalize for differences in sequencing depth. Second, for dispersion estimation a negative binomial distribution is used to model overdispersion, and dispersions are estimated using shrinkage. Third, for differential expression testing, a Wald test is used to test for significant differences in expression (log2FC) between conditions. Fourth, for multiple testing correction, *P*-values are adjusted using the Benjamini-Hochberg procedure to control the false discovery rate (FDR). Genes with significant changes in expression (based on padj < 0.05) are considered to be differentially expressed .

**Table 4:** GEO accession numbers of sequencing data

Datasets (Generated in this study)	GEO accession number
RNA-seq. of Pox virus infection	GSE234489
CUT&RUN of HSV-1 infection	GSE272015
RNA-seq. of DUX4-KO, CUT&Tag of HSV-1 infection	GSE234489
CHIP-seq of DUX4 and RNAseq upon HSV-1 infection	GSE174759
**Datasets (Used for reanalysis)**	**Accession number**
ATAC-seq upon HSV-1 infection	GSE100611
CHIP-seq of histone marker upon HSV-1 infection	GSE124803
Pro-seq of HSV-1 infection	GSE106126
RNA-seq. of HSV1 infection	GSE59717
	GSE129715
	GSE97009
	GSE101435
RNA-seq. of FSHD	GSE153301
RNA-seq. of 8 cell stage	GSE36552
RNA-seq. of PAA treatment upon HSV-1 infection	GSM5608614
RNA-seq. of EBV infection	GSE155345
RNA-seq. of KSHV infection	GSE123898
	GSE179742
CHIP-seq of DUX4 (DUX4 induction)	GSM837613
ICP4 ChIP-seq	SRP131749
single-cell RNA-seq. of nasopharyngeal carcinoma	GSE162025
single-cell RNA-seq. of Head and neck squamous cell carcinoma	GSE164690
single-cell RNA-seq. of Merkel cell carcinoma tumor	GSE226438
RNA-seq. of Ad5 infection	PRJEB57806

### Reporting summary

Further information on research design is available in the Nature Portfolio Reporting Summary linked to this article.

## Supplementary information

Supplementary Information

Peer Review file

Description of Additional Supplementary Files

Supplementary Data 1

Supplementary Data 2

Supplementary Data 3

Supplementary Data 4

Supplementary Data 5

Supplementary Data 6

Supplementary Data 7

Supplementary Data 8

Supplementary Data 9

Reporting Summary

## Source data

Source Data

## Data Availability

The Sequencing data (RNA-seq., ChIP-seq, CUT&Tag and CUT&RUN) generated in this study have been deposited in the GEO database under accession codes listed in Table 4. In this table the published datasets used for reanalysis are shown as well. Source Data are provided as a Source Data file. Source data are provided with this paper.

## Code availability

Source code is available at github.com/jiangtan01/Herpesviruses-mimic-zygotic-genome-activation.

## References

[1] Pebody, R. G. et al. The seroepidemiology of herpes simplex virus type 1 and 2 in Europe. *Sex. Transm. Infect.* **80**, 185–191 (2004). 15170000 10.1136/sti.2003.005850 PMC1744847

[2] Zuhair, M. et al. Estimation of the worldwide seroprevalence of cytomegalovirus: a systematic review and meta-analysis. *Rev. Med Virol.* **29**, e2034 (2019). 30706584 10.1002/rmv.2034

[3] Corey, L. & Wald, A. Maternal and neonatal herpes simplex virus infections. *N. Engl. J. Med* **361**, 1376–1385 (2009). 19797284 10.1056/NEJMra0807633 PMC2780322

[4] Stern, L. et al. Human cytomegalovirus latency and reactivation in allogeneic hematopoietic stem cell transplant recipients. *Front Microbiol.* **10**, 1186 (2019). 31191499 10.3389/fmicb.2019.01186 PMC6546901

[5] Plummer, M. et al. Global burden of cancers attributable to infections in 2012: a synthetic analysis. *Lancet Glob. Health* **4**, e609–616, (2016). 27470177 10.1016/S2214-109X(16)30143-7

[6] Full, F. et al. Centrosomal protein TRIM43 restricts herpesvirus infection by regulating nuclear lamina integrity. *Nat. Microbiol.* **4**, 164–176 (2019). 30420784 10.1038/s41564-018-0285-5 PMC6294671

[7] Friedel, C. C. et al. Dissecting herpes simplex virus 1-induced host shutoff at the RNA level. *J. Virol.* **95**, e01399–20 (2021). 10.1128/JVI.01399-20 PMC7925104 33148793

[8] De Iaco, A. et al. DUX-family transcription factors regulate zygotic genome activation in placental mammals. *Nat. Genet.* **49**, 941–945 (2017). 28459456 10.1038/ng.3858 PMC5446900

[9] Whiddon, J. L., Langford, A. T., Wong, C. J., Zhong, J. W. & Tapscott, S. J. Conservation and innovation in the DUX4-family gene network. *Nat. Genet.* **49**, 935–940 (2017). 28459454 10.1038/ng.3846 PMC5446306

[10] Hendrickson, P. G. et al. Conserved roles of mouse DUX and human DUX4 in activating cleavage-stage genes and MERVL/HERVL retrotransposons. *Nat. Genet.* **49**, 925–934 (2017). 28459457 10.1038/ng.3844 PMC5703070

[11] Lee, M. T., Bonneau, A. R. & Giraldez, A. J. Zygotic genome activation during the maternal-to-zygotic transition. *Annu Rev. Cell Dev. Biol.* **30**, 581–613 (2014). 25150012 10.1146/annurev-cellbio-100913-013027 PMC4303375

[12] Eckersley-Maslin, M. A., Alda-Catalinas, C. & Reik, W. Dynamics of the epigenetic landscape during the maternal-to-zygotic transition. *Nat. Rev. Mol. Cell Biol.* **19**, 436–450 (2018). 29686419 10.1038/s41580-018-0008-z

[13] De Iaco, A., Verp, S., Offner, S., Grun, D. & Trono, D. DUX is a non-essential synchronizer of zygotic genome activation. *Development* **147**, dev177725 (2020). 10.1242/dev.177725 PMC7099940 31806660

[14] Young, J. M. et al. DUX4 binding to retroelements creates promoters that are active in FSHD muscle and testis. *PLoS Genet.* **9**, e1003947 (2013). 24278031 10.1371/journal.pgen.1003947 PMC3836709

[15] Wang, J. et al. Primate-specific endogenous retrovirus-driven transcription defines naive-like stem cells. *Nature* **516**, 405–409 (2014). 25317556 10.1038/nature13804

[16] Snider, L. et al. Facioscapulohumeral dystrophy: incomplete suppression of a retrotransposed gene. *PLoS Genet.* **6**, e1001181 (2010). 21060811 10.1371/journal.pgen.1001181 PMC2965761

[17] Lemmers, R. J. et al. A unifying genetic model for facioscapulohumeral muscular dystrophy. *Science* **329**, 1650–1653 (2010). 20724583 10.1126/science.1189044 PMC4677822

[18] Himeda, C. L. et al. Myogenic enhancers regulate expression of the facioscapulohumeral muscular dystrophy-associated DUX4 gene. *Mol. Cell Biol.* **34**, 1942–1955 (2014). 24636994 10.1128/MCB.00149-14 PMC4019064

[19] Daxinger, L., Tapscott, S. J. & van der Maarel, S. M. Genetic and epigenetic contributors to FSHD. *Curr. Opin. Genet Dev.* **33**, 56–61 (2015). 26356006 10.1016/j.gde.2015.08.007 PMC4674299

[20] Hewitt, J. E. Loss of epigenetic silencing of the DUX4 transcription factor gene in facioscapulohumeral muscular dystrophy. *Hum. Mol. Genet* **24**, R17–23, (2015). 26113644 10.1093/hmg/ddv237

[21] Bakker, E. et al. The FSHD-linked locus D4F104S1 (p13E-11) on 4q35 has a homologue on 10qter. *Muscle Nerve Suppl.* **2**, S39–S44 (1995). 7739624

[22] Gabriels, J. et al. Nucleotide sequence of the partially deleted D4Z4 locus in a patient with FSHD identifies a putative gene within each 3.3 kb element. *Gene* **236**, 25–32 (1999). 10433963 10.1016/s0378-1119(99)00267-x

[23] Wyler, E. et al. Widespread activation of antisense transcription of the host genome during herpes simplex virus 1 infection. *Genome Biol.* **18**, 209 (2017). 29089033 10.1186/s13059-017-1329-5 PMC5663069

[24] Rutkowski, A. J. et al. Widespread disruption of host transcription termination in HSV-1 infection. *Nat. Commun.* **6**, 7126 (2015). 25989971 10.1038/ncomms8126 PMC4441252

[25] Birkenheuer, C. H., Danko, C. G. & Baines, J. D. Herpes simplex virus 1 dramatically alters loading and positioning of RNA polymerase II on host genes early in infection. *J. Virol.* **92**, e02184–17. (2018). 10.1128/JVI.02184-17 PMC5874419 29437966

[26] Fox, H. L., Dembowski, J. A. & DeLuca, N. A. A herpesviral immediate early protein promotes transcription elongation of viral transcripts. *mBio* **8**, e00745–17 (2017). 10.1128/mBio.00745-17 PMC5472187 28611249

[27] Shadle, S. C. et al. DUX4-induced bidirectional HSATII satellite repeat transcripts form intranuclear double-stranded RNA foci in human cell models of FSHD. *Hum. Mol. Genet* **28**, 3997–4011 (2019). 31630170 10.1093/hmg/ddz242 PMC7342170

[28] Nogalski, M. T. et al. A tumor-specific endogenous repetitive element is induced by herpesviruses. *Nat. Commun.* **10**, 90 (2019). 30626867 10.1038/s41467-018-07944-x PMC6327058

[29] Yao, Z. et al. DUX4-induced gene expression is the major molecular signature in FSHD skeletal muscle. *Hum. Mol. Genet* **23**, 5342–5352 (2014). 24861551 10.1093/hmg/ddu251 PMC4168822

[30] Dixon, R. A. & Schaffer, P. A. Fine-structure mapping and functional analysis of temperature-sensitive mutants in the gene encoding the herpes simplex virus type 1 immediate early protein VP175. *J. Virol.* **36**, 189–203 (1980). 6255206 10.1128/jvi.36.1.189-203.1980 PMC353630

[31] Everett, R. D. Trans activation of transcription by herpes virus products: requirement for two HSV-1 immediate-early polypeptides for maximum activity. *EMBO J.* **3**, 3135–3141 (1984). 6098466 10.1002/j.1460-2075.1984.tb02270.x PMC557829

[32] Geng, L. N. et al. DUX4 activates germline genes, retroelements, and immune mediators: implications for facioscapulohumeral dystrophy. *Dev. Cell* **22**, 38–51 (2012). 22209328 10.1016/j.devcel.2011.11.013 PMC3264808

[33] Hennig, T. et al. HSV-1-induced disruption of transcription termination resembles a cellular stress response but selectively increases chromatin accessibility downstream of genes. *PLoS Pathog.* **14**, e1006954 (2018). 29579120 10.1371/journal.ppat.1006954 PMC5886697

[34] Gao, C. et al. The epigenetic landscapes of histone modifications on HSV-1 genome in human THP-1 cells. *Antivir. Res.* **176**, 104730 (2020). 32014498 10.1016/j.antiviral.2020.104730

[35] Yan, L. et al. Single-cell RNA-seq. profiling of human preimplantation embryos and embryonic stem cells. *Nat. Struct. Mol. Biol.* **20**, 1131–1139 (2013). 23934149 10.1038/nsmb.2660

[36] Xue, Z. et al. Genetic programs in human and mouse early embryos revealed by single-cell RNA sequencing. *Nature* **500**, 593–597 (2013). 23892778 10.1038/nature12364 PMC4950944

[37] Liu, Y. et al. Tumour heterogeneity and intercellular networks of nasopharyngeal carcinoma at single cell resolution. *Nat. Commun.* **12**, 741 (2021). 33531485 10.1038/s41467-021-21043-4 PMC7854640

[38] Kurten, C. H. L. et al. Investigating immune and non-immune cell interactions in head and neck tumors by single-cell RNA sequencing. *Nat. Commun.* **12**, 7338 (2021). 34921143 10.1038/s41467-021-27619-4 PMC8683505

[39] Das, B. K. et al. Single-cell dissection of Merkel cell carcinoma heterogeneity unveils transcriptomic plasticity and therapeutic vulnerabilities. *Cell Rep. Med*. **4**, 101101 (2023). 37421947 10.1016/j.xcrm.2023.101101 PMC10394170

[40] Mariot, V. & Dumonceaux, J. Gene editing to tackle facioscapulohumeral muscular dystrophy. *Front Genome Ed.* **4**, 937879 (2022). 35910413 10.3389/fgeed.2022.937879 PMC9334676

[41] Choi, S. H. et al. DUX4 recruits p300/CBP through its C-terminus and induces global H3K27 acetylation changes. *Nucleic Acids Res.* **44**, 5161–5173 (2016). 26951377 10.1093/nar/gkw141 PMC4914088

[42] Clift, D. et al. A method for the acute and rapid degradation of endogenous proteins. *Cell* **171**, 1692–1706 e1618 (2017). 29153837 10.1016/j.cell.2017.10.033 PMC5733393

[43] Clift, D., So, C., McEwan, W. A., James, L. C. & Schuh, M. Acute and rapid degradation of endogenous proteins by trim-away. *Nat. Protoc.* **13**, 2149–2175 (2018). 30250286 10.1038/s41596-018-0028-3

[44] Schulz, K. N. & Harrison, M. M. Mechanisms regulating zygotic genome activation. *Nat. Rev. Genet.* **20**, 221–234 (2019). 30573849 10.1038/s41576-018-0087-x PMC6558659

[45] Messerschmidt, D. M., Knowles, B. B. & Solter, D. DNA methylation dynamics during epigenetic reprogramming in the germline and preimplantation embryos. *Genes Dev.* **28**, 812–828 (2014). 24736841 10.1101/gad.234294.113 PMC4003274

[46] Knipe, D. M. et al. Snapshots: chromatin control of viral infection. *Virology* **435**, 141–156 (2013). 23217624 10.1016/j.virol.2012.09.023 PMC3531885

[47] McGeoch, D. J., Cook, S., Dolan, A., Jamieson, F. E. & Telford, E. A. Molecular phylogeny and evolutionary timescale for the family of mammalian herpesviruses. *J. Mol. Biol.* **247**, 443–458 (1995). 7714900 10.1006/jmbi.1995.0152

[48] Chew, G. L. et al. DUX4 suppresses MHC class I to promote cancer immune evasion and resistance to checkpoint blockade. *Dev. Cell* **50**, 658–671 e657 (2019). 31327741 10.1016/j.devcel.2019.06.011 PMC6736738

[49] Spens, A. E., Sutliff, N. A., Bennett, S. R., Campbell, A. E. & Tapscott, S. J. Human DUX4 and mouse Dux interact with STAT1 and broadly inhibit interferon-stimulated gene induction. *Elife* **12**, e82057 (2023). 10.7554/eLife.82057 PMC10195082 37092726

[50] Schmidl, C., Rendeiro, A. F., Sheffield, N. C. & Bock, C. ChIPmentation: fast, robust, low-input ChIP-seq for histones and transcription factors. *Nat. Methods* **12**, 963–965 (2015). 26280331 10.1038/nmeth.3542 PMC4589892

[51] Lee, J. K. et al. Crystal structure of the double homeodomain of DUX4 in complex with DNA. *Cell Rep.* **25**, 2955–2962 e2953 (2018). 30540931 10.1016/j.celrep.2018.11.060 PMC6463520

[52] Grant, C. E., Bailey, T. L. & Noble, W. S. FIMO: scanning for occurrences of a given motif. *Bioinformatics* **27**, 1017–1018 (2011). 21330290 10.1093/bioinformatics/btr064 PMC3065696

[53] Bailey, T. L., Johnson, J., Grant, C. E. & Noble, W. S. The MEME suite. *Nucleic Acids Res*. **43**, W39–49, (2015). 25953851 10.1093/nar/gkv416 PMC4489269

[54] Zerbino, D. R. et al. Ensembl 2018. *Nucleic Acids Res.* **46**, D754–D761 (2018). 29155950 10.1093/nar/gkx1098 PMC5753206

[55] Zapatka, M. et al. The landscape of viral associations in human cancers. *Nat. Genet.* **52**, 320–330 (2020). 32025001 10.1038/s41588-019-0558-9 PMC8076016

[56] Ramirez, F. et al. deepTools2: a next generation web server for deep-sequencing data analysis. *Nucleic Acids Res*. **44**, W160–165 (2016). 27079975 10.1093/nar/gkw257 PMC4987876

