# Publisher Correction: Herpesviruses mimic zygotic genome activation to promote viral replication

**DOI:** 10.1038/s41467-025-57313-8

**Published:** 2025-03-05

**Authors:** Eva Neugebauer, Stephanie Walter, Jiang Tan, Nir Drayman, Vedran Franke, Michiel van Gent, Sandra Pennisi, Pia Veratti, Karla S. Stein, Isabelle Welker, Savaş Tay, Georges M. G. M. Verjans, H. T. Marc Timmers, Altuna Akalin, Markus Landthaler, Armin Ensser, Emanuel Wyler, Florian Full

**Affiliations:** 1 Institute of Virology, University Medical Center, and Faculty of Medicine, Albert-Ludwig-University Freiburg, Freiburg, Germany; 2 Spemann Graduate School of Biology and Medicine (SGBM), University of Freiburg, Freiburg, Germany; 3 Faculty of Biology, University of Freiburg, Freiburg, Germany; 4 Institute for Clinical and Molecular Virology, University Hospital Erlangen, Friedrich-Alexander-Universität Erlangen-Nürnberg, 91054 Erlangen, Germany; 5 The Department of Molecular Biology and Biochemistry, the Center for Virus Research and the Center for Complex Biological Systems, The University of California, Irvine, Irvine, CA 92697 USA; 6 Berlin Institute for Medical Systems Biology, Max Delbrück Center for Molecular Medicine, Helmholtz Society, Berlin, Germany; 7 HerpesLabNL, Department of Viroscience, Erasmus Medical Center, Rotterdam, The Netherlands; 8 The Pritzker School for Molecular Engineering, The University of Chicago, Chicago, IL 60637 USA; 9 German Cancer Consortium (DKTK), partner site Freiburg, a partnership between the DKFZ and Medical Center-University of Freiburg, and Department of Urology, Medical Center-University of Freiburg, Freiburg, Germany; 10 German Consulting Laboratory for HSV and VZV, Medical Center - University of Freiburg, Freiburg, Germany

**Keywords:** Herpes virus, Epigenetics

Correction to: *Nature Communications* 10.1038/s41467-025-55928-5, published online 16 January 2025

In this article the bands from the western blots in Fig. 5a were missing. The original article has been corrected.

