# Supplementary material for: Herpesviruses mimic zygotic genome activation to promote viral replication
Source: Nat Commun. 2025 Jan 16;16:710. doi: 10.1038/s41467-025-55928-5 (PMC11735616; doi:10.1038/s41467-025-55928-5)
Supplement: Supplementary file 1 — Supplementary Information [file 41467_2025_55928_MOESM1_ESM.pdf]

Supplemental Figure 1

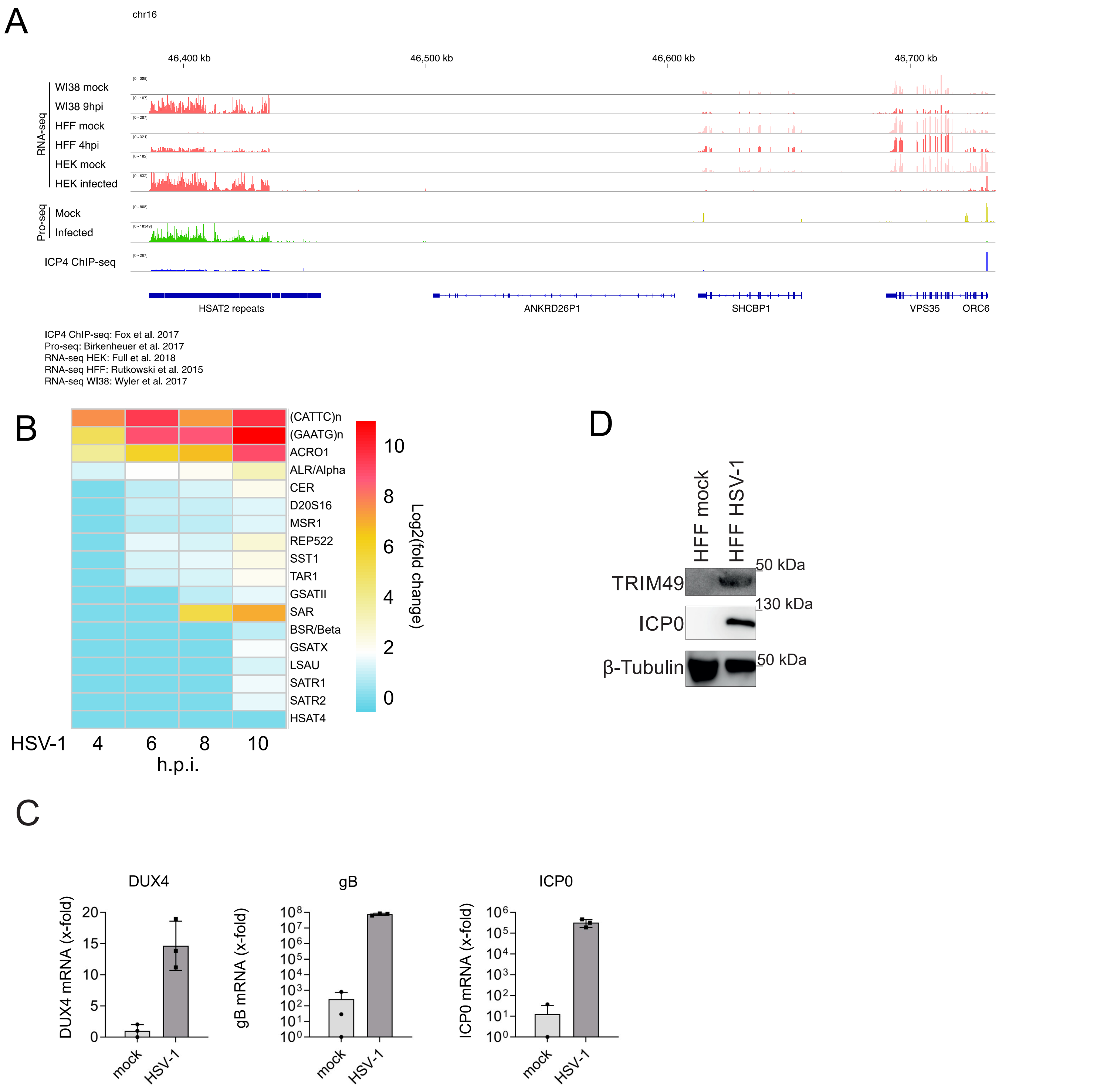

Fig. S1. A RNA-Seq. data of the HSATII repeats and neighbouring loci in WI38 9 hpi, HFF 4 hpi and HEK 293T cells 18 hpi. Pro-Seq. (Precision Run-On Sequencing) shows RNA-pol II occupancy at HSATII repeats. **B** The heatmap shows the expression of various satellite repeat loci. **C** qRT-PCR of primary melanocytes infected with HSV-1 for 18 h (MOI 0.1). HSV-1 ICP0 and gB served as infection controls. Data represent mean and s.d. of *n* = 3 (biological replicates). **D** Western blot of HFF cells infected with HSV-1 for 18 h with MOI 5 and analyzed for expression of TRIM49. ICP0 served as a control for viral infection. One representative out of n=4.

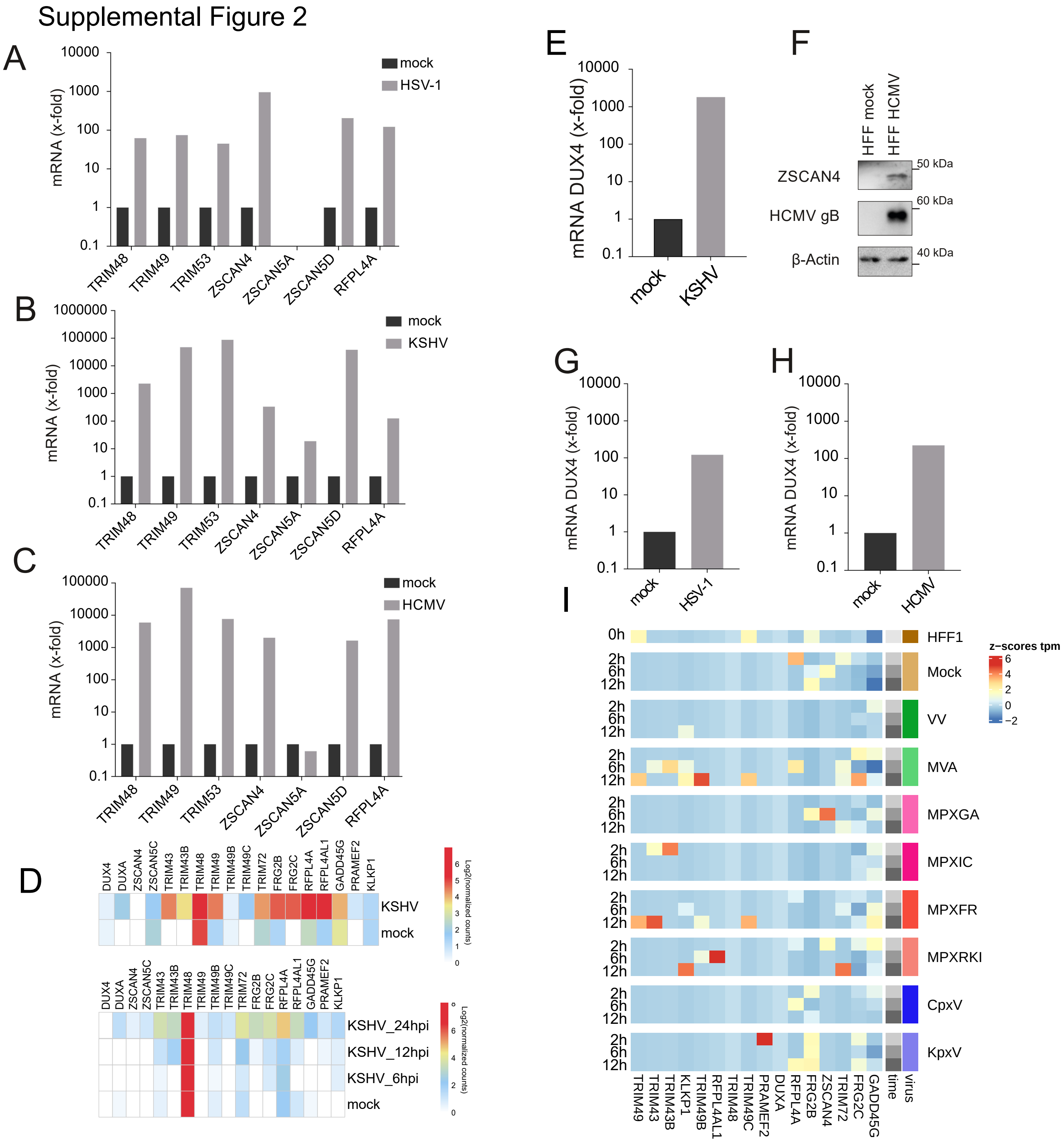

Fig. S2. A-C. qPCR analysis of DUX4 target gene expression upon infection of 293T cells with HSV-1 MOI 5 for 18 h. **(A)**, upon lytic reactivation of KSHV in iSLK cells at 5d p.i. **(B)** and upon expression of HFF cells with HCMV MOI 1 for 6d.**(C)** x-fold activation of indicated genes relativ to HPRT**.** Presented data is the mean value of n=2 technical replicates. **D** Upper panel: RNA-seq. analysis of BCBL-1 cells stimulated for KSHV reactivation (GSE179727). The heatmap shows the expression of DUX4 and DUX4 target genes. Lower panel: RNA-seq. analysis of the recombinant PEL cell line TRExBCBL1-3xFLAG-RTA stimulated with Doxycycline for 6h, 12h and 24h (GSE123898). The heatmap shows the expression of DUX4 and DUX4 target genes. **E** mRNA expression of DUX4 in iSLK cells with reactivated KSHV infection, harvested after 5 d. x-fold activation of DUX4 relativ to HPRT. Presented data is the mean value of n=2 technical replicates. **F** HFF cells infected with HCMV for 6d with MOI 1. Western blot analysis of ZSCAN4. HCMV glycoprotein B (gB) was used as marker for infection. One representative experiment out of n=2 **G** mRNA expression of DUX4 in 293T upon infection with HSV-1 for 18 h with MOI 10. x-fold activation of DUX4 relativ to HPRT. Presented data is the mean value of n=2 technical replicates. **H.** mRNA expression of DUX4 target genes in HFF infected with HCMV for 6 d with MOI 1. x-fold activation of DUX4 relativ to HPRT. Presented data is the mean value of n=2 technical replicates. **I** RNA-seq. analysis of HFF1 cells infected with vaccinia virus (VV), modified vaccinia Ankara (MVA), Cowpoxvirus (CpxV), Camelpoxvirus (KpxV), mpox virus Gabun (clade I, MPXGA), mpox virus Ivory Coast (clade 2a, MPXIC), mpox virus Freiburg (clade 2b, MPXFR) and mpox virus RKI (clade 2b, MPXRKI) at 2h, 6h and 12hpi. One representative experiment out of n=5 (A), n=2 (C), n=3 (B), n=4 (E,G,H) is shown.

Supplemental Figure 3

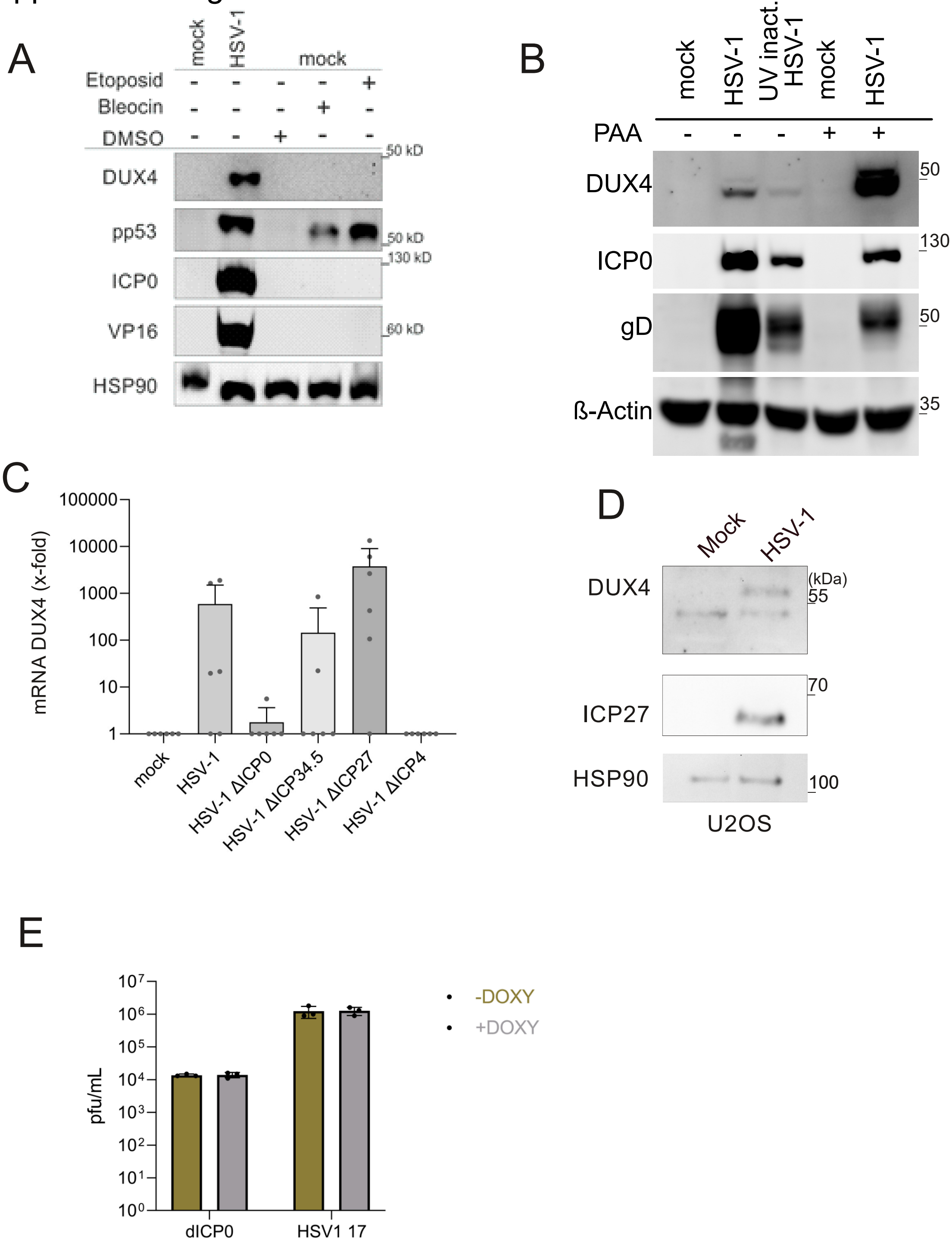

Fig. S3. A Western blot of DUX4 expression in 239T cells infected with HSV-1 for 18 h or treated with Bleocin or Etoposid. Bleocin and Etoposid were used to induce DNA damage in the cells and start the DDR. HSV-1 ICP0 and Vp16 were used as markers for infection. HSP90 was used as loading control. One representative out of n=3. **B** Western blot of DUX4 expression in 239T cells infected with wt HSV-1, UV-inactivated HSV-1 or treated with Polymerase inhibitor Phosphonoacetic acid (PAA). HSV-1 ICP0 and gD were used as markers for infection. ß-Actin was used as loading control. One representative out of n=3. **C** qRT-PCR of DUX4 expression relative to HPRT from primary HFF cells infected with HSV-1, HSV-1 ΔICP0 (ICP4-YFP), HSV-1 ΔICP34.5, HSV-1 ΔICP4, HSV-1 ΔICP27 for 24 h (MOI 10). Data are biolocial replicates (n=6) and presented as mean values +/- SD. **D** Western Blot of U2OS cells infected with HSV-1 GFP(F-strain, MOI 1 for 20 hours). One representative out of n=3. **E** Plaque Assays from viral supernatant of HFF cells infected with HSV-1 wt (strain 17) and HSV-1 delta-ICP0. Cells were transduced with a Doxycycline inducible DUX4 construct and induced with Doxycycline (1µg/ml) as indicated. Viral titer in plaque-forming units (pfu/ml). Data are biolocial replicates (n=3) and presented as mean values +/- SD.

Supplemental Figure 4

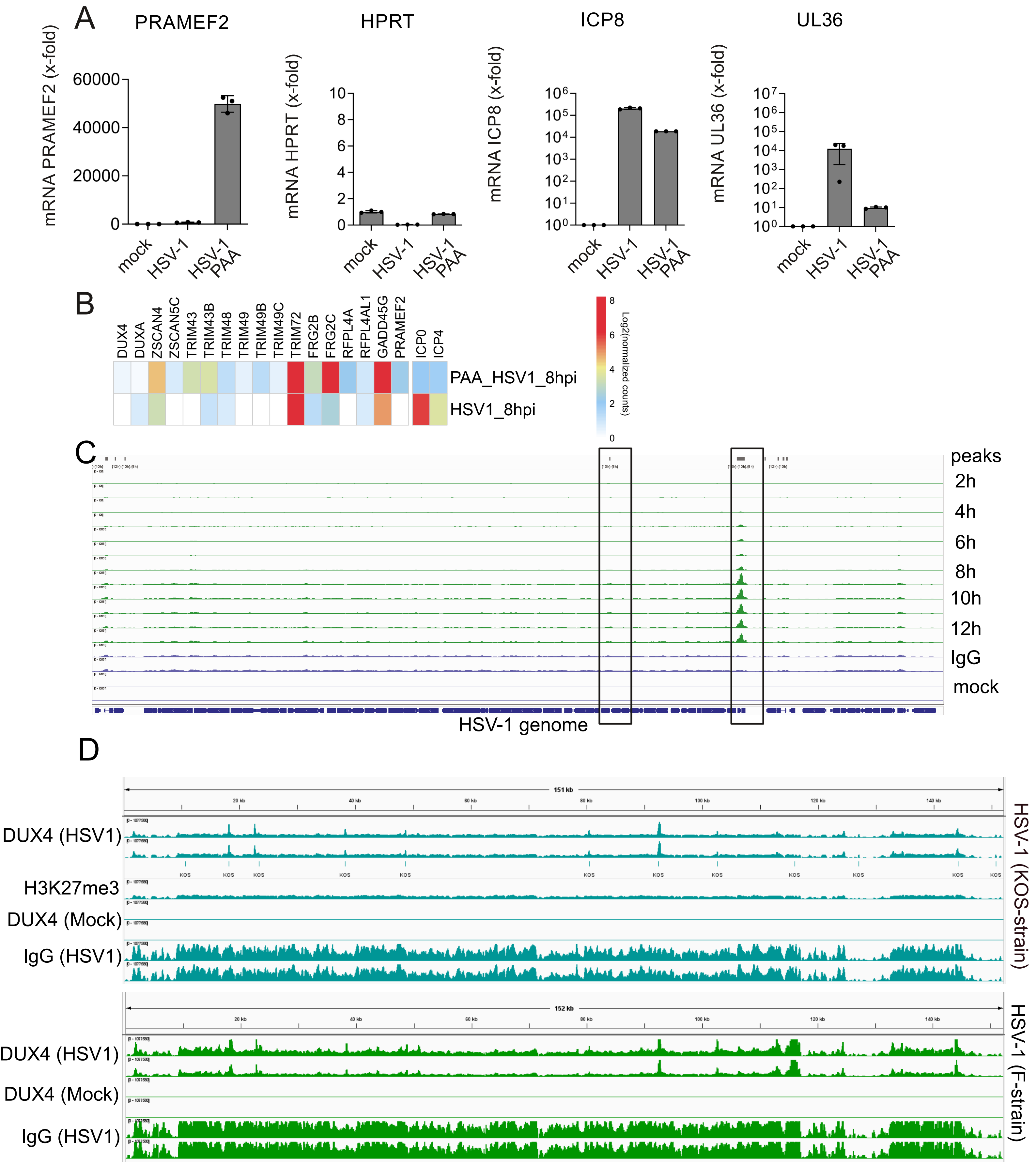

Fig. S4. A qRT-PCR analysis of cellular genes PRAMEF2 and HPRT as well as viral genes ICP8 and UL36 in HDF-TERT cells untreated or treated with PAA and infected with HSV-1 (MOI 0.1). Values are biological replicates (n=3) and presented as mean fold induction +/- SD relative to uninfected control cells. **B** RNA-seq. analysis of HFF cells infected with HSV-1 treated with PAA (F-strain, MOI 10, data from GSM5608614). The heatmap shows the expression of DUX4 and DUX4 target genes. **C** DUX4 CUT&Tag analysis of HFF1 cells infected with HSV-1 GFP (F-strain, MOI1) at 2,4,6,8,10,12h post infection. Samples with IgG antibody were included as controls. **D** CUT&RUN with a DUX4-specific antibody in HFF1 cells infected with HSV-1 at 12 hpi performed in duplicates. IgG and H3K27me3-specific antibodies were included as controls. Data was normalized to Drosophila Spike-in DNA. Lines labeled with KOS indicate the DUX4 motif sites in the HSV-1 genome determined with FIMO.

Supplemental Figure 5

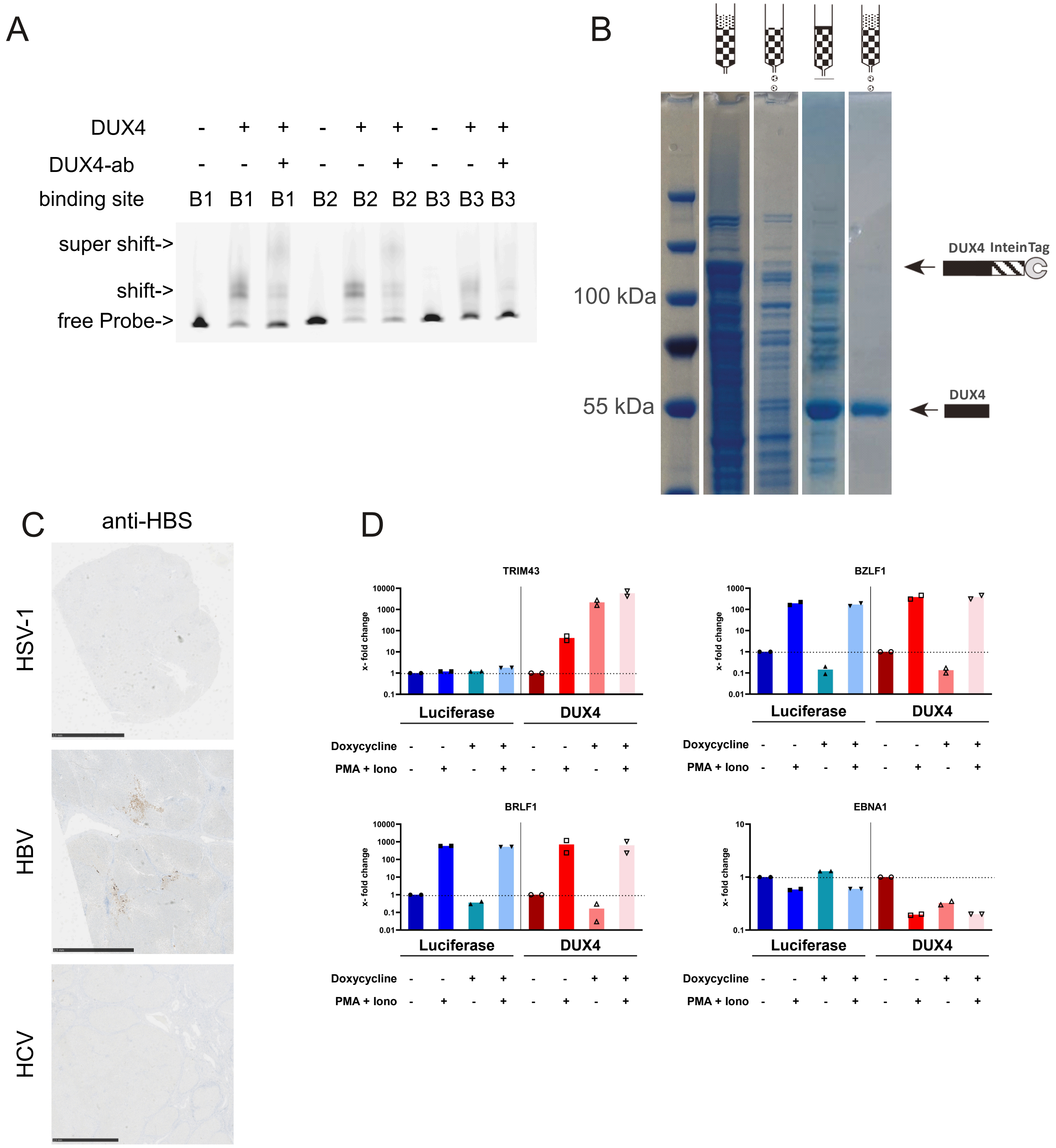

Figure S5. A Electrophoretic Mobility Shift Assay (EMSA) of ~600bp fragments of fluorescently labelled viral DNA amplified from the KSHV genome containing one DUX4 binding motif (DUX4-BS), incubated with purified DUX4 protein (shift) and DUX4 antibody (super-shift). One representative out of n=3. **B** Expression and purification of full-length DUX4 protein in E.coli. Coloidal coomassie gels of purification steps. Intein-tagged DUX4 protein was expressed in E.coli and purified with chitin columns on a Äkta pure system. Cleavage of the Intein-tag was induced by DTT and the cleaved protein further purified by size-exclusion (SE) chromatography. **C** Immunohistochemistry staining of liver biopsies from hepatitis patients with Herpes-simplex virus 1 (HSV-1), Hepatitis B virus (HBV) and Hepatitis C virus (HCV). Slices were stained with antibodies specific for HBS antigen. Scale bar is 2,5mm. **D** qPCR analysis of TRIM43, BZLF1, BLRF1 and EBNA1 gene expression relative to HPRT in Raji cells. Cells were transduced with either a Doxycycline inducible Luciferase construct or a Doxycycline inducible DUX4 construct. Cells were treated with Doxycycline (1µg/ml) for 24h and PMA/Ionomycin (25ng/ml / 1µg/ml) for 24h as indicated. One representative experiment out of n=2, values shown are technical duplicates (n=2).

Supplemental Figure 6

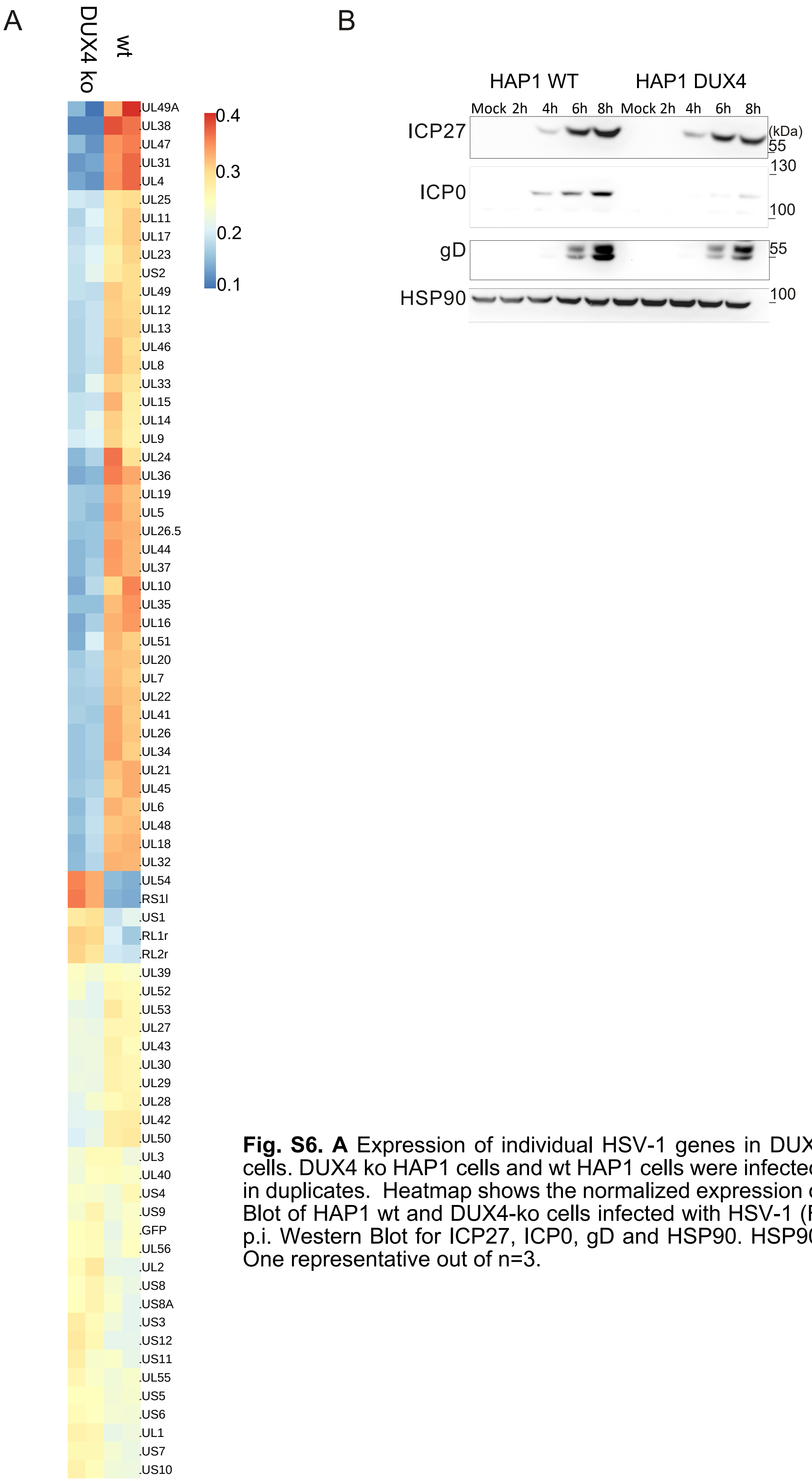

Fig. S6. A Expression of individual HSV-1 genes in DUX4 ko HAP1 cells vs. wt HAP1 cells. DUX4 ko HAP1 cells and wt HAP1 cells were infected with HSV-1 at MOI of 1 for 8h in duplicates. Heatmap shows the normalized expression of all HSV-1 genes. **B** Western Blot of HAP1 wt and DUX4-ko cells infected with HSV-1 (F-strain, MOI1) at 2,4,6 and 8h p.i. Western Blot for ICP27, ICP0, gD and HSP90. HSP90 was used as loading control. One representative out of n=3.
