# Supplementary material for: Herpesviruses mimic zygotic genome activation to promote viral replication
Source: Nat Commun. 2025 Jan 16;16:710. doi: 10.1038/s41467-025-55928-5 (PMC11735616; doi:10.1038/s41467-025-55928-5)
Supplement: Supplementary file 3 — Description of Additional Supplementary Files [file 41467_2025_55928_MOESM3_ESM.pdf]

File Name: Supplementary Data 1
Description: Peak calling results of DUX4 CUT&Tag after infection with HSV-1 GFP (F-strain)

File Name: Supplementary Data 2
Description: Peak calling results of DUX4 CUT&RUN after infection with HSV-1 GFP (F-strain)

File Name: Supplementary Data 3
Description: Peak calling results of DUX4 CUT&RUN after infection with HSV-1 KOS

File Name: Supplementary Data 4
Description: Predicted DUX4 binding sites in herpesviral genomes

File Name: Supplementary Data 5
Description: Peak calling results of DUX4 ChIP-Sequencing after HSV1 infection

File Name: Supplementary Data 6
Description: Normalized counts embryonic expression profile

File Name: Supplementary Data 7
Description: Expression pattern of embryonic genes during embryonic genome activation compared to expression pattern in DUX4 overexpressing and HSV-1 infected cells.

File Name: Supplementary Data 8
Description: Differentially expressed genes of 8 cell stage, HSV1 infection and FSHD

File Name: Supplementary Data 9
Description: Sequence of DUX4 nanobodies
