# Supplementary material for: Herpesviruses mimic zygotic genome activation to promote viral replication
Source: Nat Commun. 2025 Jan 16;16:710. doi: 10.1038/s41467-025-55928-5 (PMC11735616; doi:10.1038/s41467-025-55928-5)
Supplement: Supplementary file 14 — Source Data [file 41467_2025_55928_MOESM14_ESM.zip › Supplemental Figure 5.docx]

**Supplemental Figure 5A**

**
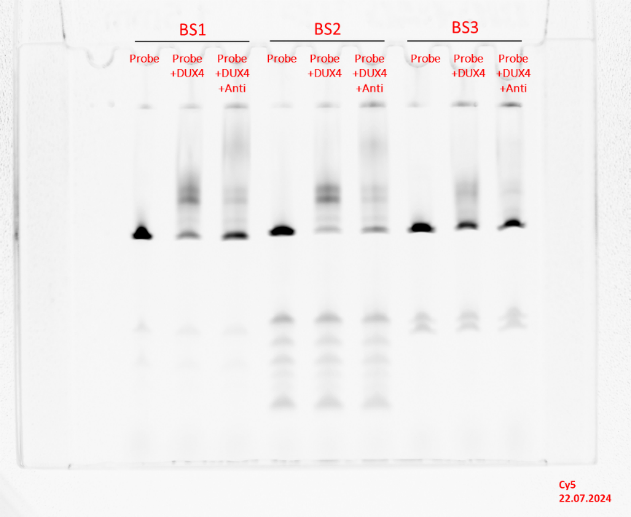
**

**Supplemental Figure 5B**

**
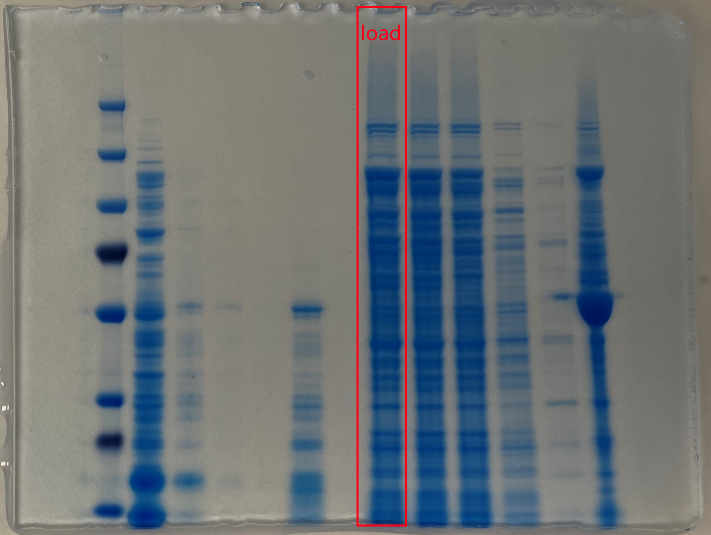

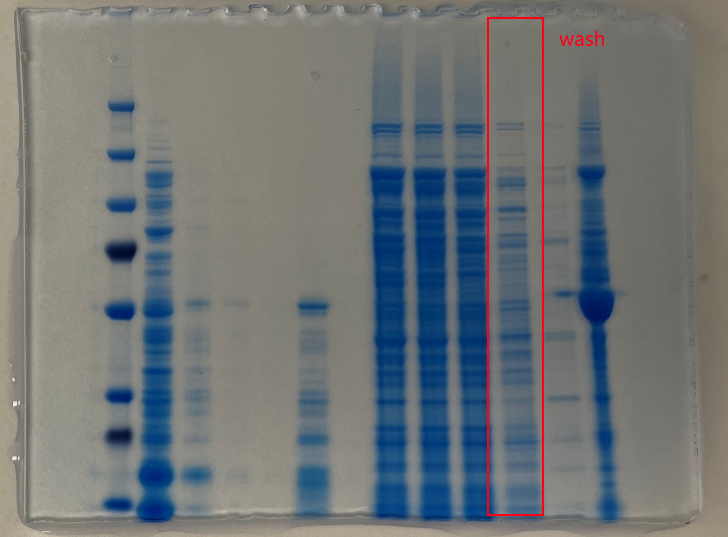

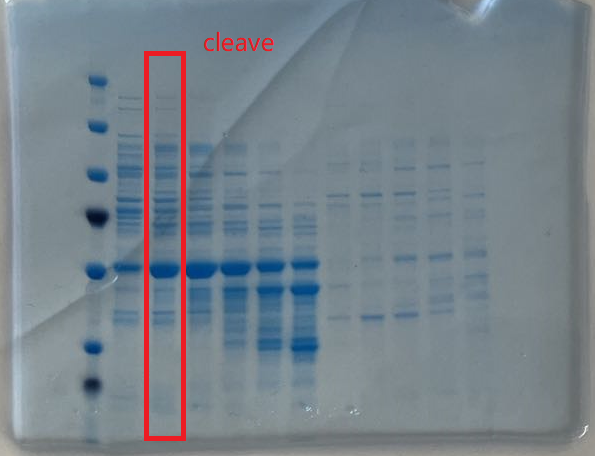

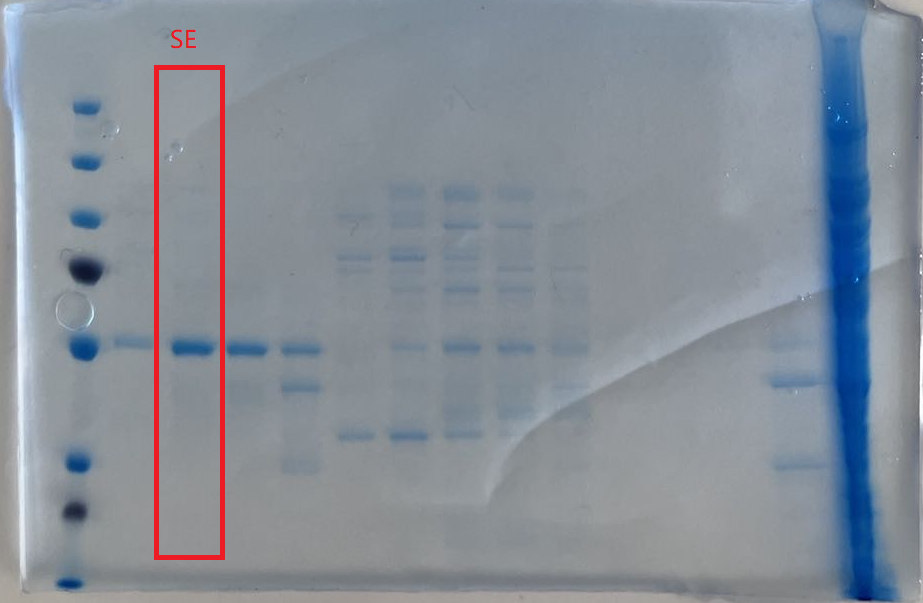
**

**Supplemental Figure 5D**

|  | **Luciferase unstimulated** | | **Luciferase +PMA25+I** | | **Luciferase +doxycycline** | | **Luciferase +doxy+PMA25+I** | | **DUX4 unstimulated** | | **DUX4 +PMA25+I** | | **DUX4 +doxycycline** | | **DUX4 +doxy+PMA25+I** | |
| --- | --- | --- | --- | --- | --- | --- | --- | --- | --- | --- | --- | --- | --- | --- | --- | --- |
| **BZLF1** | **1** | **1** | **162.58** | **225.7** | **0.09** | **0.2** | **144.86** | **194.9** | **1** | **1** | **461.74** | **307.9** | **0.17** | **0.1** | **455.78** | **311.7** |
| **BRLF1** | **1** | **1** | **590.07** | **579.6** | **0.42** | **0.3** | **523.89** | **510.9** | **1** | **1** | **237.9** | **1178.9** | **0.03** | **0.3** | **229.3** | **1046.4** |
| **EBNA1** | **1** | **1** | **0.57** | **0.6** | **1.29** | **1.3** | **0.6** | **0.6** | **1** | **1** | **0.19** | **0.2** | **0.35** | **0.3** | **0.2** | **0.2** |
| **TRIM43** | **1** | **1** | **1.21** | **1.2** | **1.24** | **1.2** | **1.76** | **1.8** | **1** | **1** | **56.1** | **34.6** | **2744.63** | **1584.8** | **7402.18** | **4200.6** |
