# Supplementary material for: Herpesviruses mimic zygotic genome activation to promote viral replication
Source: Nat Commun. 2025 Jan 16;16:710. doi: 10.1038/s41467-025-55928-5 (PMC11735616; doi:10.1038/s41467-025-55928-5)
Supplement: Supplementary file 14 — Source Data [file 41467_2025_55928_MOESM14_ESM.zip › Supplemental Figure 4.docx]

**Supplemental Figure 4A**

| HPRT | | | | |
| --- | --- | --- | --- | --- |
| mock |  | 1 | 1,109569 | 0,946058 |
| HSV-1 |  | 0,044194 | 0,038741 | 0,037681 |
| HSV-1 PAA |  | 0,82932 | 0,840896 | 0,852635 |

|  |  | PRAMEF2 | | |
| --- | --- | --- | --- | --- |
| mock |  | 1 | 1 | 1 |
| HSV-1 |  | 814,6294 | 625,9918 | 596,3436 |
| HSV-1 PAA |  | 51063,33 | 52498,92 | 46020,85 |

|  |  | UL36 | | |
| --- | --- | --- | --- | --- |
| mock |  | 1 | 1 | 1 |
| HSV-1 |  | 229,1264 | 19755,96 | 17682,08 |
| HSV-1 PAA |  | 10,55606 | 9,986644 | 8,514961 |

|  |  | ICP8 | | |
| --- | --- | --- | --- | --- |
| mock |  | 1 | 1 | 1 |
| HSV-1 |  | 187951,4 | 225067,8 | 207104,6 |
| HSV-1 PAA |  | 18820,27 | 18820,27 | 18820,27 |
