# Supplementary material for: Herpesviruses mimic zygotic genome activation to promote viral replication
Source: Nat Commun. 2025 Jan 16;16:710. doi: 10.1038/s41467-025-55928-5 (PMC11735616; doi:10.1038/s41467-025-55928-5)
Supplement: Supplementary file 14 — Source Data [file 41467_2025_55928_MOESM14_ESM.zip › Supplemental Figure 3.docx]

**Supplemental Figure 3A**


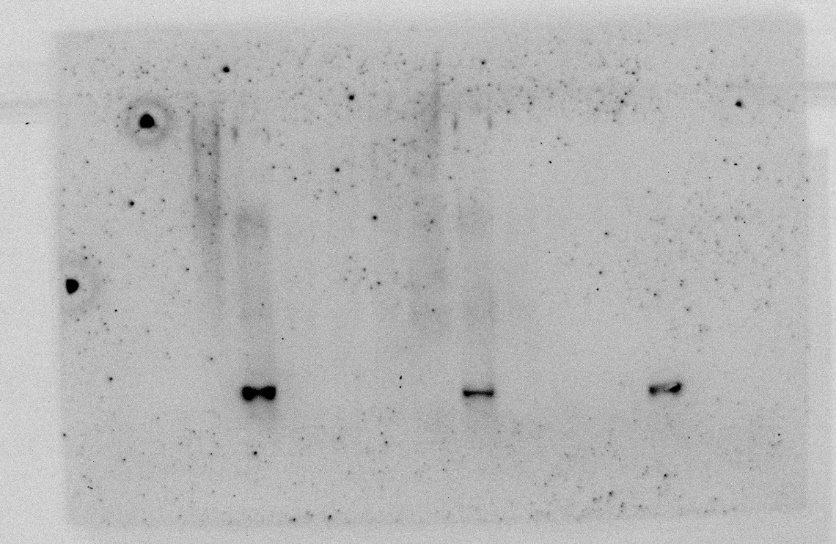


DUX4

Eto

Bleo

DMSO

HSV-1

Mock


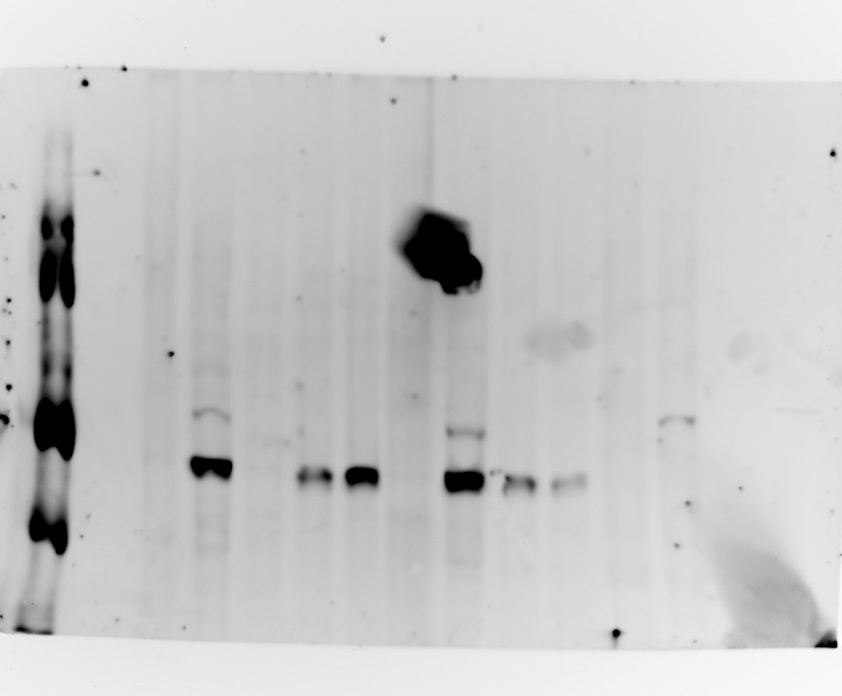


Eto

Bleo

DMSO

HSV-1

Mock

pp53


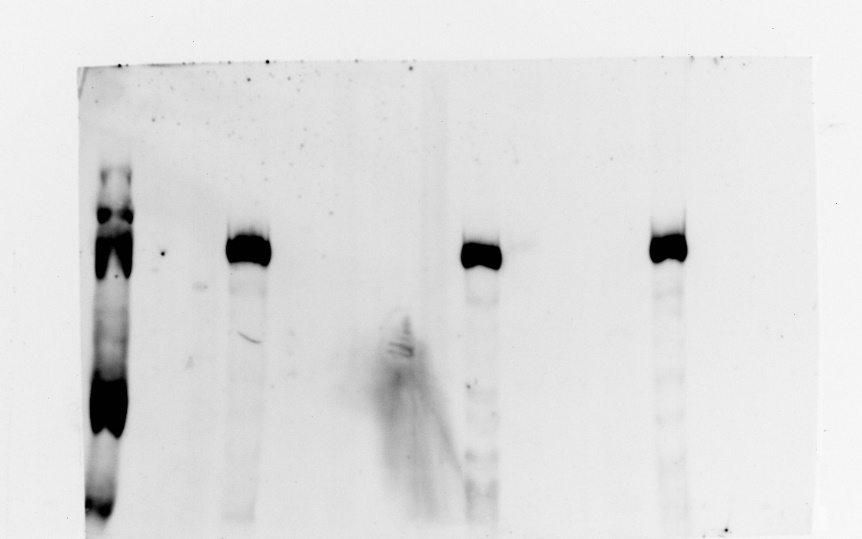


Eto

Bleo

DMSO

HSV-1

Mock

ICP0


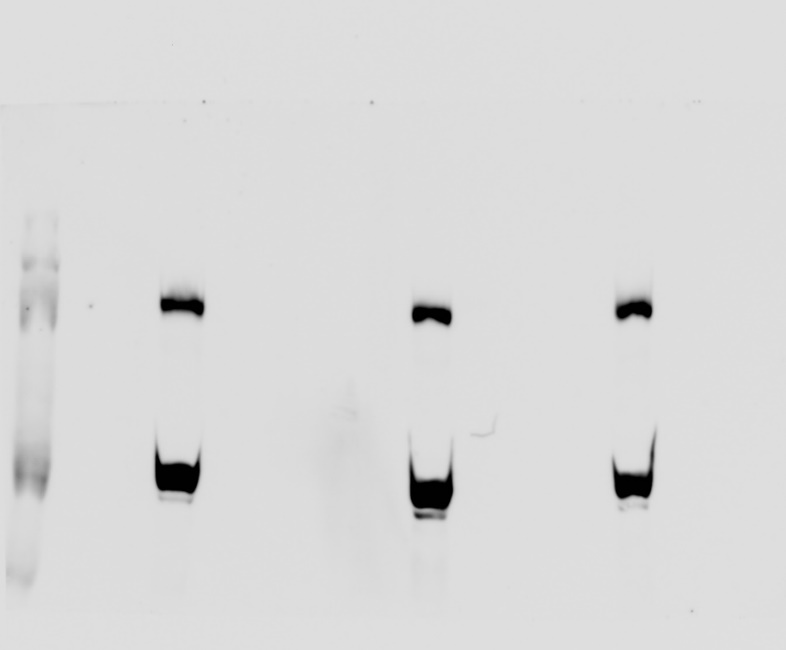


Eto

Bleo

DMSO

HSV-1

Mock

VP16


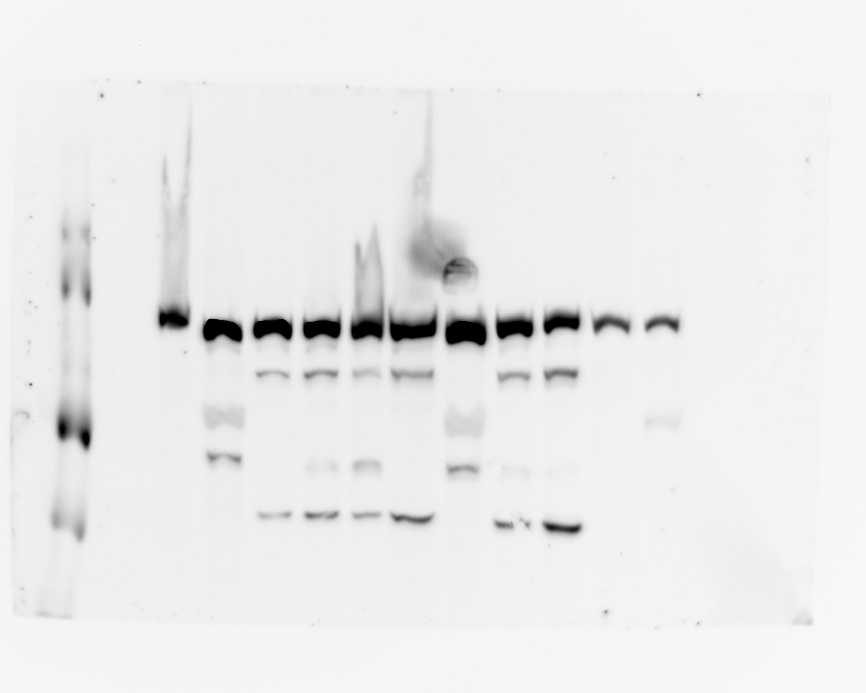


Eto

Bleo

DMSO

HSV-1

Mock

HSP90

**Supplemental Figure 3B**


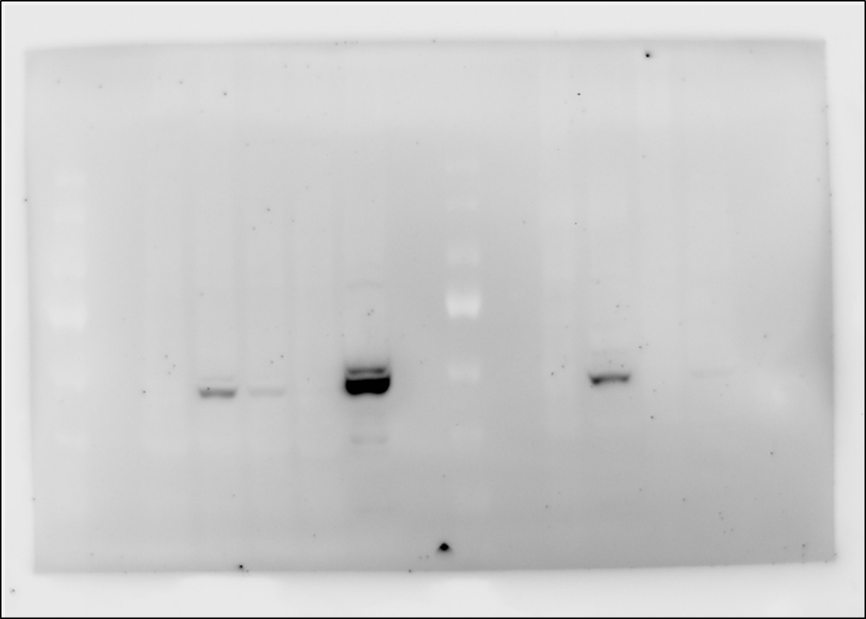


HSV-1 PAA

Mock PAA

UV HSV-1

HSV-1

Mock

DUX4


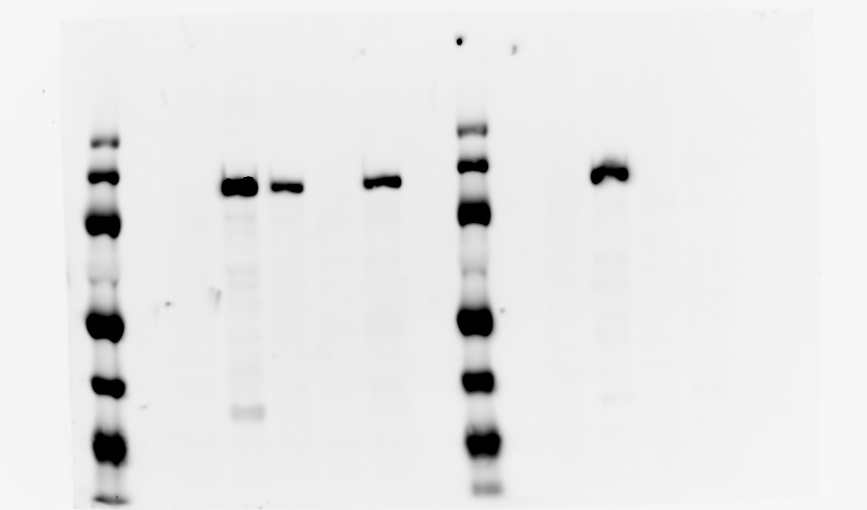


HSV-1 PAA

Mock PAA

UV HSV-1

HSV-1

Mock

ICP0


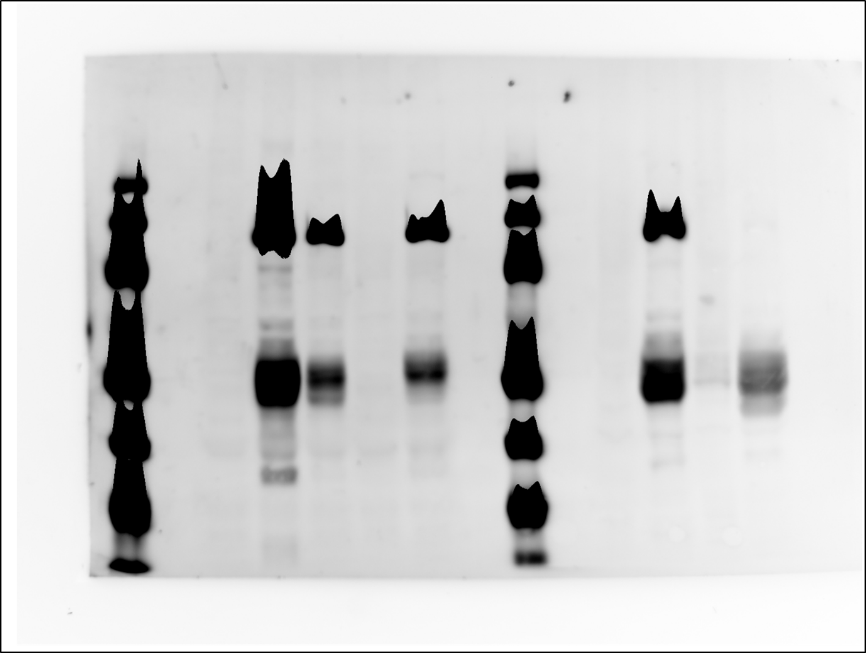


HSV-1 PAA

Mock PAA

UV HSV-1

HSV-1

Mock

gD


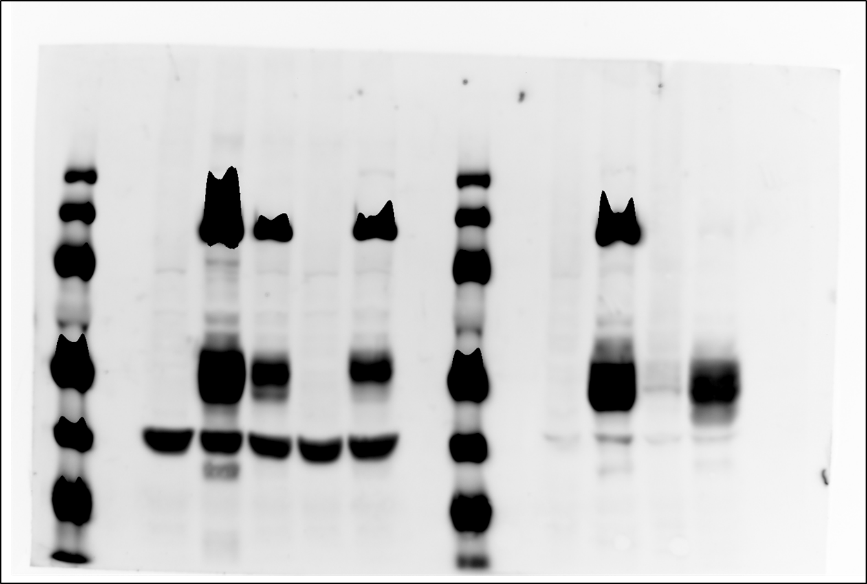


Mock

HSV-1

UV HSV-1

Mock PAA

HSV-1 PAA

ß-Actin

**Supplemental Figure 3C**

|  | mock | HSV-1 | HSV-1 ΔICP0 | HSV-1 ΔICP34.5 | HSV-1 ΔICP27 | HSV-1 ΔICP4 |
| --- | --- | --- | --- | --- | --- | --- |
|  | 1 | 1 | 1 | 1 | 13365,94892 | 1 |
|  | 1 | 21,36629 | 1 | 22,33413 | 106,61261 | 1 |
|  | 1 | 1644,3775 | 1 | 1 | 429,14215 | 1 |
|  | 1 | 19,66857 | 1 | 1 | 1 | 1 |
|  | 1 | 1 | 1 | 843,46787 | 6035,31924 | 1 |
|  | 1 | 1883,88689 | 5,57451 | 1 | 2641,40708 | 1 |
|  |  |  |  |  |  |  |

**Supplemental Figure 3D**

**
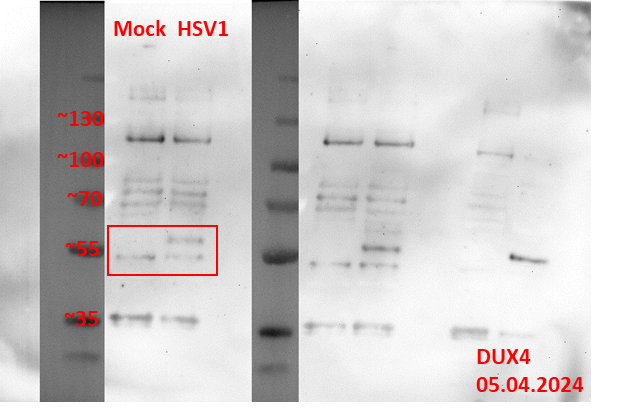
**

**
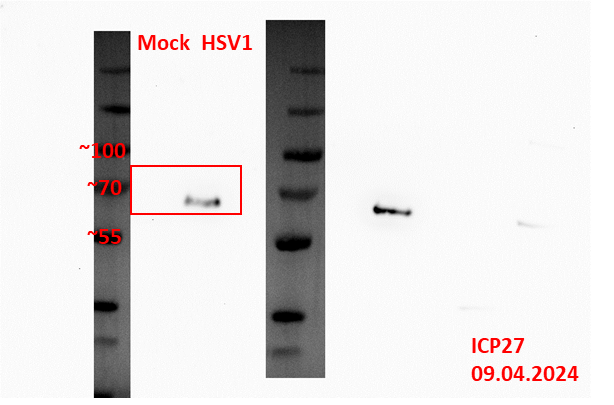
**

**
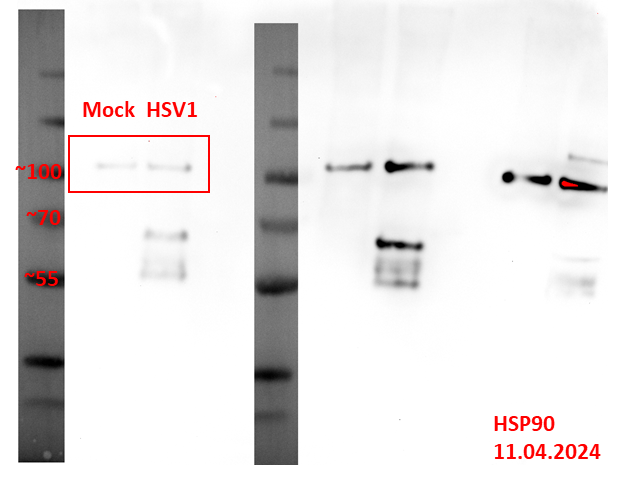
**

**Supplemental Figure 3E**

|  | -DOXY | | | +DOXY | | |
| --- | --- | --- | --- | --- | --- | --- |
| dICP0 | 15000 | 13000 | 13000 | 15000 | 11000 | 16000 |
| HSV1 17 | 1800000 | 1000000 | 900000 | 1600000 | 1300000 | 900000 |
