# Supplementary material for: Herpesviruses mimic zygotic genome activation to promote viral replication
Source: Nat Commun. 2025 Jan 16;16:710. doi: 10.1038/s41467-025-55928-5 (PMC11735616; doi:10.1038/s41467-025-55928-5)
Supplement: Supplementary file 14 — Source Data [file 41467_2025_55928_MOESM14_ESM.zip › Supplemental Figure 2.docx]

**Supplemental Figure 2A**

|  | Mock 18h | | | HSV-1 18h | | |
| --- | --- | --- | --- | --- | --- | --- |
|  | Mean | +Error | -Error | Mean | +Error | -Error |
| TRIM48 | 1 | 0,1767 | 0,15017 | 62,28935 | 2,665051 | 2,555698 |
| TRIM49 | 1 | 0,89906 | 0,47342 | 74,25221 | 6,435608 | 5,92231 |
| TRIM53 | 1 | 0,13058 | 0,1155 | 44,58157 | 2,792439 | 2,627842 |
| ZSCAN4 | 1 |  |  | 957,3411 | 36,55359 | 35,20923 |
| ZSCAN5A |  |  |  |  |  |  |
| ZSCAN5D | 1 | 0,52332 | 2,5557 | 204,2696 | 16,86143 | 0,3435364 |
| RFPL4A | 1 | 1,05056 | 0,04813 | 121,7657 | 10,96143 | 10,05617 |

**Supplemental Figure 2B**

|  | Mock 5d | | | KSHV 5d | | |
| --- | --- | --- | --- | --- | --- | --- |
|  | Mean | +Error | -Error | Mean | +Error | -Error |
| TRIM48 | 1 |  |  | 2284,001 | 339,8865 | 295,859 |
| TRIM49 | 1 | 3,107e-002 | 3,014e-002 | 47209,29 | 6478,543 | 5696,773 |
| TRIM53 | 1 | 3,107e-002 | 3,014e-002 | 87668,63 | 12623,72 | 11034,78 |
| ZSCAN4 | 1 |  |  | 335,538 | 46,03201 | 40,47879 |
| ZSCAN5A | 1 | 0,501748 | 3,341093e-001 | 18,77992 | 2,602131 | 2,28546 |
| ZSCAN5D | 1 | 3,107e-002 | 3,014e-002 | 38062,98 | 27823,46 | 16073,78 |
| RFPL4A | 1 | 1,1724e-001 | 1,0494e-001 | 125,1591 | 19,4557 | 16,83823 |

**Supplemental Figure 2C**

|  | Mock 6d | | | HCMV 6d | | |
| --- | --- | --- | --- | --- | --- | --- |
|  | Mean | +Error | -Error | Mean | +Error | -Error |
| TRIM48 | 1 | 0,92628 | 4,8087e-001 | 5965,053 | 365,6328 | 344,5154 |
| TRIM49 | 1 | 1,415002e-002 | 1,396e-002 | 71272,24 | 911,5673 | 900,0557 |
| TRIM53 | 1 |  |  | 7653,729 | 279,6501 | 269,7925 |
| ZSCAN4 | 1 | 3,2589e-001 | 2,4579e-001 | 2007,271 | 411,0769 | 341,201 |
| ZSCAN5A | 1 | 1,976247e-001 | 1,650139e-001 | 0,6138681 | 0,25437 | 0,17985 |
| ZSCAN5D | 1 |  |  | 1651,101 | 609,5485 | 445,1934 |
| RFPL4A | 1 | 3,77e-002 | 3,632998e-002 | 7508,066 | 782,5329 | 708,6712 |

**Supplemental Figure 2E**

|  | Mock | | | KSHV | | |
| --- | --- | --- | --- | --- | --- | --- |
|  | Mean | +Error | -Error | Mean | +Error | -Error |
| DUX4 | 1 | 3,106999e-002 | 3,013998e-002 | 1817,321 | 676,959 | 493,2292 |

**Supplemental Figure 2F**

Die Rohdaten zu diesem Blot:


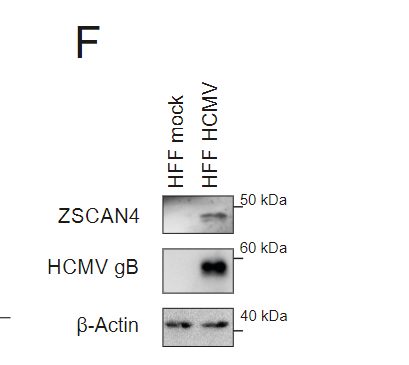


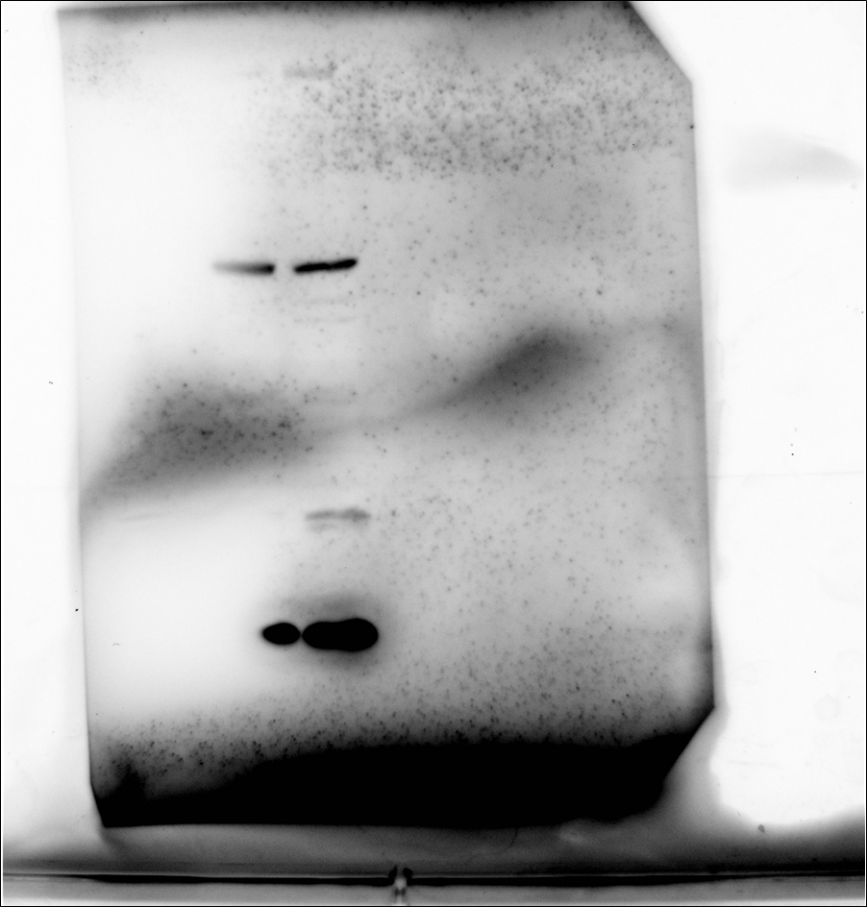


ZSCAN4

CMV

Mock


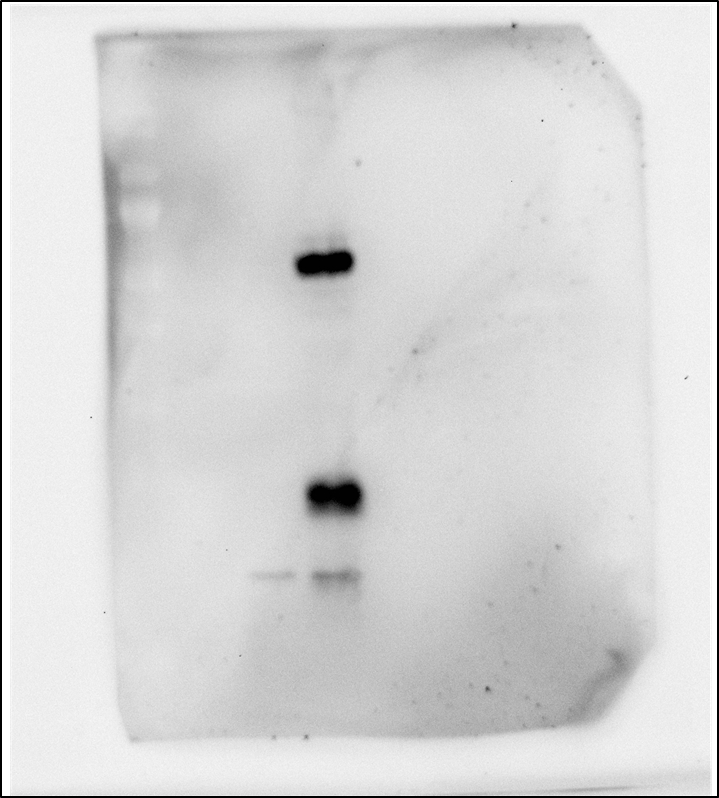


gB

CMV

Mock


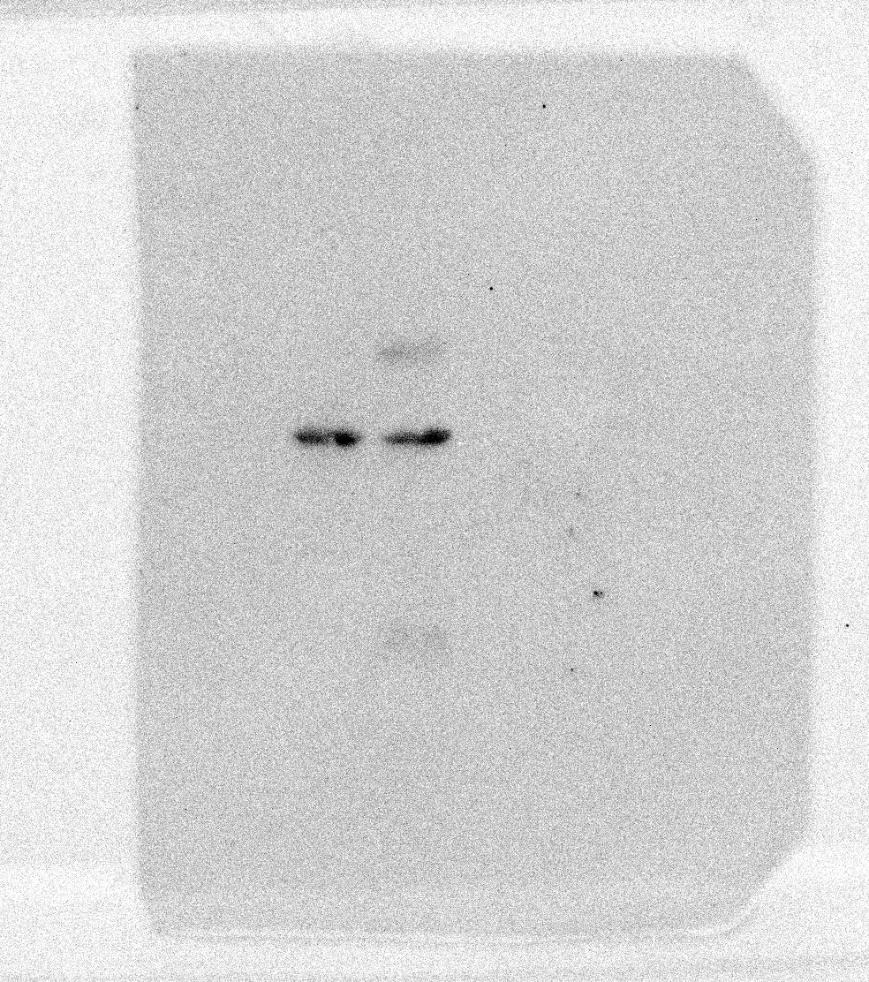


CMV

Mock

ß-Actin

**Supplemental Figure 2G**

|  | Mock | | | HSV-1 | | |
| --- | --- | --- | --- | --- | --- | --- |
|  | Mean | +Error | -Error | Mean | +Error | -Error |
| DUX4 | 1 | 2,0516e-001 | 1,7024e-001 | 120,1643 | 4,88826 | 4,69718 |

**Supplemental Figure 2H**

|  | Mock | | | HCMV | | |
| --- | --- | --- | --- | --- | --- | --- |
|  | Mean | +Error | -Error | Mean | +Error | -Error |
| DUX4 | 1 | 8,129e-002 | 7,518e-002 | 224,8923 | 33,88835 | 29,45054 |
