# Supplementary material for: Herpesviruses mimic zygotic genome activation to promote viral replication
Source: Nat Commun. 2025 Jan 16;16:710. doi: 10.1038/s41467-025-55928-5 (PMC11735616; doi:10.1038/s41467-025-55928-5)
Supplement: Supplementary file 14 — Source Data [file 41467_2025_55928_MOESM14_ESM.zip › Supplemental Figure 1.docx]

**Supplemental Figure 1C**

|  |  | mock | | | HSV-1 | | |
| --- | --- | --- | --- | --- | --- | --- | --- |
| DUX4 |  | 1 | 0,005 | 2,027 | 13,832 | 11,196 | 18,961 |

|  |  | mock | | | HSV-1 | | |
| --- | --- | --- | --- | --- | --- | --- | --- |
| gB |  | 1 | 28,74 | 797,864 | 6,3e+007 | 8,2e+007 | 8,6e+007 |

|  |  | mock | | | HSV-1 | | |
| --- | --- | --- | --- | --- | --- | --- | --- |
| ICP0 |  | 1 | 0,0403 | 37,142 | 320000 | 190000 | 460000 |

**Supplemental Figure 1D**


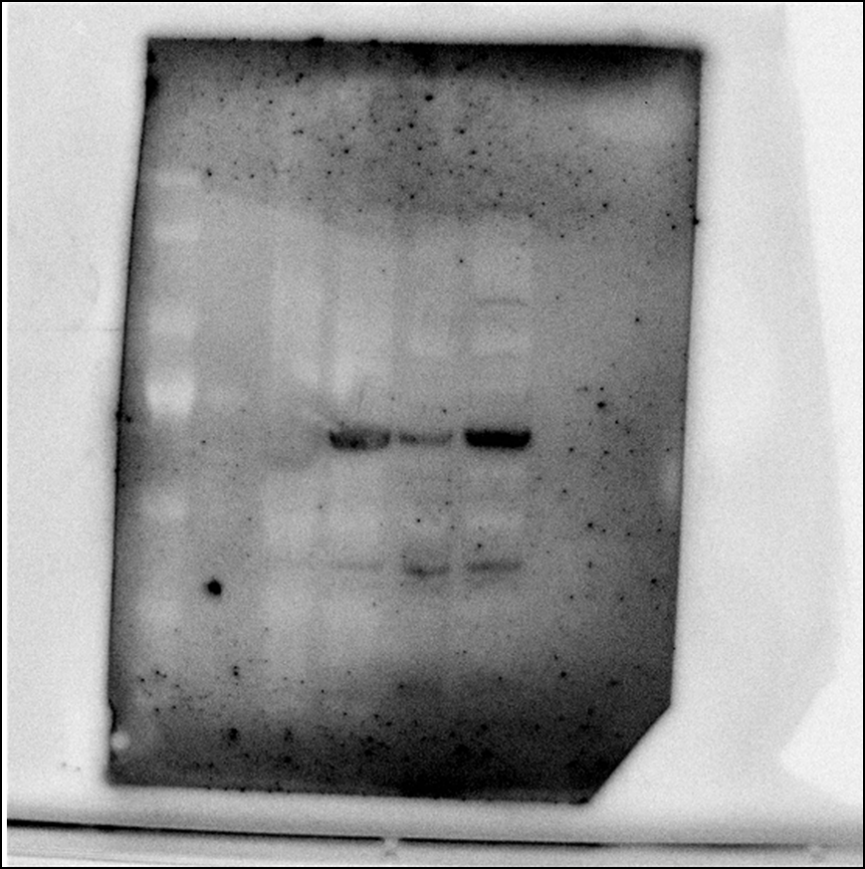


HFF HSV1

HFF Mock

TRIM49

13.04.2018


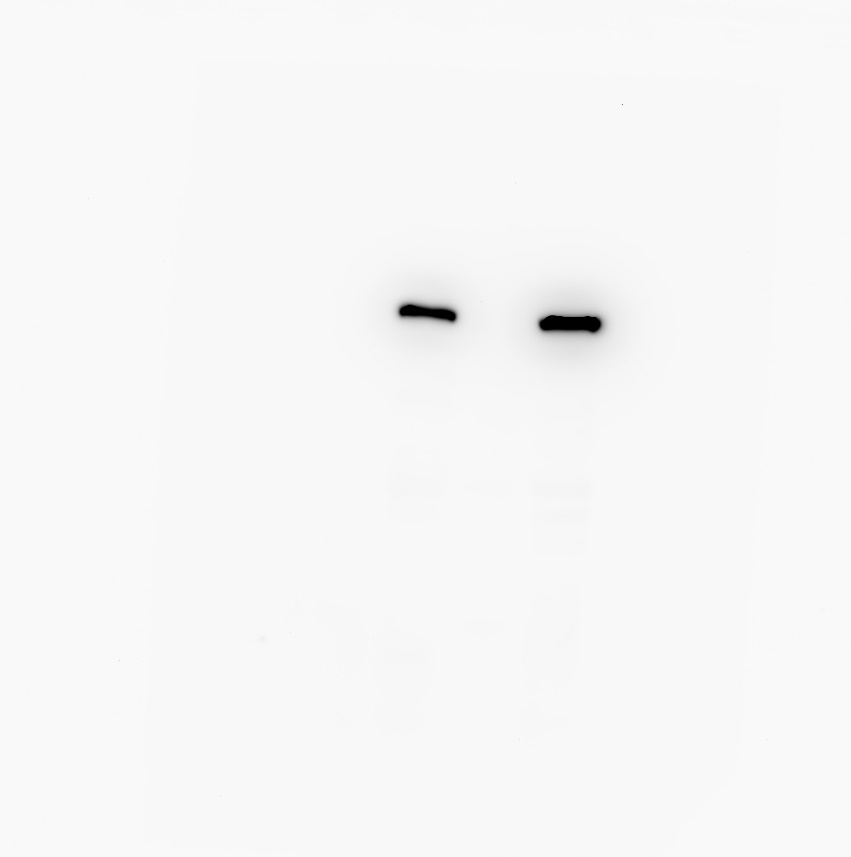


ICP0

17.04.2018

HFF HSV1

HFF Mock


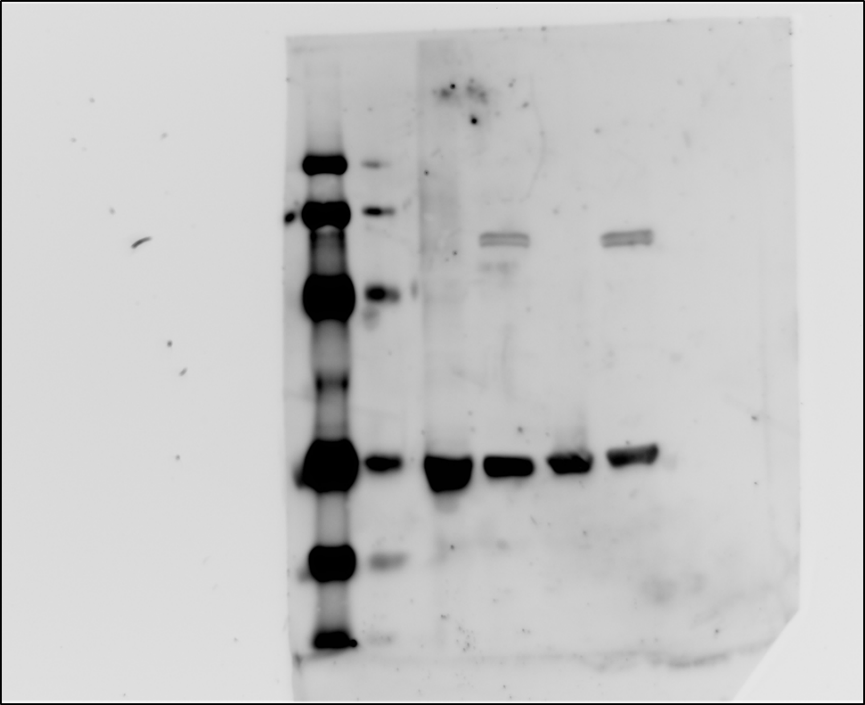


HFF HSV1

HFF Mock

ß-Tubulin

30.04.2018
