# Supplementary material for: Herpesviruses mimic zygotic genome activation to promote viral replication
Source: Nat Commun. 2025 Jan 16;16:710. doi: 10.1038/s41467-025-55928-5 (PMC11735616; doi:10.1038/s41467-025-55928-5)
Supplement: Supplementary file 14 — Source Data [file 41467_2025_55928_MOESM14_ESM.zip › Figure 6.docx]

**Figure 6C**


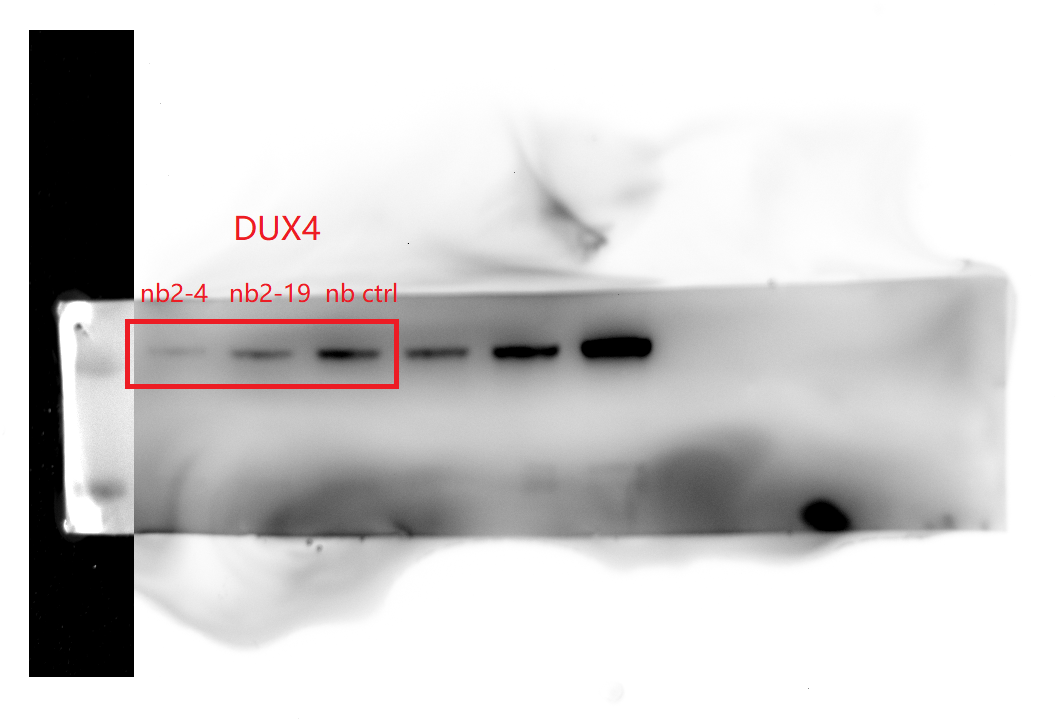
**
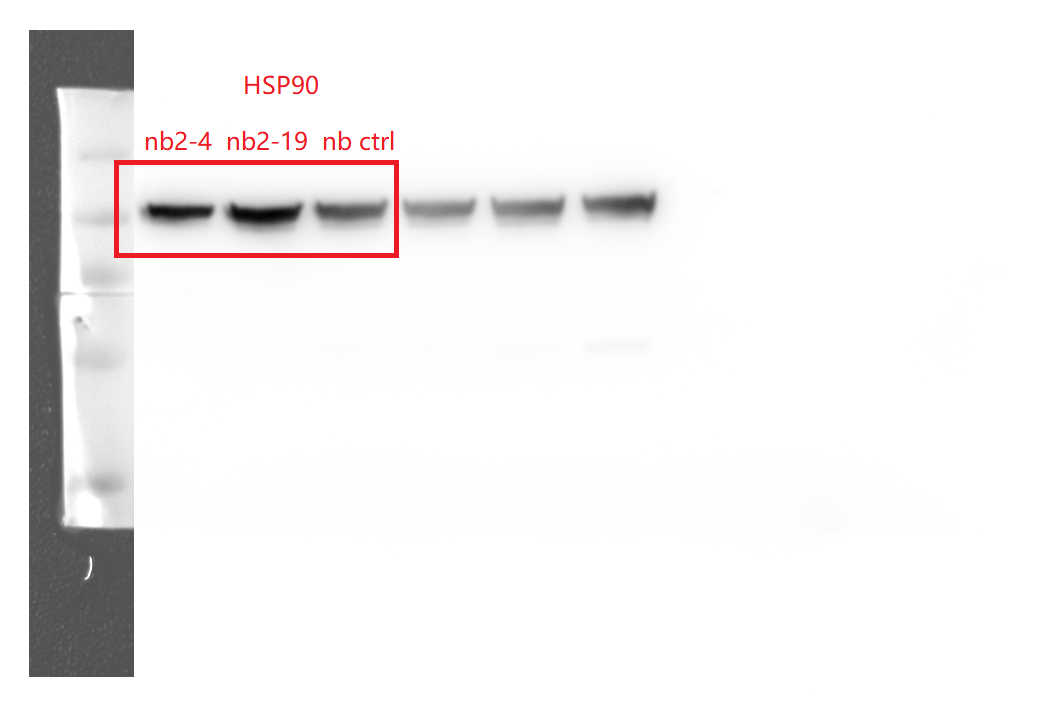
**

**Figure 6D**

**Day1**

**Nb2-4**
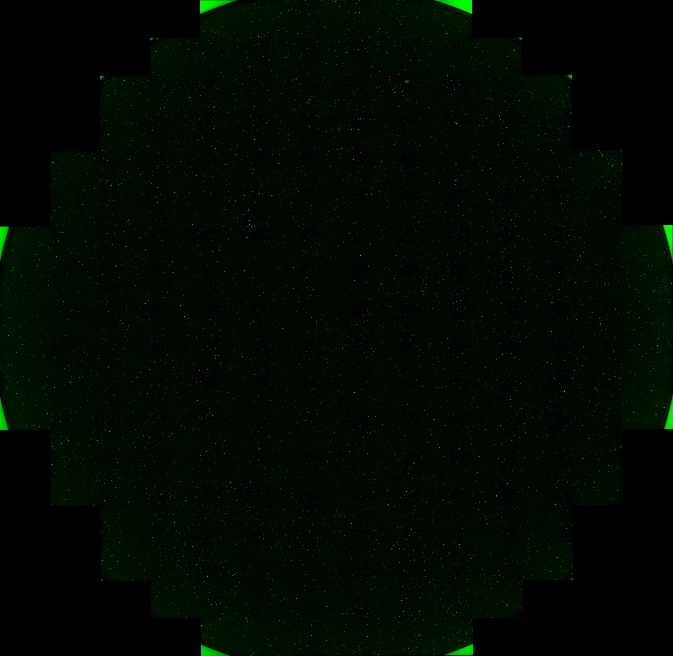


**nb2-19**
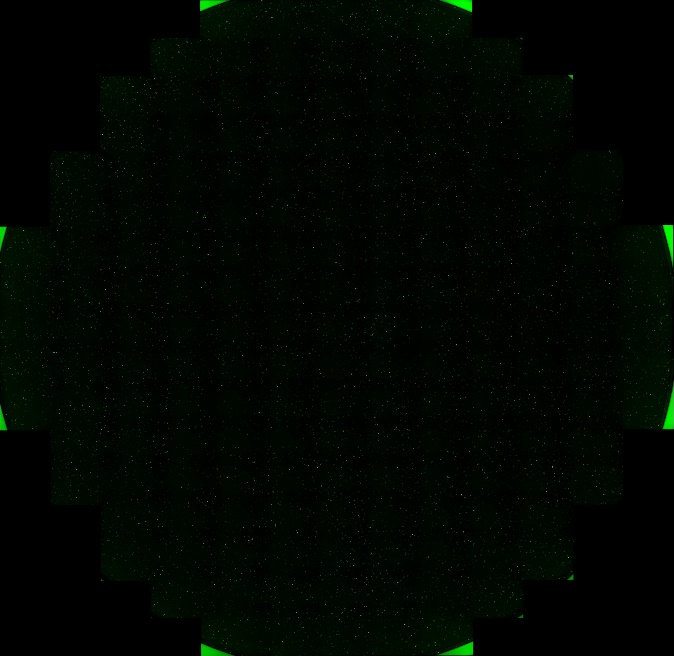


**nb ctrl**
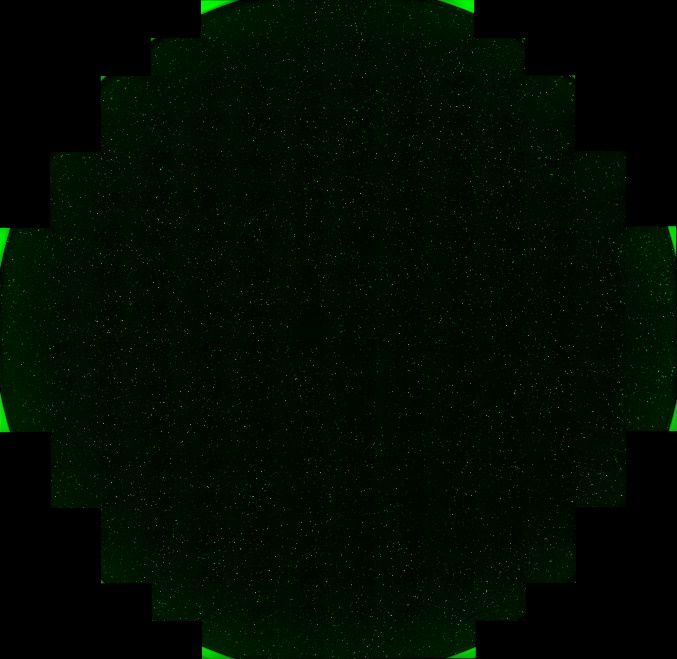


**Day2**

**Nb2-4
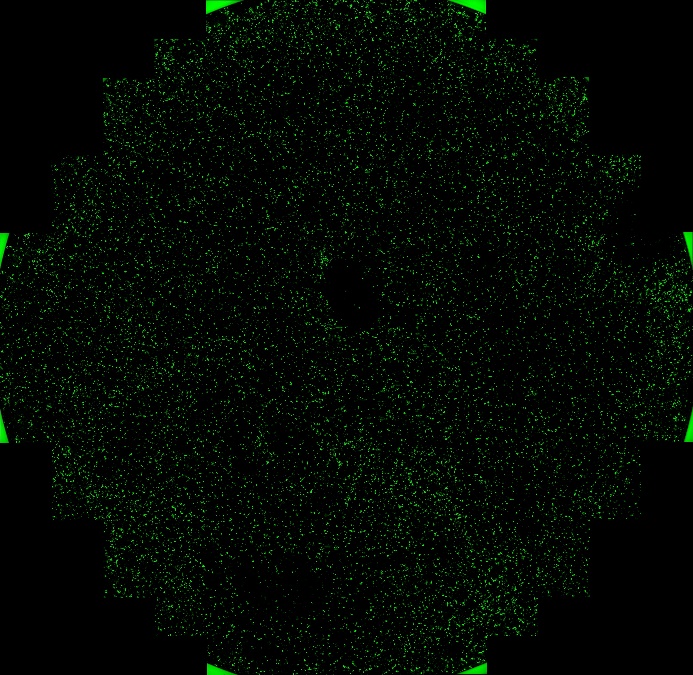
**

**Nb2-19
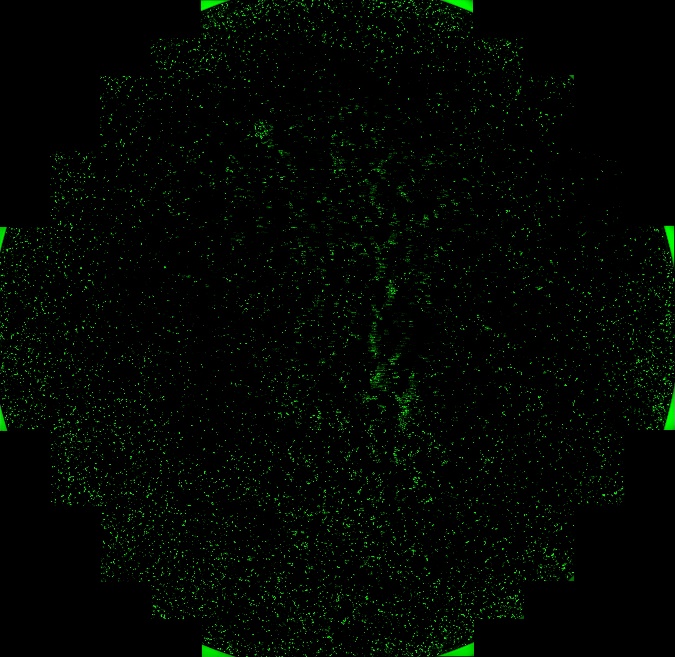
**

**nb ctrl
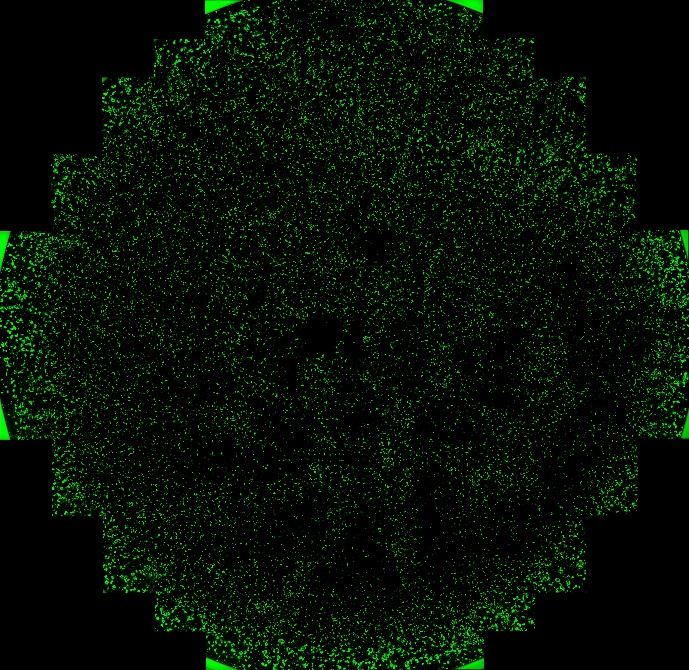
**

**Day3**

**Nb2-4
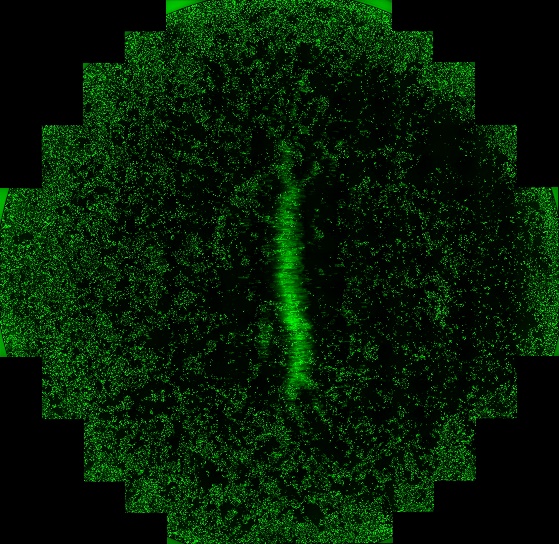
**

**nb 2-19
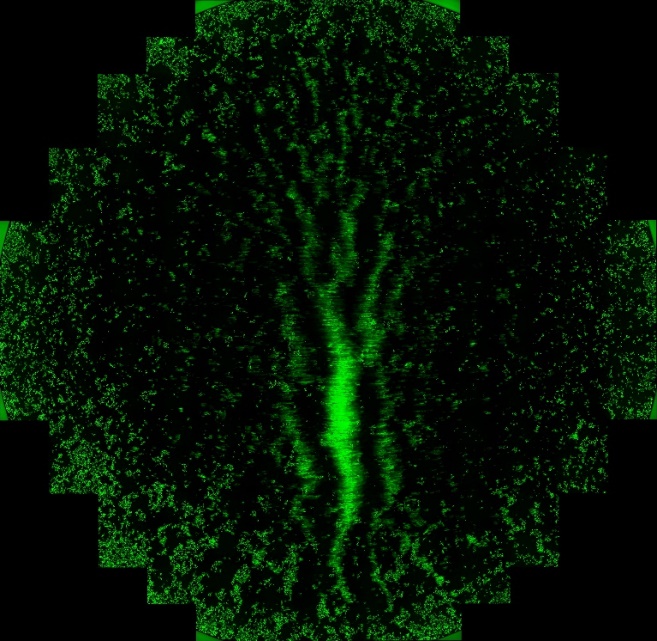
**

**nb ctrl
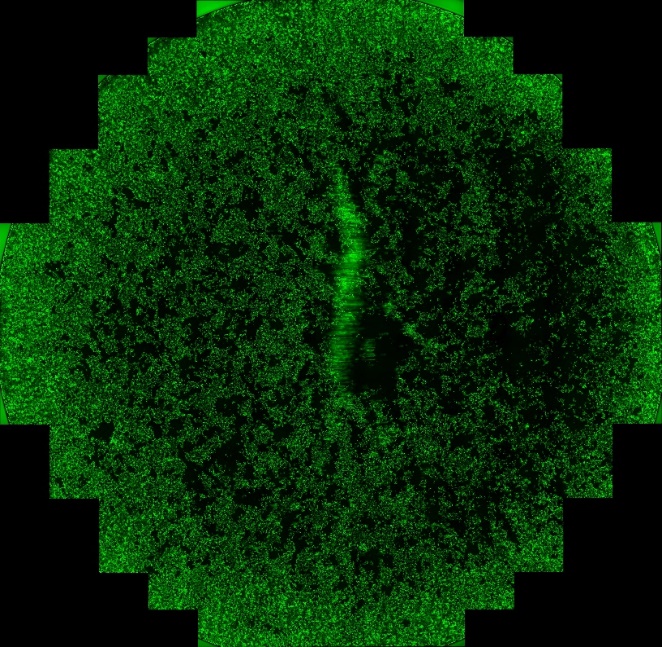
**

**Figure 6E**

| Treatment | dpi | percentage |
| --- | --- | --- |
| nb2-4 | d1 | 5,993691 |
| nb2-4 | d1 | 5,915904 |
| nb2-4 | d1 | 5,683646 |
| nb2-19 | d1 | 7,491857 |
| nb2-19 | d1 | 4,94671 |
| nb2-19 | d1 | 6,046274 |
| nbctrl | d1 | 9,780262 |
| nbctrl | d1 | 9,808853 |
| nbctrl | d1 | 9,67905 |
| nb2-4 | d2 | 11,83404 |
| nb2-4 | d2 | 12,54826 |
| nb2-4 | d2 | 12,0564 |
| nb2-19 | d2 | 11,11853 |
| nb2-19 | d2 | 11,56118 |
| nb2-19 | d2 | 11,70882 |
| nbctrl | d2 | 37,24871 |
| nbctrl | d2 | 39,46882 |
| nbctrl | d2 | 38,42099 |
| nb2-4 | d3 | 30,66759 |
| nb2-4 | d3 | 32,03906 |
| nb2-4 | d3 | 32,69433 |
| nb2-19 | d3 | 29,72367 |
| nb2-19 | d3 | 32,13043 |
| nb2-19 | d3 | 31,22543 |
| nbctrl | d3 | 62,86803 |
| nbctrl | d3 | 64,71136 |
| nbctrl | d3 | 63,26079 |
