# Supplementary material for: Herpesviruses mimic zygotic genome activation to promote viral replication
Source: Nat Commun. 2025 Jan 16;16:710. doi: 10.1038/s41467-025-55928-5 (PMC11735616; doi:10.1038/s41467-025-55928-5)
Supplement: Supplementary file 14 — Source Data [file 41467_2025_55928_MOESM14_ESM.zip › Figure 5.docx]

**Figure 5A**

**
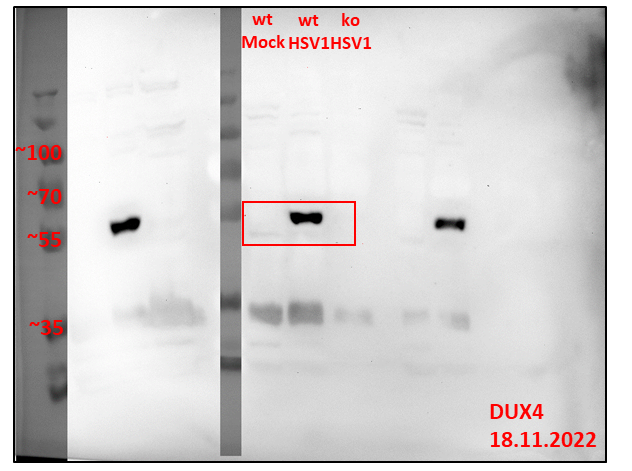
**


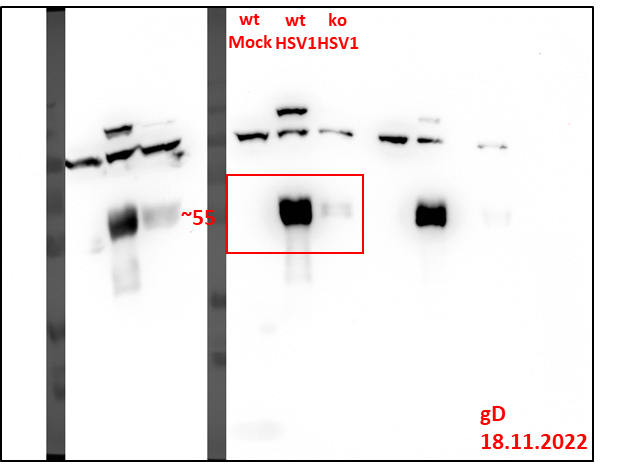


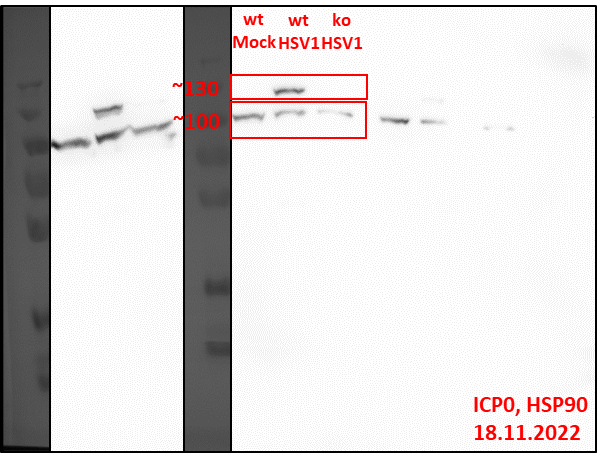


**Figure 5D**

Upper Graph

| Days post infection | HAP1 WT | | | HAP1 KO | | |
| --- | --- | --- | --- | --- | --- | --- |
| *0** | *3,64** | *3,42** | *2,87** | *1,96** | *1,94** | *1,92** |
| 1 | 32,5 | 29,7 | 29 | 21,2 | 18,9 | 22,5 |
| 2 | 83,2 | 87,1 | 83 | 20 | 21,6 | 22,4 |
| 3 | 96,1 | 96 | 96,8 | 18,3 | 17,9 | 18,4 |
| 4 | 96,6 | 97,7 | 97,7 | 32,1 | 28,1 | 25,3 |

Lower Graph

| Days post infection | HAP1 WT | | | DUX4 KO | | |
| --- | --- | --- | --- | --- | --- | --- |
| 0 | 640 | 420 | 560 | 560 | 720 | 960 |
| 1 | 4600 | 4200 | 4200 | 620 | 640 | 700 |
| 2 | 700000 | 540000 | 580000 | 6400 | 8000 | 13000 |
| 3 | 2800000 | 3200000 | 2600000 | 11800 | 8200 | 14000 |
| 4 | 4400000 | 4200000 | 2600000 | 11800 | 12800 | 15000 |

**Figure 5E**

Upper graph

| Days post infection | HAP1 WT | | | HAP1 KO | | |
| --- | --- | --- | --- | --- | --- | --- |
| *0* | 0,82 | 0,87 | 0,85 | 1,59 | 0,69 | 1,66 |
| 1 | 5,92 | 7,03 | 9,56 | 4,74 | 5,57 | 6,36 |
| 2 | 18,2 | 22,1 | 19,3 | 7,87 | 7,42 | 6,86 |
| 3 | 46,9 | 46,6 | 48,2 | 7,51 | 8,3 | 7,58 |
| 4 | 68,9 | 75,2 | 71,9 | 9,82 | 10,8 | 10 |

Lower graph

| Days post infection | WT HAP1 | | | DUX4 KO | | |
| --- | --- | --- | --- | --- | --- | --- |
| 0 | 620 | 500 | 480 | 720 | 620 | 660 |
| 1 | 180 | 260 | 280 | 20 | 0 | 0 |
| 2 | 24000 | 12000 | 10400 | 440 | 380 | 500 |
| 3 | 140000 | 160000 | 240000 | 1800 | 1800 | 1600 |
| 4 | 220000 | 220000 | 140000 | 800 | 1200 | 1100 |

**Figure 5F**

DUX4 KO HSV1

DUX4 KO mock


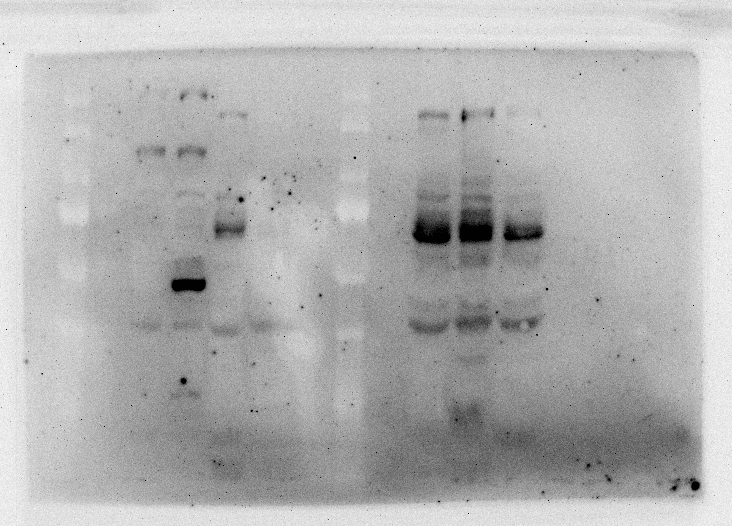


DUX4 KO HSV1

HSV-1

Mock

DUX4

15.08.2018.2019

DUX4 KO mock


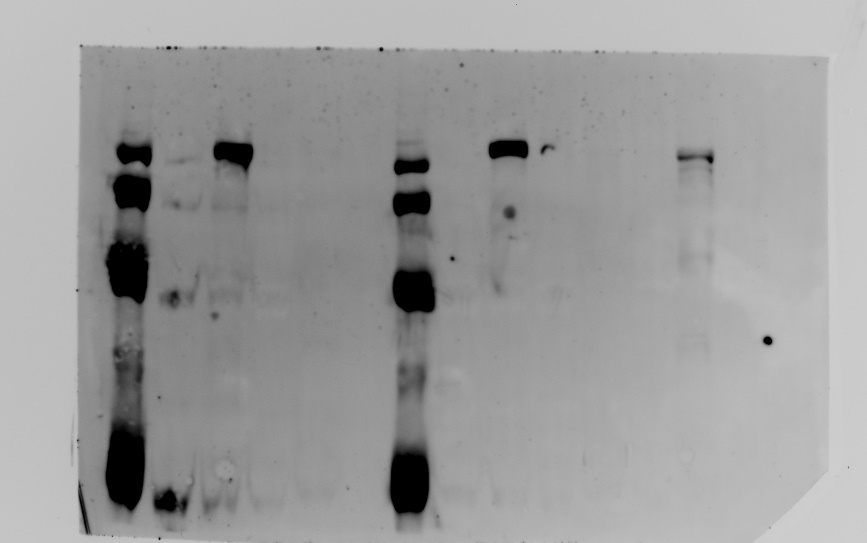


HSV-1

Mock

ICP4

30.11.2018.2019


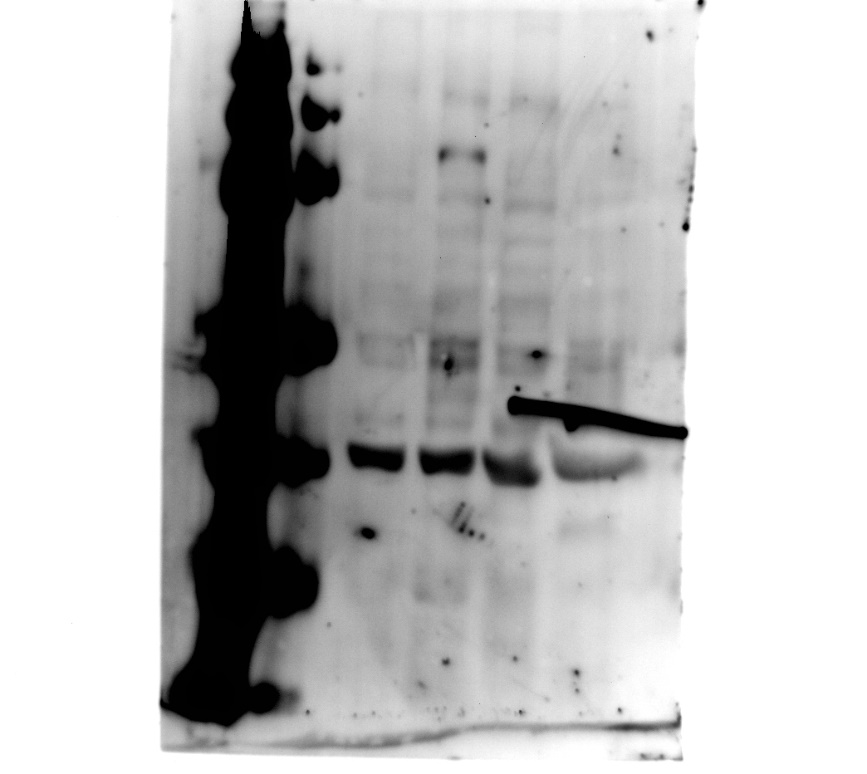


DUX4 KO HSV1

DUX4 KO mock

HSV-1

Mock

ICP0

17.08.2018


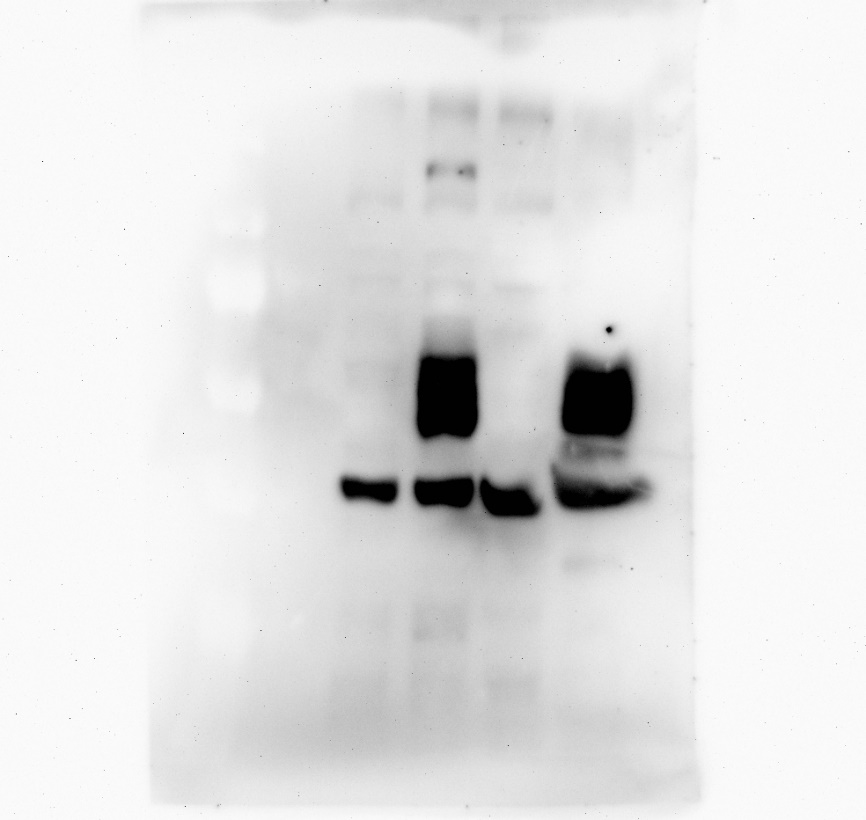


DUX4 KO HSV1

DUX4 KO mock

HSV-1

Mock

Actin

24.08.2018


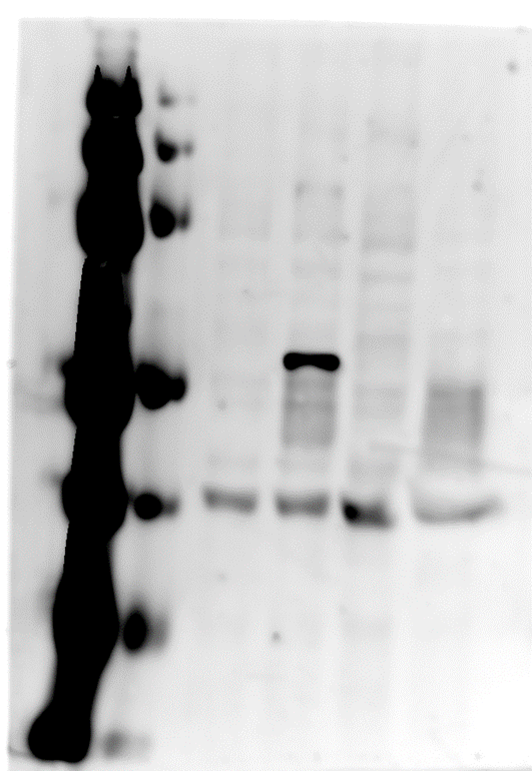


Mock

HSV-1

DUX4 KO mock

DUX4 KO HSV1

VP16


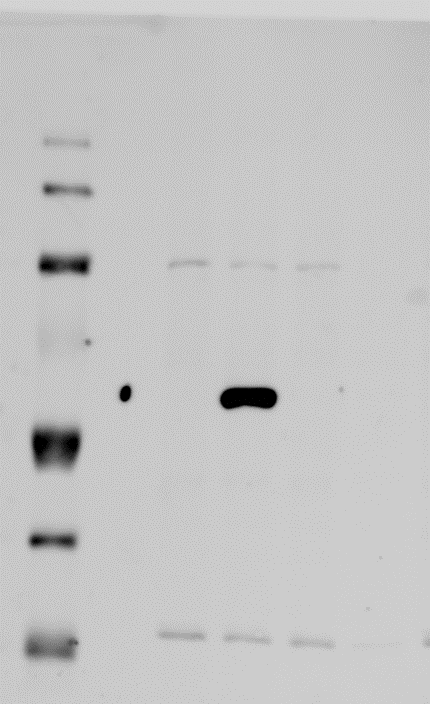


DUX4 KO HSV1

DUX4 KO mock

HSV-1

Mock

ICP27

**Figure 5G**

Left Graph

| hpi | WT GFP | | | KO GFP | | |
| --- | --- | --- | --- | --- | --- | --- |
| 0 | 359 | 379 | 446 | 189 | 173 | 187 |
| 6 | 2360 | 2152 | 2718 | 472 | 414 | 382 |
| 12 | 15027 | 19821 | 20230 | 2800 | 1635 | 2674 |

Right Graph

| hpi | WT KOS | | | KO KOS | | |
| --- | --- | --- | --- | --- | --- | --- |
| 0 | 377 | 559 | 552 | 287 | 300 | 285 |
| 6 | 17341 | 17183 | 14427 | 3029 | 3053 | 3973 |
| 12 | 112892 | 111781 | 106772 | 31005 | 37113 | 41495 |

**Figure 5H**

|  | ICP0 | | |
| --- | --- | --- | --- |
| 2,6nM | 1,1448016 | 0,456587409 | 0,326405385 |
| 9,8nM | 1,041 | 0,152808247 | 0,133244604 |
| DMSO | 1 |  |  |

|  | ICP27 | | | TRIM43 | | |
| --- | --- | --- | --- | --- | --- | --- |
| 2,6nM | 1,1973317 | 0,233655651 | 0,195503697 | 0,0622935 | 0,008311356 | 0,007332973 |
| 9,8nM | 1,1380698 | 0,040068652 | 0,038705912 | 0,0524161 | 0,012192049 | 0,009891315 |
| DMSO | 1 |  |  | 1 |  |  |
