# Supplementary material for: Herpesviruses mimic zygotic genome activation to promote viral replication
Source: Nat Commun. 2025 Jan 16;16:710. doi: 10.1038/s41467-025-55928-5 (PMC11735616; doi:10.1038/s41467-025-55928-5)
Supplement: Supplementary file 14 — Source Data [file 41467_2025_55928_MOESM14_ESM.zip › Figure 2.docx]

**Figure 2A**


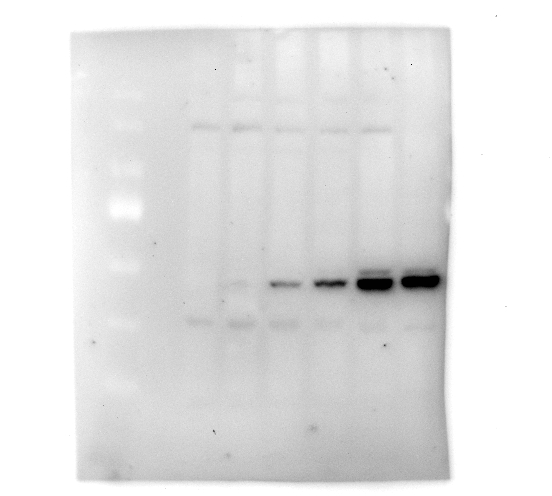


Mock

4 hpi

6 hpi

8 hpi

12 hpi

24 hpi

DUX4

25.02.2019


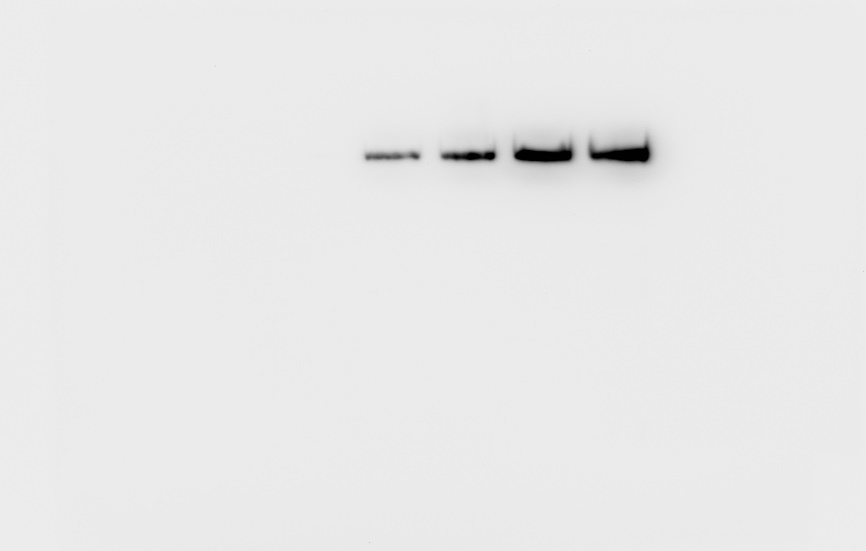


ICP0

15.05.2019

24 hpi

12 hpi

8 hpi

6 hpi

4 hpi

Mock


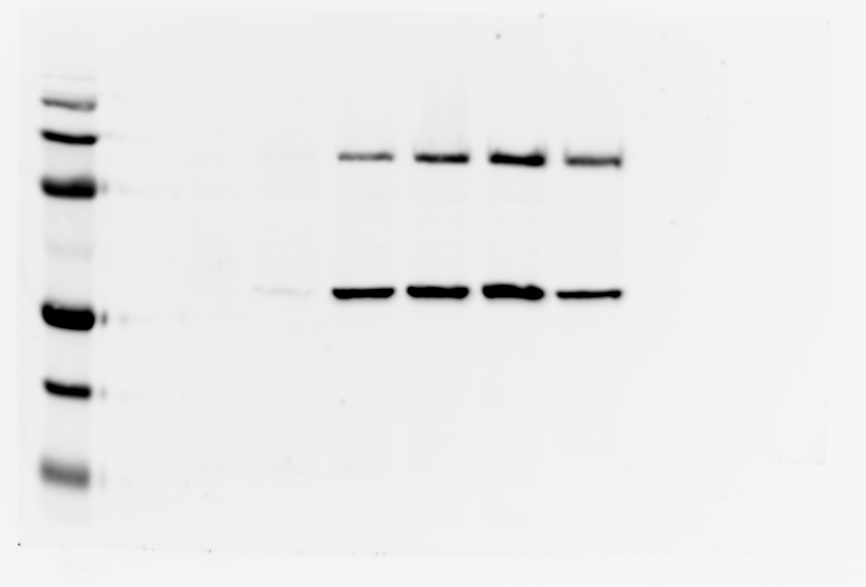


ICP27

16.05.2019

Mock

4 hpi

6 hpi

8 hpi

12 hpi

24 hpi


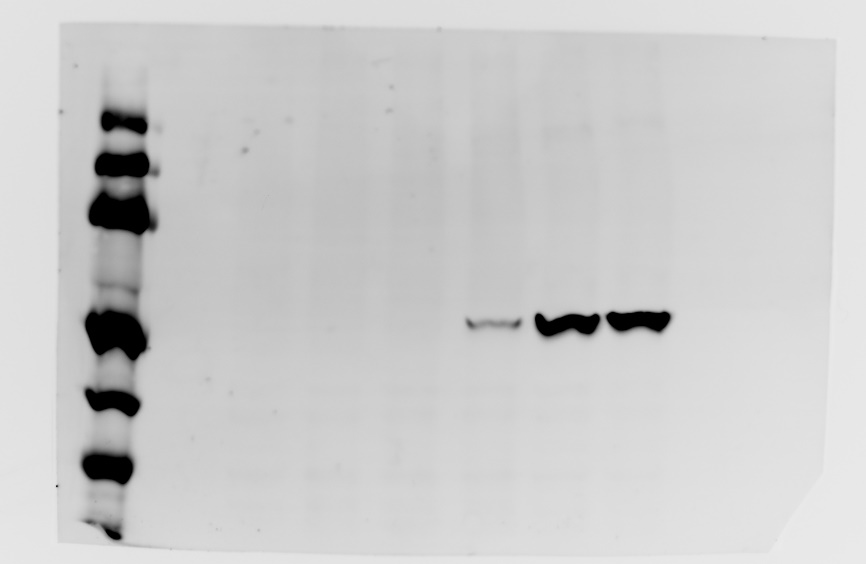


24 hpi

12 hpi

8 hpi

6 hpi

4 hpi

Mock

VP16

08.03.2019


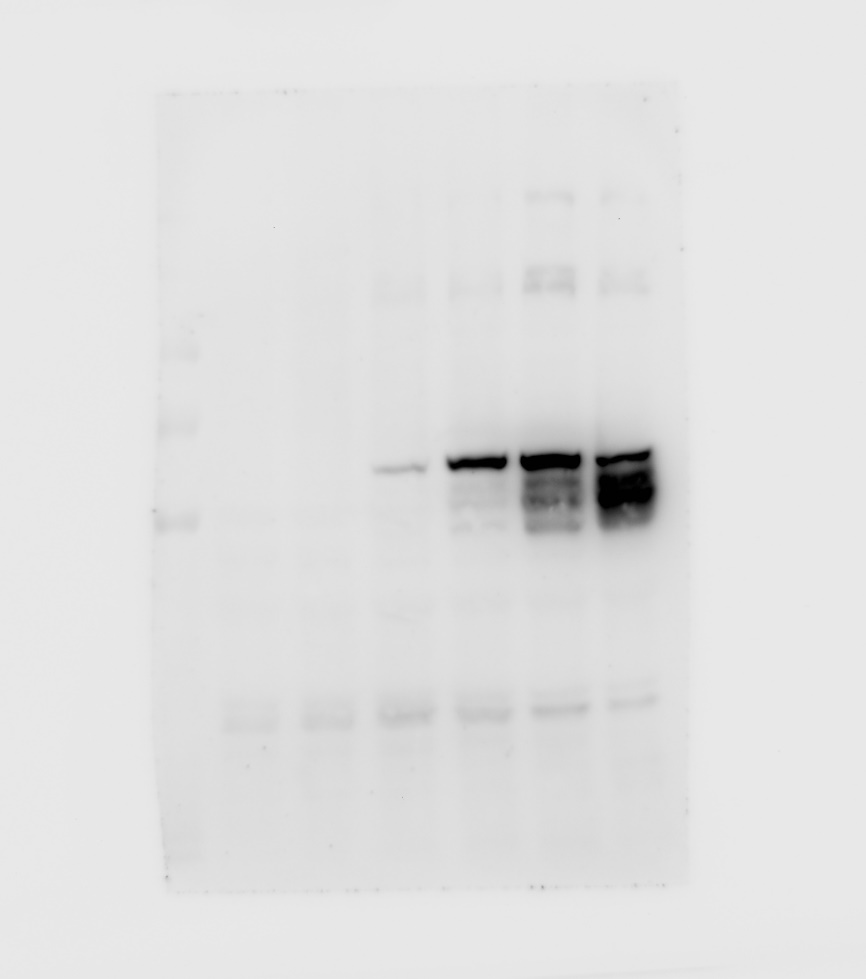


Mock

4 hpi

6 hpi

8 hpi

12 hpi

24 hpi

gD

28.02.2019


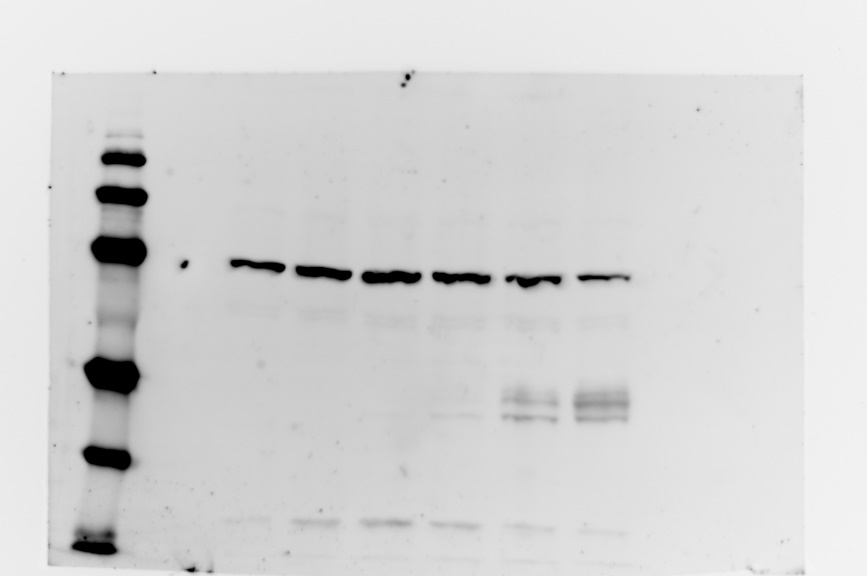


HSP90

17.05.2019

Mock

4 hpi

6 hpi

8 hpi

12 hpi

24 hpi

**Figure 2B**

| h.p.i. | wt | | | dvhs | | |
| --- | --- | --- | --- | --- | --- | --- |
| 0 | 0 | 0 |  | 0 | 0 |  |
| 1 | 0 | 0,05636 |  | 0,04 | 0,03 |  |
| 2 | 0,2371 | 0,05582 |  | 0,07 | 0,07 |  |
| 3 | 1,877 | 1,214 |  | 3,32 | 2,4 |  |
| 4 | 2,652 | 3,257 |  | 6 | 5,36 |  |
| 5 | 2,694 | 2,601 |  | 4,79 | 5,52 |  |
| 6 | 1,779 | 1,986 |  | 3,06 | 3,04 |  |
| 7 | 1,233 | 0,6641 |  | 1,8 | 2,05 |  |
| 8 | 0,6223 | 0,7535 |  | 0,94 | 0,96 |  |

**Figure 2C**

HSV1 dICP4

HSV1 WT

HSV1 dICP34.5

HSV1 dICP0

HSV1 WT


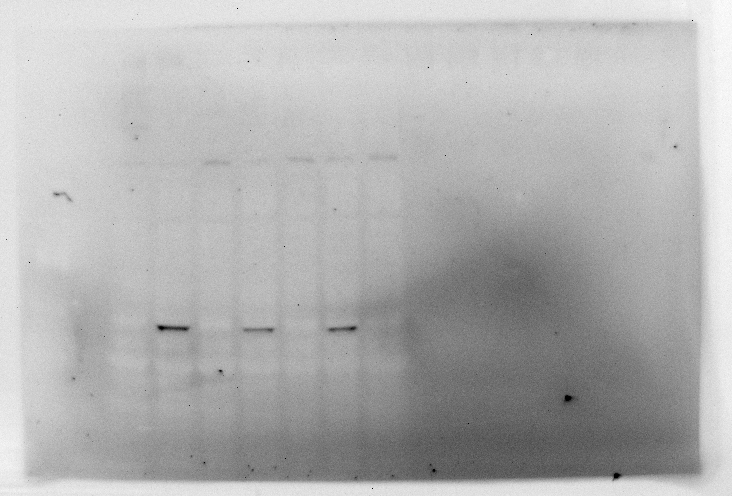


Mock

Mock

DUX4

31.10.2019


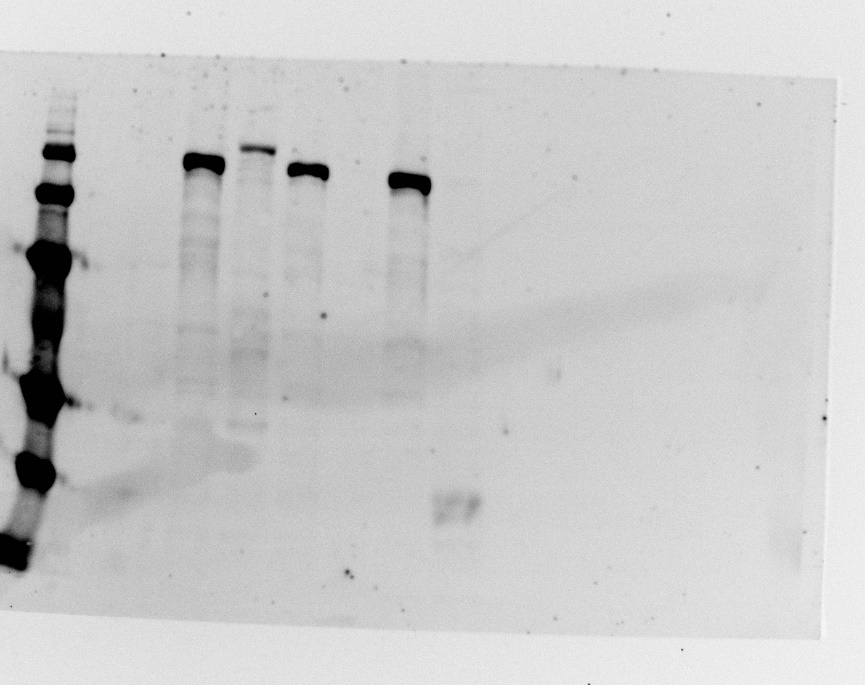


ICP4

19.11.2019.2019

HSV1 dICP4

HSV1 WT

Mock

HSV1 dICP34.5

HSV1 dICP0

HSV1 WT

Mock

HSV1 WT

Mock

HSV1 dICP34.5

HSV1 dICP0

HSV1 WT

Mock

HSV1 dICP4


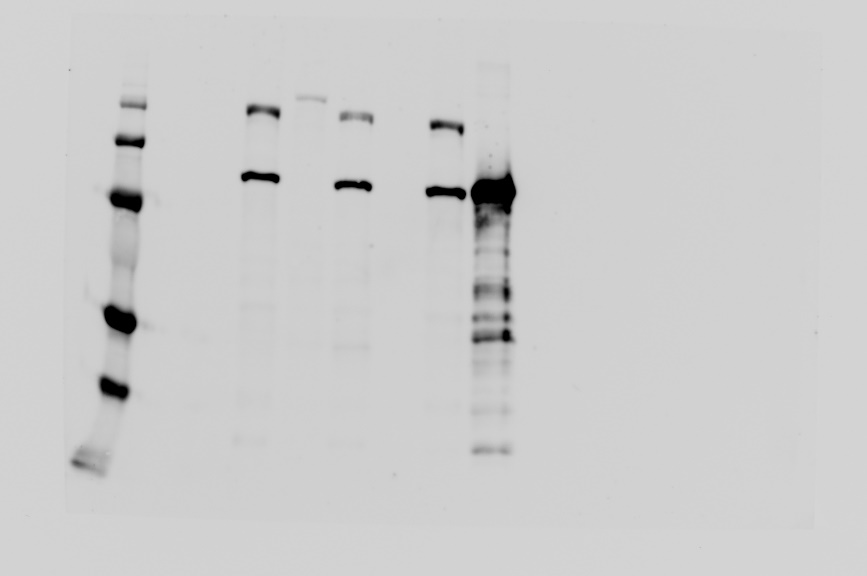


ICP0

20.11.2019.2019


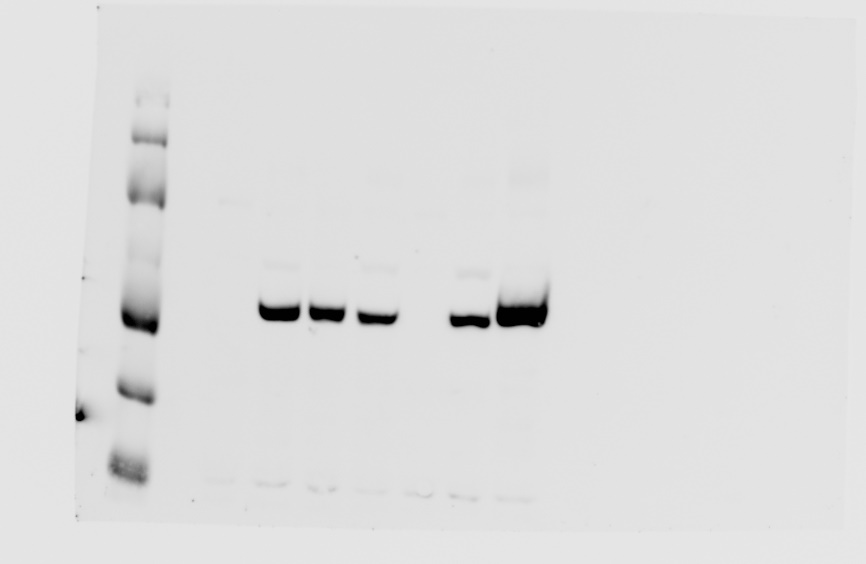


ICP27

03.12.2019.2019

Mock

HSV1 WT

HSV1 dICP0

HSV1 dICP34.5

Mock

HSV1 WT

HSV1 dICP4


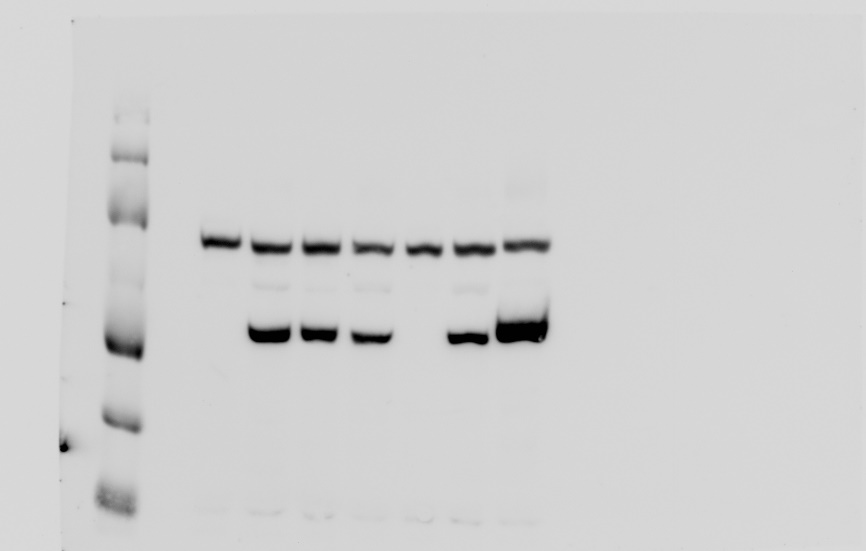


HSP90

04.12.2019.2019

Mock

HSV1 WT

HSV1 dICP0

HSV1 dICP34.5

Mock

HSV1 WT

HSV1 dICP4

ICP0

20.11.2019

**Figure 2D**


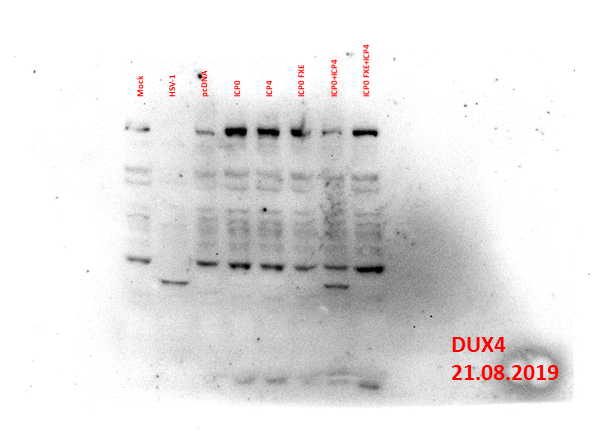


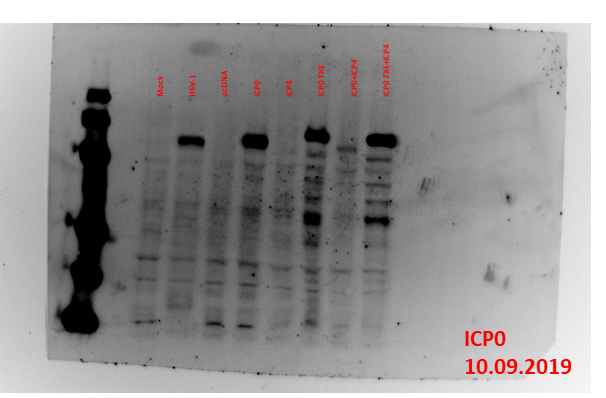


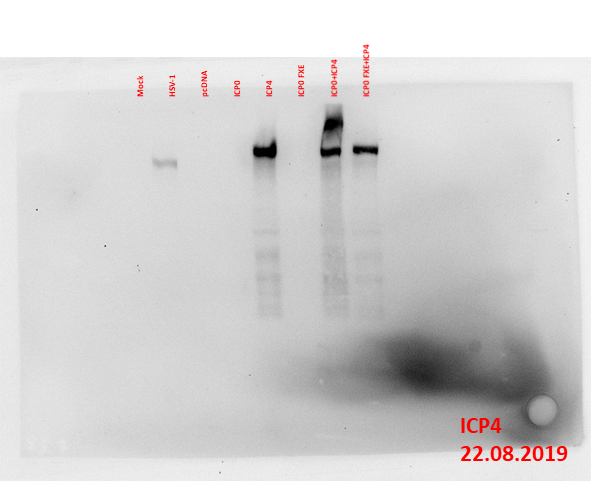


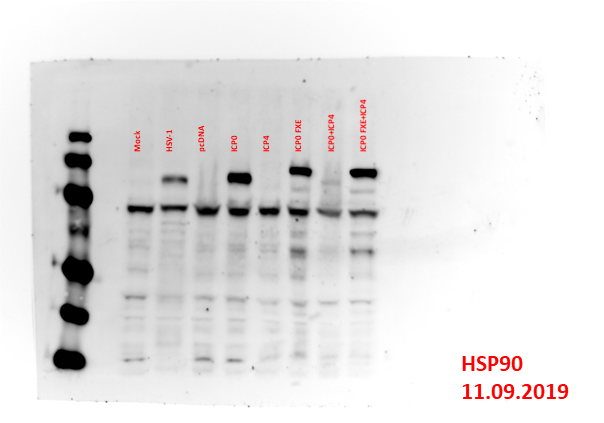


**Figure 2E**

|  |  | **ICP0** | | |
| --- | --- | --- | --- | --- |
| **mock** |  | **1** | **32** | **1** |
| **HSV-1** |  | **1383604** | **1763488** | **1691650** |
| **HSV-1 PAA** |  | **18305,63** | **18561,17** | **18053,61** |

|  |  | **DUX4** | | |
| --- | --- | --- | --- | --- |
| **mock** |  | **1** | **1** | **1** |
| **HSV-1** |  | **7,835362** | **8,456144** | **8,168097** |
| **HSV-1 PAA** |  | **37,01402** | **44,01734** | **37,79177** |

|  |  | **TRIM43** | | |
| --- | --- | --- | --- | --- |
| **mock** |  | **1** | **1** | **1** |
| **HSV-1** |  | **248,9997** | **310,8339** | **290,0183** |
| **HSV-1 PAA** |  | **9089,593** | **9152,816** | **9541,498** |

|  |  | **gC** | | |
| --- | --- | --- | --- | --- |
| **mock** |  | **1** | **1** | **1** |
| **HSV-1** |  | **18305,63** | **751805,5** | **912838,4** |
| **HSV-1 PAA** |  | **229,1264** | **207,9366** | **209,3829** |
