# Supplementary figures and images for: Herpesviruses mimic zygotic genome activation to promote viral replication
Source: Nat Commun. 2025 Jan 16;16:710. doi: 10.1038/s41467-025-55928-5 (PMC11735616; doi:10.1038/s41467-025-55928-5)

**Figure 1A**

**
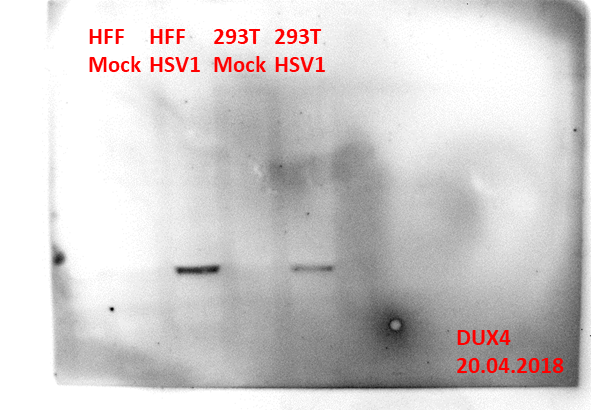
**

**
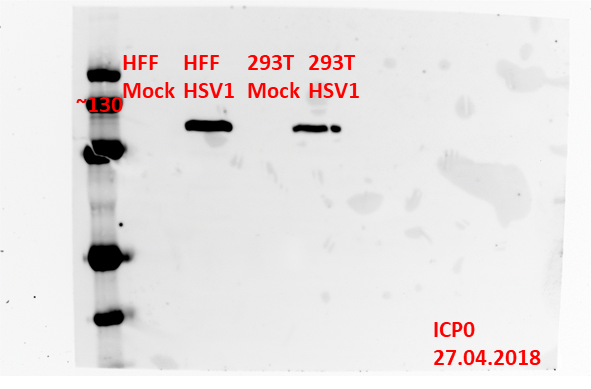
**

**
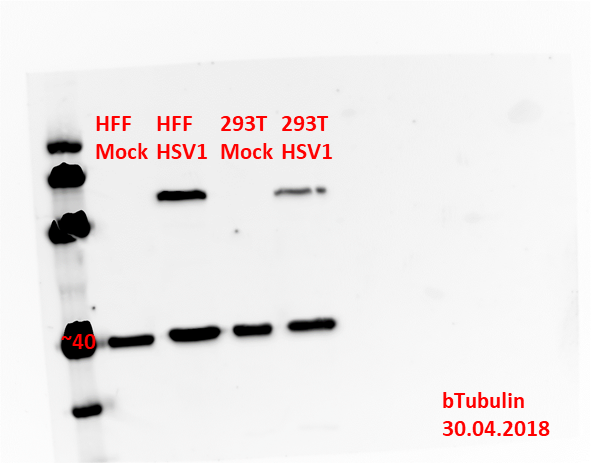
**

**Figure 1C**


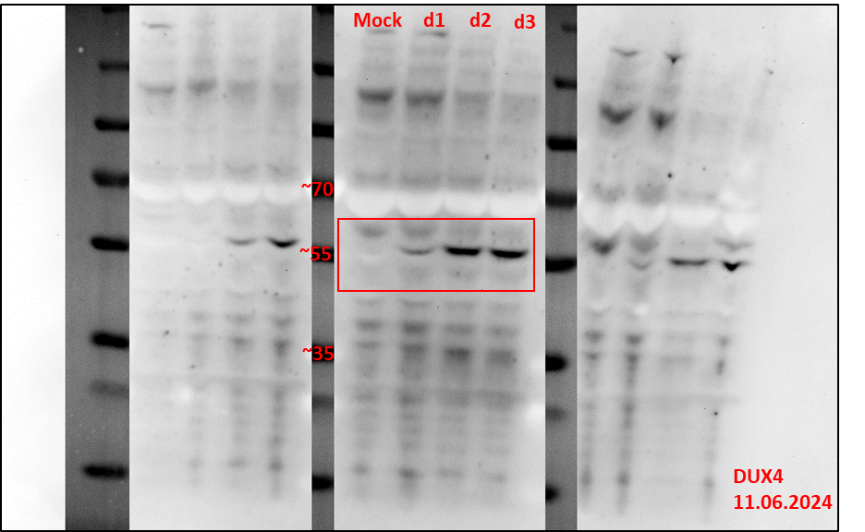


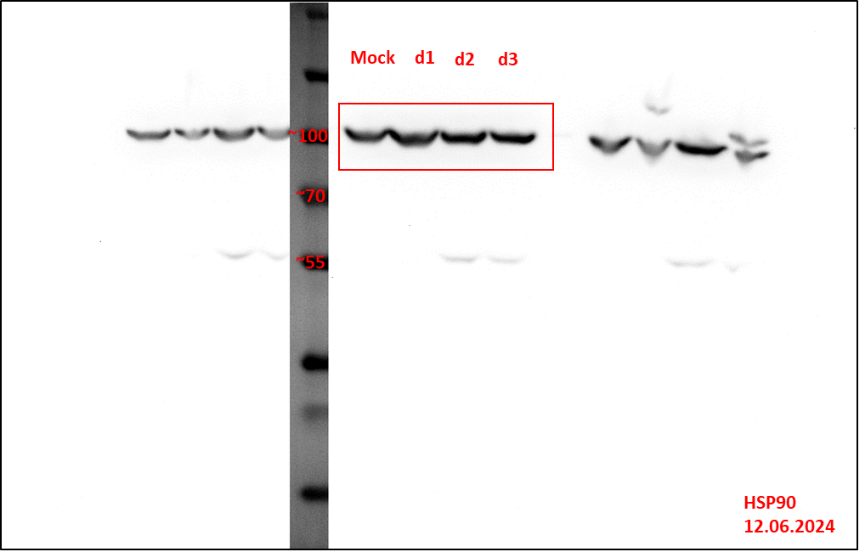


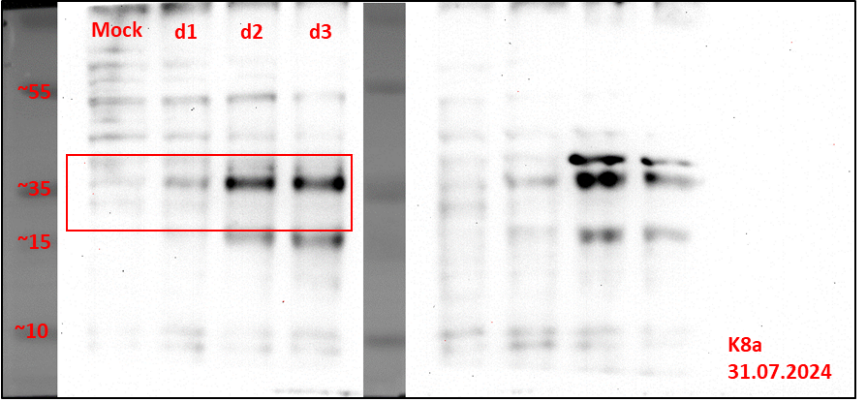


**Figure 1D**

293T mock

293T HSV-1


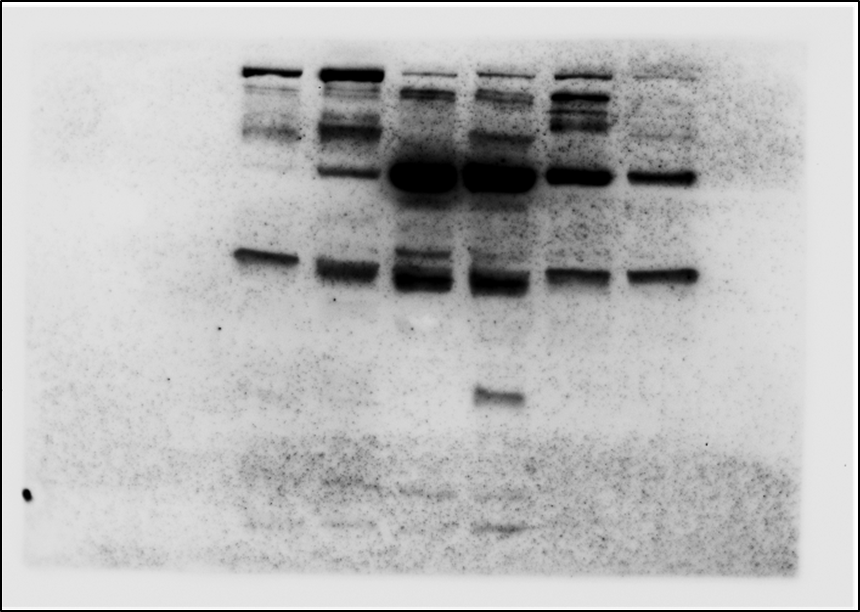


ZSCAN4


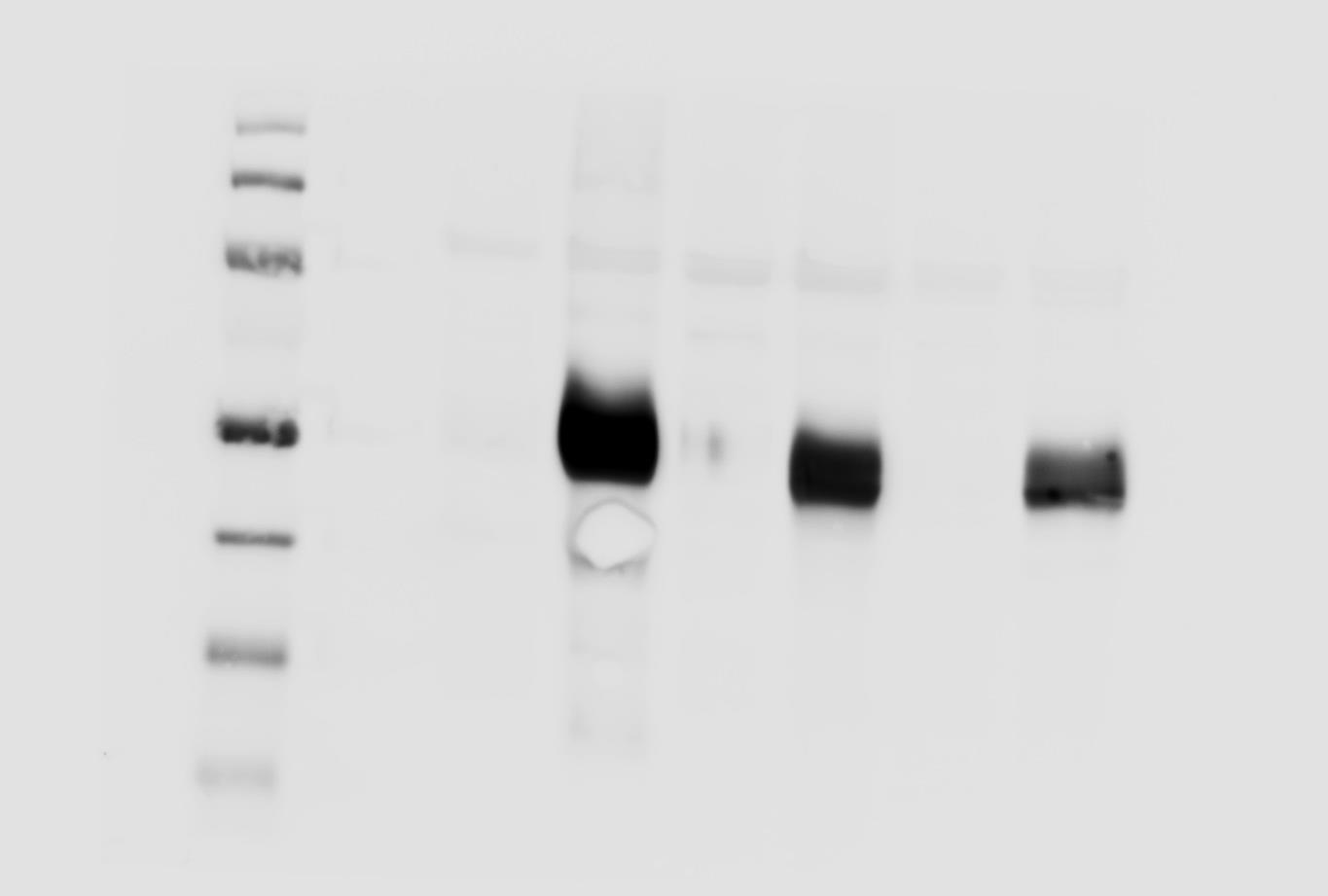


293T mock

293T HSV-1

gD


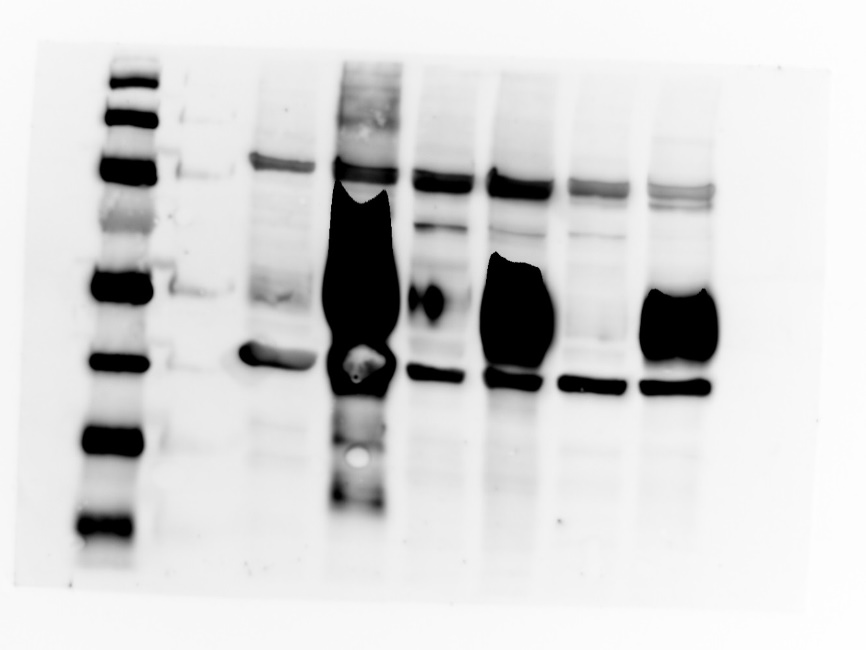


293T mock

293T HSV-1

ß-Actin

**Figure 1E**

**
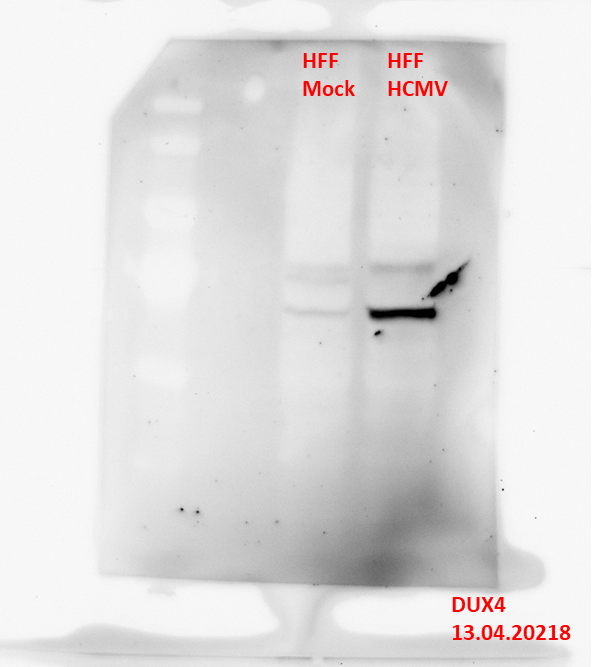
**

**
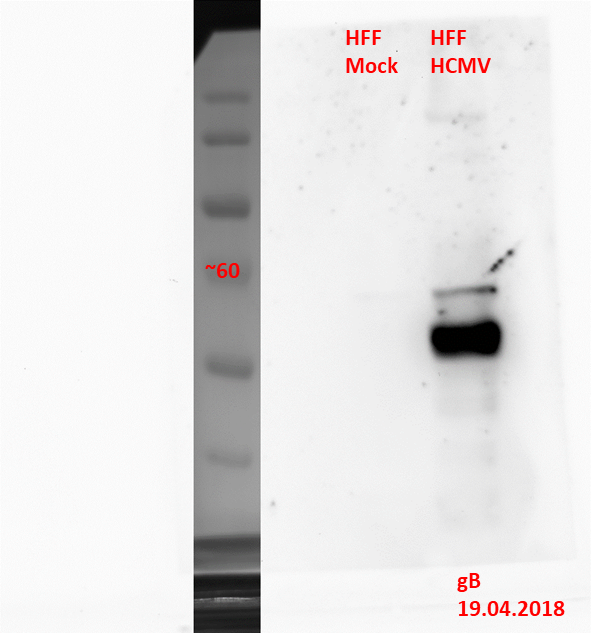
**

**
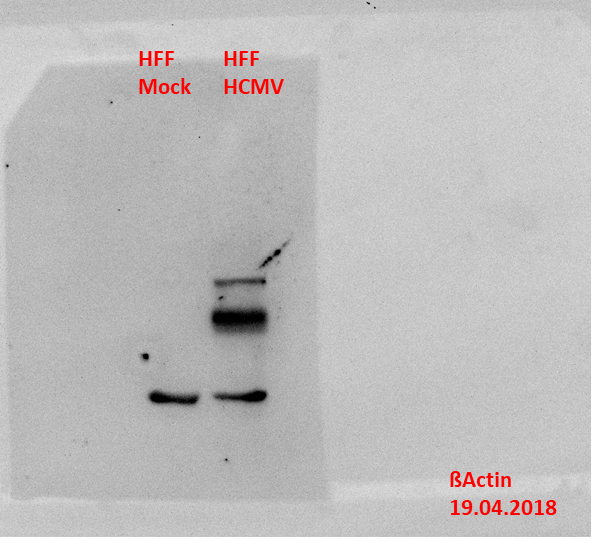
**

Supplement: Supplementary file 14 — Source Data [file 41467_2025_55928_MOESM14_ESM.zip › Figure 1.docx]

**Supplemental Figure 6B**

**
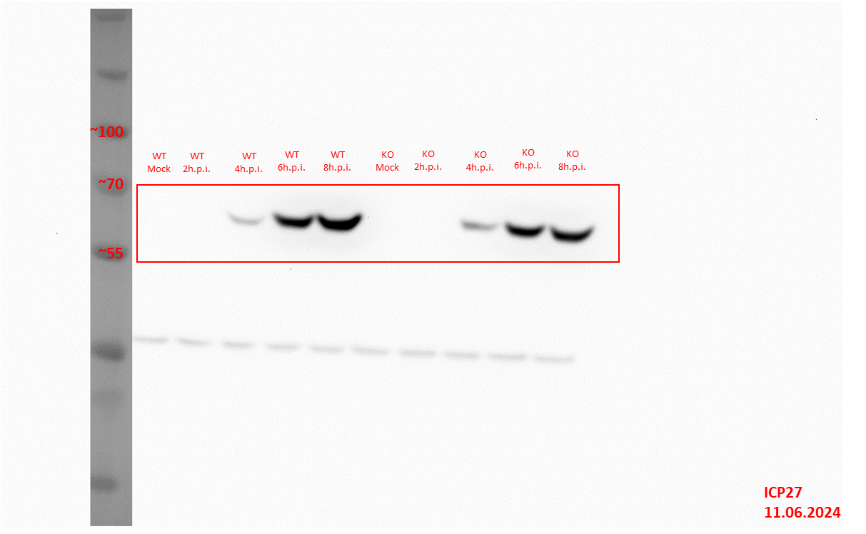
**

**
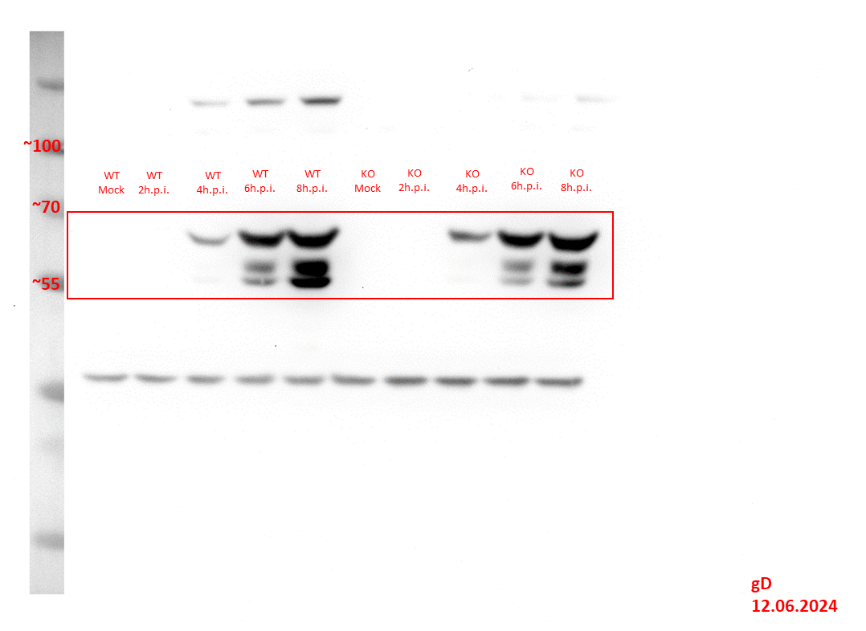
**

**
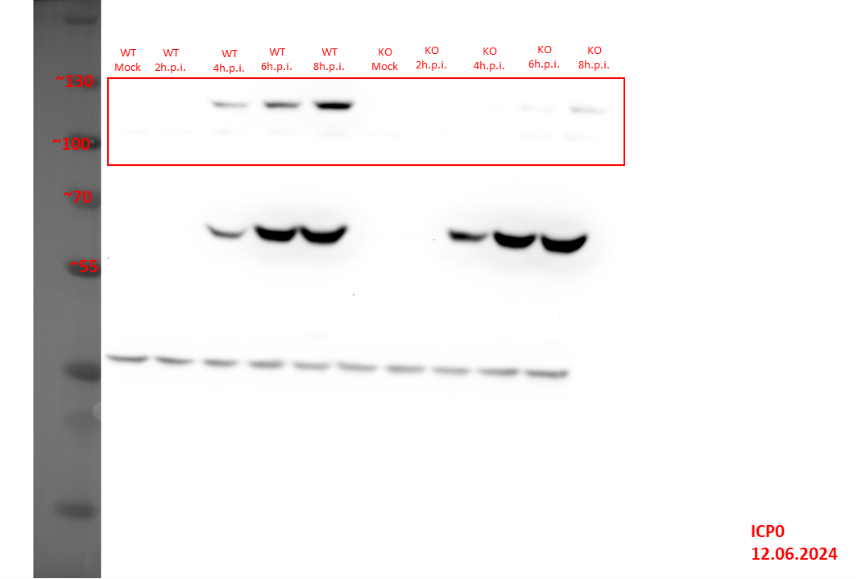
**

**
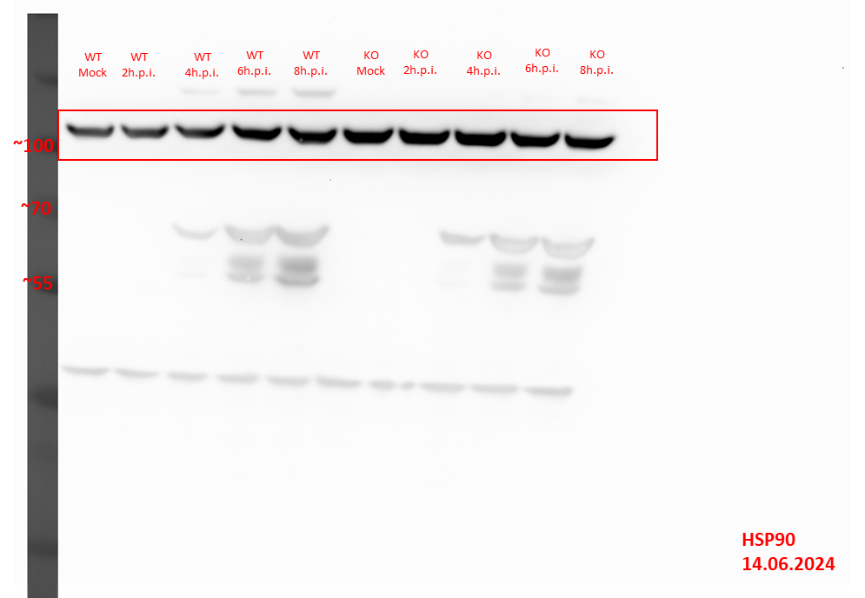
**

Supplement: Supplementary file 14 — Source Data [file 41467_2025_55928_MOESM14_ESM.zip › Supplemental Figure 6.docx]

**Figure 3D**

**
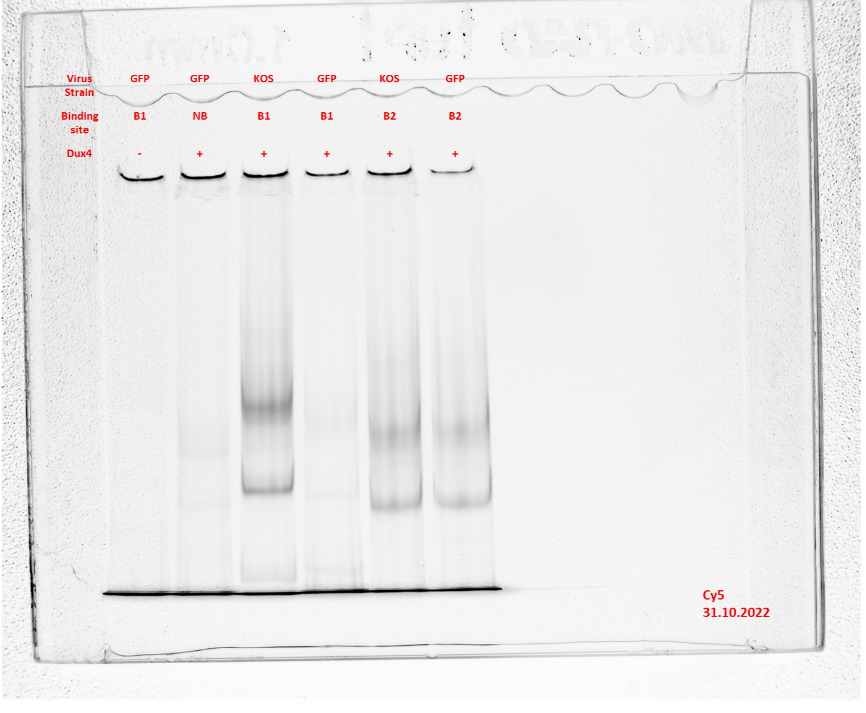
**

Supplement: Supplementary file 14 — Source Data [file 41467_2025_55928_MOESM14_ESM.zip › Figure 3.docx]
